# Benzyl thioether formation merging copper catalysis†

**DOI:** 10.1039/d1ra08015f

**Published:** 2021-12-23

**Authors:** Bing Xu, Ying Lin, Yang Ye, Li Xu, Tian Xie, Xiang-Yang Ye

**Affiliations:** School of Pharmacy, Hangzhou Normal University Hangzhou Zhejiang 311121 PR China yangye@hznu.edu.cn; Key Laboratory of Elemene Class Anti-Cancer Chinese Medicines, Engineering Laboratory of Development and Application of Traditional Chinese Medicines, Collaborative Innovation Center of Traditional Chinese Medicines of Zhejiang Province, Hangzhou Normal University Hangzhou Zhejiang 311121 PR China

## Abstract

A novel copper-catalyzed thioetherification reaction has been developed to afford benzyl thioethers in moderate to excellent yields. Under the mild and easy-to-operate conditions, a variety of thioethers are efficiently prepared from readily available benzyl alcohols (primary, secondary, and tertiary) and thiols in the presence of Cu(OTf)_2_ as the Lewis acid catalysis. This C–S bond formation protocol furnishes exceptional chemoselectivity, and the preliminary mechanism studies show that the reaction should proceed through a Lewis-acid-mediated S_N_1-type nucleophilic attack of the carbocations formed *in situ*.

## Abstract 2

A novel copper-catalyzed thioetherification reaction has been developed to afford benzyl thioethers in moderate to excellent yields.

## Graphical abstract

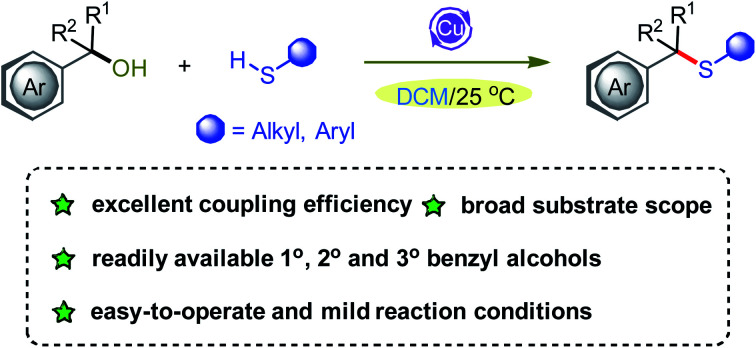

## Introduction

**Scheme 1:**
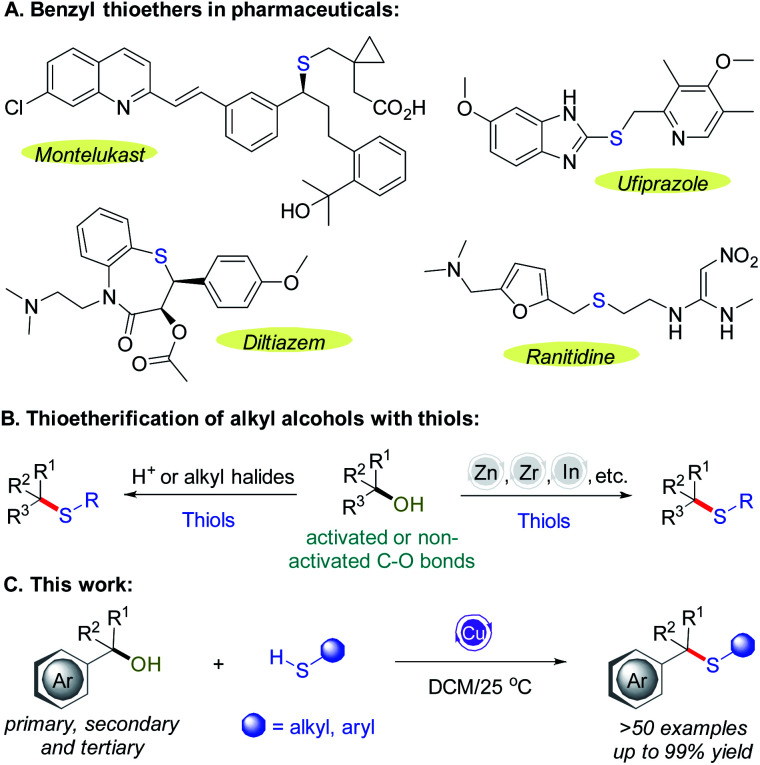
Benzyl thioethers and thioetherification reactions.

Sulfur compounds often show different biological activities and have important application value in the pharmaceutical industry.^1^ Thioethers are a type of sulfur-containing compound with diverse physiological activities^2^ and unique physicochemical properties.^3^ As an important structural unit, thioethers widely exist in natural products,^4^ drugs^2,5^ and organic functional materials.^3*a*,6^ For example (Scheme 1A), montelukast^7^ is a selective leukotriene receptor antagonist approved for oral treatment of asthma and allergic rhinitis. Ufiprazole^8^ can be used to treat acid related diseases such as peptic ulcer and gastroesophageal reflux disease. Diltiazem^9^ is a safe and effective drug for the treatment of supraventricular arrhythmia, angina pectoris and hypertension in the elderly. Ranitidine^10^ is a digestive system drug used to relieve stomachache, heartburn and acid reflux caused by excessive gastric acid. In addition, studies have shown that thioethers can also be used as biopheromones for animal communication.^11^

In view of the significant and broad potential applications of thioethers, chemists have developed a series of methods to construct thioethers based on the alkyl alcohol substrates,^12,18–20^ which constitute an important part of our chemical feedstocks.^13–15^ A variety of sulfide compounds were synthesized through these methods,^16^ but the disadvantages of the traditional methods are usually accompanied that can not be ignored,^17^ such as largely they require harsh or strong acidic conditions to stimulate the reaction of alkyl alcohols with thioalcohols or thiophenols,^18^ and the reaction have poor step economy and atomic economy. These shortcomings limit the applications of these reactions to a certain extent. Recently, Lewis acid and transition metal catalyzed construction of thioethers using activated or non-activated alkyl alcohols have been greatly developed,^19,20^ which often have the following characteristics compared with the traditional synthesis methods: (1) higher atomic economy; (2) mild and easy-to-operate manners; (3) shorter synthesis steps and less-waste generation. Therefore, some examples have been discretely reported for the thiolation of benzyl alcohols under Lewis acid catalyzed reaction conditions, such as Zn-, Zr-, In-, Fe-complexes, *etc.* (Scheme 1B)^20^ However, there is still a great demand for the preparation of benzyl thioethers from readily accessible alcohol precursors under more available and mild catalytic conditions.

From this initiative, we explored a process for thioetherification of benzyl alcohols with thiols catalyzed by a Cu-complex with intriguing results (Scheme 1C). More specifically, most reactions were highly chemoselective with near equimolar quantities of the products, and occurred at mild temperature. Multiple functional groups were well tolerated. The preliminary mechanistic studies were also discussed.

## Results and discussion

We initiated our investigation by exploring the thioetherification of 2-phenylpropan-2-ol 1 with 4-fluorobenzenethiol 2 as the model substrates (Table 1).^21^ An extensive screening of the reaction parameters revealed that the use of Cu(OTf)_2_ (3 mol%) in DCM (dichloromethane) at an air atmosphere of 25 °C delivered benzyl sulfide product 3 in 96% yield (entry 1). Under the selected conditions, Lewis acids appeared to have great influence on this C–S bond transformation. After the screening for Cu sources, Cu(OTf)_2_ was the best copper catalyst for this thioetherification reaction (Table S5†).^21^ Without Cu(OTf)_2_, the reaction could not afford 3 (entry 2). Next, the amount of Cu(OTf)_2_ was briefly screened in Table 1, the use of 1 mol% Cu(OTf)_2_ generated 3 in a low yield (entry 3) and when added more Cu(OTf)_2_ could produce 3 in reasonable high yields (entries 4 and 5), whereas replacing Cu(OTf)_2_ with other Lewis acids did not result in better outcomes (entries 6–8). Replacement of DCM by DCE (1,2-dichloroethane) led to an inferior yield, that was shown to be an unsuitable solvent (entry 9). Reaction at 50 °C (entry 11) obtained a comparable yield, while somewhat lower yields were obtained at 0 °C and 80 °C (entries 10 and 12). Change of other parameters such as the amount of the substrates were also have an impact on this transformation (entries 13 and 14).

**Table 1:** Optimization for the formation of 3

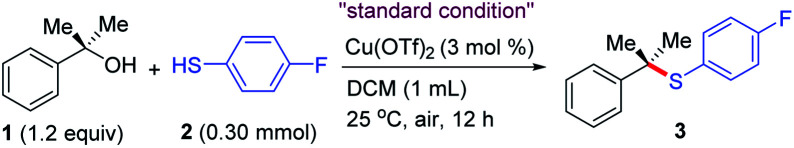		
Entry	Variation from standard conditions^a^	Yield^b^ [%]
1	None	96
2	W/o Cu(OTf)_2_	No reaction
3	Cu(OTf)_2_ (1 mol%)	65
4	Cu(OTf)_2_ (5 mol%)	99
5	Cu(OTf)_2_ (8 mol%)	99
6	Ni(OTf)_2_ instead of Cu(OTf)_2_	61
7	Zn(OTf)_2_ instead of Cu(OTf)_2_	15
8	Sc(OTf)_2_ instead of Cu(OTf)_2_	44
9	DCE instead of DCM	91
10	0 °C	34
11	50 °C	98
12	80 °C	75
13	1 (0.60 mmol)	84
14	2 (1.5 equiv.)	99

a Standard conditions: 1 (1.2 equiv.), 2 (0.30 mmol, 1.0 equiv.), Cu(OTf)_2_ (3 mol%), DCM (1.0 mL), 25 °C, 12 h.

b Isolated yield (average of 2 independent runs). DCE = 1,2-dichloroethane, DCM = dichloromethane.

**Fig. 1:**
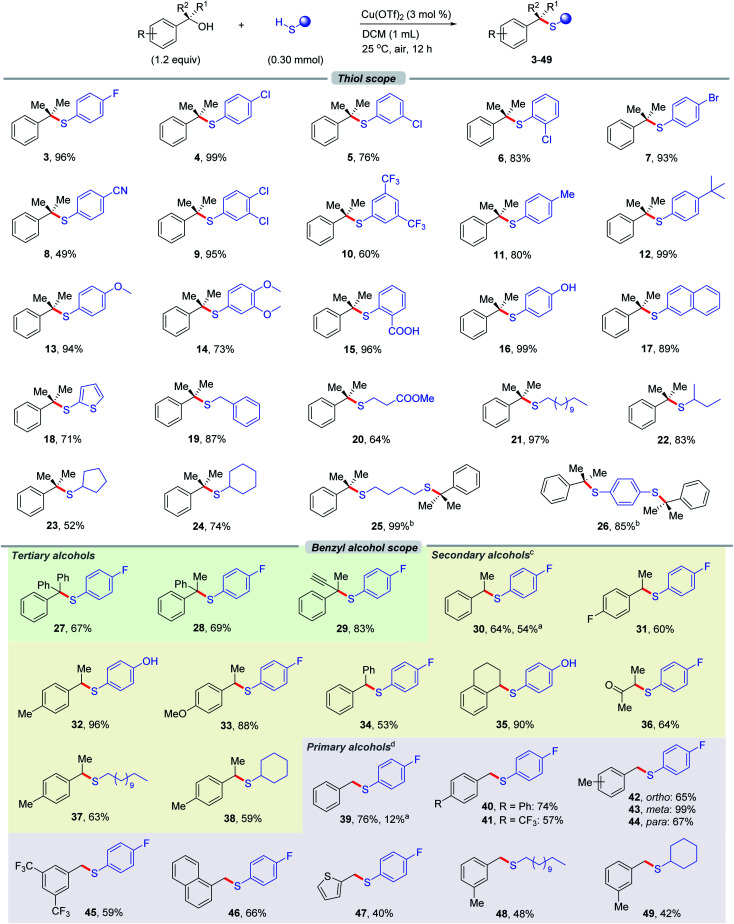
The scope of thiols and benzyl alcohols. ^*a*^ The standard reaction conditions; isolated yields are provided (average of 2 independent runs). ^*b*^ 2-Phenylpropan-2-ol 1 (2.4 equiv.). ^*c*^ Cu(OTf)_2_ (8 mol%), DCE instead of DCM, 40 °C. ^*d*^ Cu(OTf)_2_ (8 mol%), DCE instead of DCM, 80 °C.

With the optimal reaction conditions in hand, we sought to explore the generality of this thioetherification reaction. Firstly, a wide range of thiophenols bearing electron-poor (3–10) or electron-rich substituents (11–14) on the arene afforded the desired products smoothly (Fig. 1). The electronic properties of alkenyl halides did not show an obvious effect on the efficiency of this transformation. Notably, the 4-Cl substituted thiophenol (4) was shown to participate in the reaction to provide better satisfying result than the 2- or 3-positions (5–6). In addition, polysubstituted thiophenols (9–10, 14) were also proved to be compatible. Excellent coupling results with good chemoselectivity were also observed for the substituents on the thiophenols with active hydrogen, such as containing carboxyl or hydroxyl groups or moieties (15–16). It was found that naphthyl- (17) and heteroaromatic-substituted thiophenols such as thiophene (18) were also suitable substrates with good yields. Furthermore, the primary and secondary thioalcohols, as exemplified in 19–24, were effective to couple with 2-phenylpropan-2-ol 1, manifesting that the scope of thioalcohols was broad. The compatible functional groups on the primary thioalcohols include terminal alkane, and ester. The secondary thioalcohols derived from alkane (22) afforded the product in good yield. However, a slight decrease in yields were observed when cyclic alkyl thioalcohols include 5-, and 6-membered rings (23–24) were used as the substrates. No reaction took place when tertiary butyl thiol was subjected to the reaction, which is indicative of the dependence of coupling efficiency on the steric encumbrance. Finally, the thioetherification protocol was extendable to the coupling of symmetrical alkyl or aryl dithioalcohol with 2-phenylpropan-2-ol 1, which furnished 25 and 26 in 99% and 85% yields, respectively. In this case, two sulfur containing quaternary carbon centers were established simultaneously in an efficient manner.

Our attention was then shifted to the scope of the benzyl alcohol partner. As shown in Fig. 1, primary, secondary and tertiary benzyl alcohols bearing a variety of substituents underwent this thioetherification smoothly with gratifying yields. Signally, this Cu-catalyst system coupled a thioalcohol in the presence of different edition of tertiary benzyl alcohols with moderate to high yields (27–29). Under these exceptionally mild reaction conditions, even a sensitive functional group like the alkynyl group (29) remained intact. It was found that secondary benzyl alcohols (30–36) were also suitable substrates. Notably, benzyl alcohols bearing a variety of electron-rich substituents (32–33) such as Me, and MeO on the aromatic ring underwent this transformation efficiently with better yields than electron-deficient substituents. 1,2,3,4-Tetrahydronaphthalen-1-ol (35) could also undergo the reaction, providing access to benzocyclohexane derivative in 90% yield. Moreover, α-hydroxy alcohol derived product (36) could be prepared by this method as well in satisfied result. To further expand the usefulness of this tactic, we attached thioalcohol moiety to a variety of primary benzyl alcohols. Remarkably, all of these substrates bearing electron-poor or -rich substituents include Ph, CF_3_, and Me on the arene examined in our hand underwent the transformation in moderate to excellent yields (39–45). Emphatically, the *meta*-substituted primary benzyl alcohol (43) was shown to participate in the reaction to provide better result than the *ortho*- or *para*-positions (42, 44). Besides, naphthalene (46), and thiophene (47) activated primary alcohols were also competent coupling partners in this reaction system. Moreover, the secondary and primary benzyl alcohols could also reacted with aliphatic thiols (primary and secondary) in moderate yields (37–38, 48–49).

**Fig. 2:**
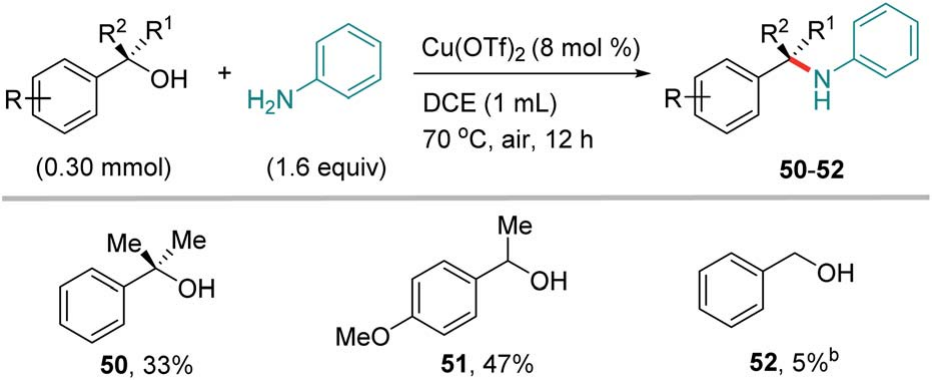
The amination of benzyl alcohols with benzenamine. ^*a*^ The standard reaction conditions; isolated yields are provided (average of 2 independent runs). ^*b*^ Determined by ^1^H NMR using 2,5-dimethylfuran as internal reference.

After an additional screening of the reaction conditions revealed that the use of Cu(OTf)_2_ (8 mol%) in DCE at an air atmosphere of 70 °C that were also applicable for the amination of benzyl alcohols with benzenamine (Fig. 2). Whereas, the amination process was competent with a set of primary, secondary and tertiary benzyl alcohols that delivered the benzyl amines 50–51 in moderate yields, in which a molecular sieve or a base additive was not needed.^22,23^ The stability of carbocation appeared to affect the amination efficiency. For instance, the amination process was not compatible with primary benzyl alcohol 52.1
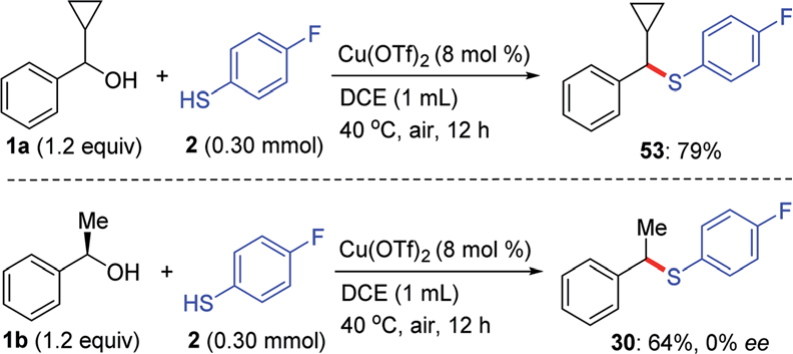


Several mechanism experiments were conducted to get insight into the details on the C–S bond transformation reaction.^21^ Firstly, cyclopropyl-containing alcohol 1a was subjected to the reaction conditions to test whether the C–O bond cleavage event involves formation of alkyl radicals. The cyclopropane-containing product 53 was obtained in 79% yield and didn't accompany by the producing of ring-opening product, which inconsistent with a proposal of the participation of a radical intermediate (eqn (1), top). To further verify the reaction mechanism, the thioetherification of (*R*)-1-phenylethan-1-ol 1b (99% ee, commercial) with 4-fluorobenzenethiol 2 was carried out under the standard condition, giving the thioether 30 in 64% yield constituting a 1 : 1 mixture of enantiomers (eqn (1), bottom). The racemization of the alcohol in the thioetherification event supports a mechanistic scenario that proceeds through a carbocation intermediate. These results reveal that the reaction should proceed through a Lewis acid mediated S_N_1-type nucleophilic attack of *in situ* formed carbocations^24^

## Conclusions

In conclusion, we have developed a Cu-catalyzed coupling of benzyl alcohols with thiols *via* C–O bond cleavage to forge C–S bond. A set of thioethers, significant pharmaceutically interested scaffolds, were efficiently synthesized from easily accessible primary, secondary and tertiary benzyl alcohols and thio-alcohols/phenols in the presence of Cu(OTf)_2_ as the Lewis acid catalysis under mild conditions. Significantly, the present method tolerates a variety of functional groups, affording the coupling products generally in modest to excellent yields. The preliminary mechanistic study indicates that the reaction likely to go through the process of carbocation species. Further mechanistic studies are currently in progress in our laboratory.

## Author contributions

B. X. and Y. L. performed experiments and analyzed data. Y. Y. designed research and wrote the paper. L. X. analyzed part of data. T. X. and X.-Y. Y. revised the paper.

## Conflicts of interest

There are no conflicts to declare.

## Supplementary Material

RA-012-D1RA08015F-s001
