# Supplementary material for: Benzyl thioether formation merging copper catalysis
Source: RSC Adv. 2021 Dec 23;12(2):692–7. doi: 10.1039/d1ra08015f (PMC8697992; doi:10.1039/d1ra08015f)
Supplement: RA-012-D1RA08015F-s001 [file RA-012-D1RA08015F-s001.pdf]

Electronic Supplementary Material (ESI) for RSC Advances.
This journal is © The Royal Society of Chemistry 2021

Supporting Information

Benzyl Thioethers Formation Merging Copper Catalysis

Bing Xu,[a,b,†] Ying Lin,[a,b,†] Yang Ye,*[,a,b] Li Xu,[a,b] Tian Xie*[,a,b] and Xiang-Yang Ye,*[,a,b]

[a]*School of Pharmacy, Hangzhou Normal University, Hangzhou, Zhejiang 311121, PR China*

[b]*Key Laboratory of Elemene Class Anti-Cancer Chinese Medicines; Engineering Laboratory of Development and Application of Traditional Chinese Medicines; Collaborative Innovation Center of Traditional Chinese Medicines of Zhejiang Province, Hangzhou Normal University, Hangzhou, Zhejiang 311121, China*

yangye@hznu.edu.cn; yeyang0711@163.com

Table of Contents

I. Experimental Section S2-S26

Part 1. General Information S2

Part 2. Details of Optimization and Control Experiments S3-S6

Part 3. Coupling of Benzyl Alcohols with Thiols S6-S23

Part 4. The Amination of Benzyl Alcohols with Benzenamine. S24-S25

Part 5. Mechanism Verification S25-S26

II. Spectral Data for New Compounds S27-S96

III. References S97

I. Experimental Section

Part 1. General Information

1. Chemicals and Reagents

All operations were carried out under an atmosphere of air in a sealed flask. DCE (1,2-dichloroethane) and DCM (methylene dichloride) was purchased (Sinopharm Group Co., China) and used directly. Deuterated solvents were used as received ($CDCl_3$ from Maclin Co., China). $Zn(OTf)_2$ (Energy Chemical, China), $Cu(OTf)_2$ (Energy Chemical, China), $Sc(OTf)_2$ (Energy Chemical, China), $Ni(OTf)_2$ (Energy Chemical, China) was purchased and used as received. Unless otherwise noted, all other reagents and starting materials were purchased from commercial sources and used without further purification.

2. Physical Method

Column chromatography was performed using silica gel 300-400 mesh (purchased from Qingdao-Haiyang Co., China) as the solid support. All NMR spectra were recorded on Bruker Avance 500 MHz spectrometer at STP unless otherwise indicated. ^{1}H NMR and ^{13}C NMR chemical shifts are reported in δ units, parts per million (ppm) relative to the chemical shift of residual solvent. Reference peaks for chloroform in ^{1}H NMR and ^{13}C NMR spectra were set at 7.26 ppm and 77.16 ppm, respectively. High resolution mass spectra were measured on Bruker MicroTOF II ESI-TOF mass spectrometer. Low resolution mass spectra were measured on Agilent 1260 Infinity II/6125 mass spectrometer. Melting point was recorded on a micro melting point apparatus (X-4, YUHUA Co., Ltd, Gongyi, China).

Part 2. Details of Optimization and Control Experiments

Typical procedure for optimization reaction conditions: 2-Phenyl-2-Propanol (0.36 mmol), the catalyst were added to a dried test tube equipped with a stir bar under air. The tube was capped with a rubber septum. 4-Fluorothiophenol and a solvent were then added via syringes. After the reaction mixture was allowed to stir overnight under the air atmosphere at 25 ºC, it was directly loaded onto a silica column without work-up. The residue in the reaction vessel was rinsed with small amount of CH_2Cl_2 or eluent. A quick flash column offered a mixture of the product with other impurities.

Table S1. Screening of temperature for the reaction of **1** and **2**

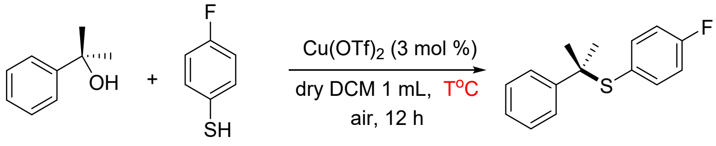

1 (0.36 mmol) **2** (0.30 mmol) **3**

Entry	T°C	yield%[a]
1	0°C	34
2	r.t.(25°C)	96
3	50°C	98
4	80°C	75

[a]Isolated yield.

Table S2. Screening of solvent for the reaction of **1** and **2**

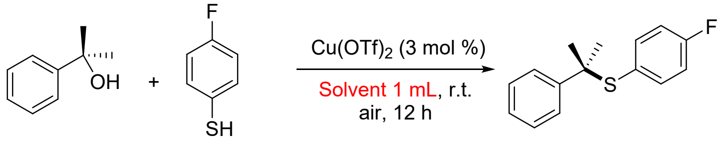

1 (0.36 mmol) **2** (0.30 mmol) **3**

entry	Solvent	yield%[a]
1	dry DCM	96
2	DCE instead of DCM	91

[a]Isolated yield.

Table S3. Change of $Cu(OTf)_2$ loading for the reaction of **1** and **2**

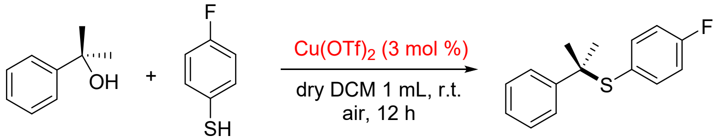

1 (0.36 mmol) **2** (0.30 mmol) **3**

Entry	Catalyst	yield%[a]
1	w/o $Cu(OTf)_2$	ND[b]
2	1 mmol % $Cu(OTf)_2$	65
3	3 mmol % $Cu(OTf)_2$	96
4	5 mmol % $Cu(OTf)_2$	99
5	8 mmol % $Cu(OTf)_2$	99
6	3 mmol % $Ni(OTf)_2$	61
7	3 mmol % $Zn(OTf)_2$	15
8	3 mmol % $Sc(OTf)_2$	44

[a]Isolated yield.; [b]Not detected.

Table S4. Variation of the molar ratios of **1** and **2**

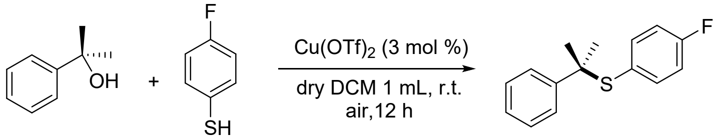

1 (0.36 mmol) **2** (0.30 mmol) **3**

Entry	Variation	yield%[a]
1	none	96
2	**1** (0.30 mmol)	84
3	**2** (1.5 equiv)	99

[a]Isolated yield.

Table S5. Screening of Cu sources for the reaction of **1** and **2**

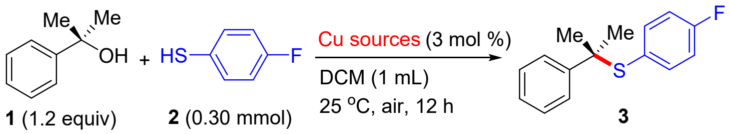

entry	Cu sources	yield%[a]	entry	Cu sources	yield%[a]
1	Cu	ND[b]	16	Copper(II) pyrophosphate	5
2	CuO	ND[b]	17	Copper(II) oxalate	1
3	Cu_2O	ND[b]	18	Copper(II) phthalocyanine	ND[b]
4	CuS	39	19	Copper(I) thiophene-2-carboxylate	ND[b]
5	Cu_2S	87	20	Copper(II) D-gluconate	ND[b]
6	CuF_2	5	21	Copper(I) diphenylphosphinate	ND[b]
7	CuCl	11	22	$Cu(OTs)_2$	36
8	$CuBr_2$	24	23	CuOTf	84
9	CuBr	35	24	$CuSO_4$	20
10	CuBr•DMS	36	25	$CuSO_4$•$5H_2O$	27
11	CuI	5	26	$Cu(acac)_2$	ND[b]
12	$Cu(OAc)_2$	ND[b]	27	$Cu(PPh_3)_3Cl$	ND[b]
13	CuOAc	ND[b]	28	$Cu(Py)_4(OTf)_2$	ND[b]
14	CuSCN	ND[b]	29	$Cu(CH_3CN)_4BF_4$	45
15	$Cu(BF_4)_2$	77	30	$Cu(CH_3CN)_4OTf$	66

[a]isolated yield; [b]Not detected.

Part 3. Coupling of Benzyl Alcohols with Thiols

1. General Procedure

General procedure for the reaction of benzyl alcohols with thiols (method A): To a dried test tube was charged with benzyl alcohols (49.0 mg, 0.36 mmol, 120 mol %), $Cu(OTf)_2$ (3.3 mg, 0.009 mmol, 3 mol %) at r.t.The tube was capped with a rubber septum. Thiols (0.30 mmol, 100 mol %) was added followed by addition of DCM (1 mL) via syringes The reaction mixture was allowed to maintain r.t. and stirred overnight. The reaction mixture was purified by column chromatograph to afford the product as a solid or oil.

2. Experimental Details

(4-Fluorophenyl)(2-phenylpropan-2-yl)sulfane (3).

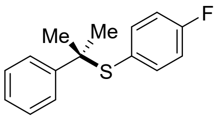

This compound was prepared according to the method A. After purification by column chromatography (SiO_2: petroleum ether), the title compound was isolated in 96% yield (70.9 mg, 0.288 mmol) as a pale yellow oil.

^{1}H NMR (500 MHz, $CDCl_3$): δ 7.41–7.32 (m, 2H), 7.29–7.25 (m, 2H), 7.22–7.17 (m, 1H), 7.12–7.02 (m, 2H), 6.92–6.78 (m, 2H), 1.67 (s, 6H).

^{13}C NMR (126 MHz, $CDCl_3$): δ 146.04, 138.58, 138.51, 127.92, 126.62, 126.55, 115.41, 115.24, 51.03, 29.46.

^{19}F NMR (471 MHz, $CDCl_3$): δ -112.66 (s).

(4-Chlorophenyl)(2-phenylpropan-2-yl)sulfane (4).

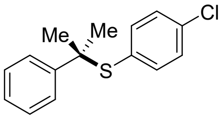

This compound was prepared according to the method A. After purification by column chromatography (SiO_2: petroleum ether), the title compound was isolated in 99% yield (77.9 mg, 0.296 mmol) as a colorless oil.

^{1}H NMR (500 MHz, $CDCl_3$): δ 7.42–7.34 (m, 2H), 7.30–7.24 (m, 2H), 7.23–7.18 (m, 1H), 7.17–7.09 (m, 2H), 7.06–6.96 (m, 2H), 1.68 (s, 6H).

^{13}C NMR (126 MHz, $CDCl_3$): δ 146.00, 137.62, 134.97, 128.44, 127.98, 126.69, 126.57, 51.28, 29.59.

(3-Chlorophenyl)(2-phenylpropan-2-yl)sulfane (5).

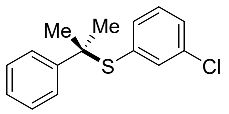

This compound was prepared according to the method A. After purification by column chromatography (SiO_2: petroleum ether), the title compound was isolated in 76% yield (59.9 mg, 0.228 mmol) as a colorless oil.

^{1}H NMR (500 MHz, $CDCl_3$): δ 7.43–7.36 (m, 2H), 7.32–7.27 (m, 2H), 7.25–7.20 (m, 2H), 7.13–7.03 (m, 2H), 6.97 (dt, J = 7.8, 1.3 Hz, 1H), 1.70 (s, 6H).

^{13}C NMR (126 MHz, $CDCl_3$): δ 148.07, 146.55, 129.70, 127.86, 126.80, 126.38, 123.89, 119.08, 110.52, 55.77, 55.54, 29.38.

(2-Chlorophenyl)(2-phenylpropan-2-yl)sulfane (6).

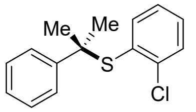

This compound was prepared according to the method A. After purification by column chromatography (SiO_2: petroleum ether), the title compound was isolated in 83% yield (65.4 mg, 0.249 mmol) as a pale yellow oil.

^{1}H NMR (500 MHz, $CDCl_3$): δ 7.46 (dd, J = 8.4, 1.3 Hz, 2H), 7.40 (dd, J = 8.0, 1.4 Hz, 1H), 7.31–7.25 (m, 2H), 7.25–7.21 (m, 1H), 7.17 (ddd, J = 8.0, 7.2, 1.8 Hz, 1H), 7.00–6.91 (m, 2H), 1.74 (s, 6H).

^{13}C NMR (126 MHz, $CDCl_3$): δ 145.97, 137.50, 129.82, 129.42, 128.08, 126.76, 126.52, 126.37, 52.88, 29.77.

(4-Bromophenyl)(2-phenylpropan-2-yl)sulfane (7).

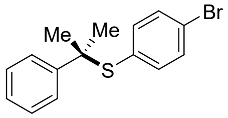

This compound was prepared according to the method A. After purification by column chromatography (SiO_2: 2% ethyl acetate in petroleum ether), the title compound was isolated in 93% yield (85.7 mg, 0.279 mmol) as a colorless oil.

^{1}H NMR (500 MHz, $CDCl_3$): δ 7.39 (d, J = 8.1 Hz, 2H), 7.30–7.24 (m, 4H), 7.20 (td, J = 6.7, 6.1, 1.5 Hz, 1H), 6.94 (dd, J = 8.4, 2.5 Hz, 2H), 1.68 (d, J = 2.6 Hz, 6H).

^{13}C NMR (126 MHz, $CDCl_3$): δ 145.99, 137.83, 132.01, 131.40, 127.99 , 126.70, 126.57, 123.25, 51.27, 29.62 .

4-((2-Phenylpropan-2-yl)thio)benzonitrile (8).

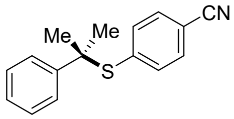

This compound was prepared according to the method A. After purification by column chromatography (SiO_2: 5% ethyl acetate in petroleum ether), the title compound was isolated in 49% yield (37.3 mg, 0.147 mmol) as a yellows oil.

^{1}H NMR (500 MHz, $CDCl_3$): δ 7.41 (dd, J = 16.8, 8.0 Hz, 4H), 7.30 (t, J = 7.5 Hz, 2H), 7.23 (t, J = 7.2 Hz, 1H), 7.11 (d, J = 8.2 Hz, 2H), 1.73 (s, 6H).

^{13}C NMR (126 MHz, $CDCl_3$): δ 145.67, 134.81, 131.66, 128.27, 127.01, 126.51, 118.58, 111.30, 52.31, 30.03.

HRMS (ESI) m/z ($[M+H]^+$) calcd for $C_{16}H_{16}NS$: 254.0998. Found: 254.0959.

M.p.: 124-126 ºC.

(3,4-Dichlorophenyl)(2-phenylpropan-2-yl)sulfane (9).

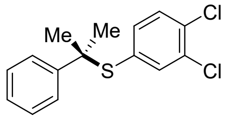

This compound was prepared according to the method A. After purification by column chromatography (SiO_2: petroleum ether), the title compound was isolated in 95% yield (84.7 mg, 0.285 mmol) as a pale yellow oil.

^{1}H NMR (500 MHz, $CDCl_3$): δ 7.43–7.36 (m, 2H), 7.30 (dd, J = 8.4, 6.7 Hz, 2H), 7.25–7.20 (m, 2H), 7.12 (s, 1H), 6.87 (dd, J = 8.3, 2.1 Hz, 1H), 1.70 (s, 6H).

^{13}C NMR (126 MHz, $CDCl_3$): δ 145.61, 137.50, 135.19, 131.97, 129.90, 128.07, 126.91, 126.56, 51.79, 29.56.

(3,5-Bis(trifluoromethyl)phenyl)(2-phenylpropan-2-yl)sulfane (10).

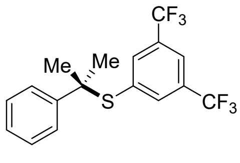

This compound was prepared according to the method A. After purification by column chromatography (SiO_2: petroleum ether), the title compound was isolated in 60% yield (65.6 mg, 0.180 mmol) as a pale yellow oil.

^{1}H NMR (500 MHz, $CDCl_3$): δ 7.70 (s, 1H), 7.38 (s, 2H), 7.34 (dd, J = 8.4, 1.4 Hz, 2H), 7.31–7.26 (m, 2H), 7.26–7.21 (m, 1H), 1.74 (s, 6H).

^{13}C NMR (126 MHz, $CDCl_3$): δ 144.92, 136.35, 135.49, 131.45, 131.19, 128.24, 127.21, 126.49, 123.94, 121.98, 121.95, 121.92, 121.76, 52.48, 29.47.

^{19}F NMR (471 MHz, $CDCl_3$): δ -63.12 (s).

(2-Phenylpropan-2-yl)(p-tolyl)sulfane (11).

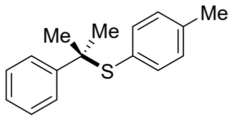

This compound was prepared according to the method A. After purification by column chromatography (SiO_2: petroleum ether), the title compound was isolated in 80% yield (57.9 mg, 0.239 mmol) as a colorless oil.

^{1}H NMR (500 MHz, $CDCl_3$): δ 7.41 (dd, J = 8.4, 1.3 Hz, 2H), 7.27 (t, J = 7.7 Hz, 2H), 7.22–7.17 (m, 1H), 7.05–6.93 (m, 4H), 2.29 (s, 3H), 1.67 (s, 6H).

^{13}C NMR (126 MHz, $CDCl_3$): δ 146.48, 138.66, 136.62, 129.29, 129.07, 127.87, 126.58, 126.47, 50.76, 29.63, 21.22.

HRMS (ESI) m/z ($[M+H]^+$) calcd for $C_{16}H_{19}S$: 243.1202. Found: 243.1198.

(4-(Tert-butyl)phenyl)(2-phenylpropan-2-yl)sulfane (12).

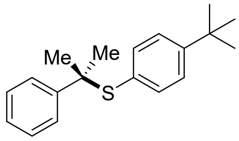

This compound was prepared according to the method A. After purification by column chromatography (SiO_2: petroleum ether), the title compound was isolated in 99% yield (84.5 mg, 0.297 mmol) as a yellow oil.

^{1}H NMR (500 MHz, $CDCl_3$): δ 7.45–7.41 (m, 2H), 7.28 (dd, J = 8.4, 6.8 Hz, 2H), 7.22–7.17 (m, 3H), 7.11–7.06 (m, 2H), 1.68 (s, 6H), 1.27 (s, 9H).

^{13}C NMR (126 MHz, $CDCl_3$): δ 151.73, 136.19, 127.88, 126.54, 126.48, 125.30, 50.82, 34.59, 31.25, 29.73.

HRMS (ESI) m/z ($[M+H]^+$) calcd for $C_{19}H_{25}S$: 285.1671. Found: 285.1686.

(4-Methoxyphenyl)(2-phenylpropan-2-yl)sulfane (13).

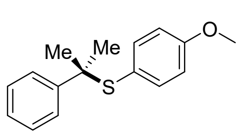

This compound was prepared according to the method A. After purification by column chromatography (SiO_2: petroleum ether), the title compound was isolated in 94% yield (73.2 mg, 0.283 mmol) as a yellow solid.

^{1}H NMR (500 MHz, $CDCl_3$): δ 7.42–7.33 (m, 2H), 7.28–7.24 (m, 2H), 7.23–7.16 (m, 1H), 7.11–7.00 (m, 2H), 6.77–6.64 (m, 2H), 3.76 (s, 3H), 1.66 (s, 6H).

^{13}C NMR (126 MHz, $CDCl_3$): δ 160.19, 146.39, 138.31, 127.84, 126.55, 126.44, 123.66, 113.76, 55.23, 50.73, 29.42.

M.p.: 49-51 ºC.

(3,4-Dimethoxyphenyl)(2-phenylpropan-2-yl)sulfane (14).

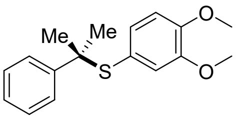

This compound was prepared according to the method A. After purification by column chromatography (SiO_2: 10% ethyl acetate in petroleum ether), the title compound was isolated in 73% yield (63.2 mg, 0.219 mmol) as a yellow oil.

^{1}H NMR (500 MHz, $CDCl_3$): δ 7.41–7.35 (m, 2H), 7.27 (dd, J = 8.4, 6.8 Hz, 2H), 7.22–7.15 (m, 1H), 6.86 (dd, J = 8.2, 2.0 Hz, 1H), 6.71 (d, J = 8.3 Hz, 1H), 6.34 (d, J = 2.0 Hz, 1H), 3.84 (s, 3H), 3.55 (s, 3H), 1.68 (s, 6H).

^{13}C NMR (126 MHz, $CDCl_3$): δ 149.64, 146.55, 129.70, 127.86, 126.80, 126.37, 123.88, 119.08, 110.52, 55.77, 55.54, 50.91, 29.37.

HRMS (ESI) m/z ($[M+H]^+$) calcd for $C_{17}H_{21}O_2S$: 289.1257. Found: 289.1256.

2-((2-Phenylpropan-2-yl)thio)benzoic acid (15).

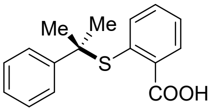

This compound was prepared according to the method A. After purification by column chromatography (SiO_2: 20% ethyl acetate in petroleum ether), the title compound was isolated in 96% yield (78.5 mg, 0.288 mmol) as a white solid.

^{1}H NMR (500 MHz, $CDCl_3$): δ 8.28 (dd, J = 7.9, 1.6 Hz, 1H), 7.44 (t, J = 7.6 Hz, 1H), 7.36 (d, J = 7.7 Hz, 2H), 7.30 (dddd, J = 17.5, 9.0, 4.5, 2.0 Hz, 4H), 7.04 (dd, J = 7.8, 1.3 Hz, 1H), 1.76 (s, 6H).

^{13}C NMR (126 MHz, MeOD): δ 146.48, 136.93, 134.45, 133.56, 129.81, 128.75, 127.84, 126.87, 126.32 , 126.29, 51.39, 29.37.

HRMS (ESI) m/z ($[M+K]^+$) calcd for $C_{16}H_{16}KO_2S$: 311.0503. Found: 311.0494.

M.p.: 112-114 ºC.

4-((2-Phenylpropan-2-yl)thio)phenol (16).

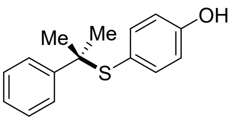

This compound was prepared according to the method A. After purification by column chromatography (SiO_2: 10% ethyl acetate in petroleum ether),

the title compound was isolated in 99% yield (72.6 mg, 0.297 mmol) as a yellow oil.

^{1}H NMR (500 MHz, $CDCl_3$): δ 7.40–7.35 (m, 2H), 7.26 (dd, J = 8.4, 6.8 Hz, 2H), 7.23–7.15 (m, 1H), 7.08–6.95 (m, 2H), 6.69–6.57 (m, 2H), 5.24 (s, 1H), 1.66 (s, 6H).

^{13}C NMR (126 MHz, $CDCl_3$): δ 156.38, 138.56, 127.85, 126.55, 126.47, 115.32, 50.77, 29.38.

HRMS (ESI) m/z ($[M+Na]^+$) calcd for $C_{15}H_{16}NaOS$: 267.0814. Found: 267.0818.

Naphthalen-2-yl(2-phenylpropan-2-yl)sulfane (17).

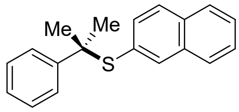

This compound was prepared according to the method A. After purification by column chromatography (SiO_2: petroleum ether), the title compound was isolated in 89% yield (74.4 mg, 0.267 mmol) as a white solid.

^{1}H NMR (500 MHz, $CDCl_3$): δ 7.80–7.72 (m, 1H), 7.70–7.56 (m, 3H), 7.49–7.36 (m, 4H), 7.28 (t, J = 7.4 Hz, 2H), 7.24–7.20 (m, 1H), 7.13 (dd, J = 8.5, 1.7 Hz, 1H), 1.73 (s, 6H).

^{13}C NMR (126 MHz, $CDCl_3$): δ 146.41, 136.18, 133.18, 130.37, 127.96, 127.87, 127.52, 126.65, 126.60, 126.55, 126.11, 51.38, 29.76.

HRMS (ESI) m/z ($[M+H]^+$) calcd for $C_{19}H_{19}S$: 279.1202. Found: 279.0928.

M.p.: 53-55 ºC.

2-((2-Phenylpropan-2-yl)thio)thiophene (18).

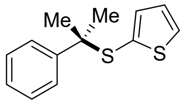

This compound was prepared according to the method A. After purification by column chromatography (SiO_2: petroleum ether), the title compound was isolated in 71% yield (49.9 mg, 0.213 mmol) as a yellow oil.

^{1}H NMR (500 MHz, $CDCl_3$): δ 7.46–7.40 (m, 2H), 7.33–7.26 (m, 3H), 7.24–7.19 (m, 1H), 6.91 (dd, J = 5.4, 3.6 Hz, 1H), 6.85 (dd, J = 3.5, 1.3 Hz, 1H), 1.71 (s, 6H).

^{13}C NMR (126 MHz, $CDCl_3$): δ 145.63, 136.61, 130.80, 127.99, 127.16, 126.76, 126.62, 52.27, 29.29.

Benzyl(2-phenylpropan-2-yl)sulfane (19).

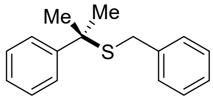

This compound was prepared according to the method A. After purification by column chromatography (SiO_2: petroleum ether), the title compound was isolated in 87% yield (63.3 mg, 0.261 mmol) as a yellow oil.

^{1}H NMR (500 MHz, $CDCl_3$): δ 7.58 (d, J = 7.8 Hz, 2H), 7.35 (t, J = 7.7 Hz, 2H), 7.25 (s, 1H), 7.24–7.19 (m, 2H), 7.17 (d, J = 7.0 Hz, 1H), 7.12 (d, J = 7.1 Hz, 2H), 3.39 (s, 2H), 1.72 (s, 6H).

^{13}C NMR (126 MHz, $CDCl_3$): δ 146.35, 138.21, 128.98, 128.39, 128.20, 126.76, 126.68, 126.59, 48.60, 34.58, 30.29.

HRMS (ESI) m/z ($[M+K]^+$) calcd for $C_{16}H_{18}KS$: 281.0761. Found: 281.0952.

Methyl 3-((2-phenylpropan-2-yl)thio)propanoate (20).

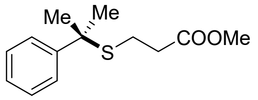

This compound was prepared according to the method A. After purification by column chromatography (SiO_2: 5% ethyl acetate in petroleum ether), the title compound was isolated in 64% yield (46.0 mg, 0.193 mmol) as a yellow oil.

^{1}H NMR (500 MHz, $CDCl_3$): δ 7.60–7.47 (m, 2H), 7.32 (dd, J = 8.5, 7.1 Hz, 2H), 7.21 (td, J = 7.1, 1.2 Hz, 1H), 3.62 (s, 3H), 2.49 (t, J = 7.6 Hz, 2H), 2.32 (t, J = 7.5 Hz, 2H), 1.71 (s, 6H).

^{13}C NMR (126 MHz, $CDCl_3$): δ 146.29, 128.14, 126.57, 126.47, 51.68, 47.85, 34.14, 30.16, 24.47.

HRMS (ESI) m/z ($[M+K]^+$) calcd for $C_{13}H_{18}O_2KS$: 277.0659. Found: 277.0840.

Dodecyl(2-phenylpropan-2-yl)sulfane (21).

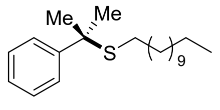

This compound was prepared according to the method A. After purification by column chromatography (SiO_2: petroleum ether), the title compound was isolated in 97% yield (93.1 mg, 0.290 mmol) as a pale yellow oil.

^{1}H NMR (500 MHz, $CDCl_3$): δ 7.61–7.46 (m, 2H), 7.31 (t, J = 7.8 Hz, 2H), 7.19 (t, J = 7.3 Hz, 1H), 2.20 (t, J = 7.4 H $CDCl_3$z, 2H), 1.70 (s, 6H), 1.40–1.33 (m, 2H), 1.31–1.14 (m, 18H), 0.88 (t, J = 6.9 Hz, 3H).

^{13}C NMR (126 MHz, $CDCl_3$): δ 127.99, 126.46, 126.29, 47.34, 31.93, 30.29, 29.65, 29.64, 29.58, 29.46, 29.43, 29.36, 29.18, 29.07, 22.70, 14.13.

HRMS (ESI) m/z ($[M+H]^+$) calcd for $C_{21}H_{37}S$: 321.2610. Found: 321.3147.

sec-Butyl(2-phenylpropan-2-yl)sulfane (22).

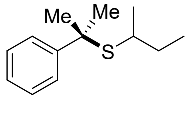

This compound was prepared according to the method A. After purification by column chromatography (SiO_2: petroleum ether), the title compound was

isolated in 83% yield (51.7 mg, 0.248 mmol) as a pale yellow oil.

^{1}H NMR (500 MHz, $CDCl_3$): δ 7.60–7.48 (m, 2H), 7.33–7.27 (m, 2H), 7.22–7.15 (m, 1H), 2.31 (h, J = 6.8 Hz, 1H), 1.72 (d, J = 3.1 Hz, 6H), 1.34 (ddt, J = 14.4, 9.7, 7.2 Hz, 2H), 1.01 (d, J = 6.9 Hz, 3H), 0.78 (t, J = 7.4 Hz, 3H).

^{13}C NMR (126 MHz, $CDCl_3$): δ 147.14, 127.88, 126.60, 126.29, 47.97, 40.64, 31.09, 30.90, 30.81, 22.81, 11.19.

Cyclopentyl(2-phenylpropan-2-yl)sulfane (23).

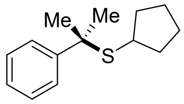

This compound was prepared according to the method A. After purification by column chromatography (SiO_2: petroleum ether), the title compound was isolated in 52% yield (34.4 mg, 0.156 mmol) as a yellow oil.

^{1}H NMR (500 MHz, $CDCl_3$): δ 7.58–7.52 (m, 2H), 7.31 (t, J = 7.8 Hz, 2H), 7.20 (t, J = 7.3 Hz, 1H), 2.66 (p, J = 7.8 Hz, 1H), 1.73 (s, 6H), 1.62–1.52 (m, 3H), 1.44–1.26 (m, 5H).

^{13}C NMR (126 MHz, DMSO-d_6): δ 123.20, 121.76, 121.54, 43.23, 37.65, 30.31, 26.15, 20.06.

HRMS (ESI) m/z ($[M+K]^+$) calcd for $C_{14}H_{20}KS$: 259.0917. Found: 259.1111.

Cyclohexyl(2-phenylpropan-2-yl)sulfane (24).

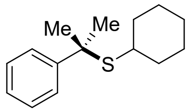

This compound was prepared according to the method A. After purification by column chromatography (SiO_2: petroleum ether), the title compound was isolated in 74% yield (52.2 mg, 0.222 mmol) as a pale yellow oil.

^{1}H NMR (500 MHz, $CDCl_3$): δ 7.55 (dd, J = 8.4, 1.3 Hz, 2H), 7.34–7.26 (m, 2H), 7.22–7.15 (m, 1H), 2.30 (ddd, J = 10.2, 6.5, 3.5 Hz, 1H), 1.71 (s, 6H), 1.60 (tq, J = 13.7, 4.5, 4.0 Hz, 4H), 1.43 (s, 1H), 1.27–1.13 (m, 5H).

^{13}C NMR (126 MHz, $CDCl_3$): δ 127.88, 126.53, 126.27, 48.17, 42.49, 35.32, 30.95, 26.12, 25.55.

HRMS (ESI) m/z ($[M+K]^+$) calcd for $C_{15}H_{22}KS$: 273.1074. Found: 273.1258.

1,4-Bis((2-phenylpropan-2-yl)thio)butane (25).

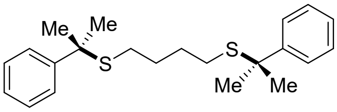

This compound was prepared according to the method A except using 2-phenyl-2-propanol (98.0 mg, 0.72 mmol, 240 mol %). After purification by column chromatography (SiO_2: 5% ethyl acetate in petroleum ether), the title compound was isolated in 99% yield (55.8 mg, 0.156 mmol) as a colorless oil.

^{1}H NMR (500 MHz, $CDCl_3$): δ 7.49 (d, J = 8.0 Hz, 4H), 7.32–7.24 (m, 4H), 7.18 (t, J = 7.3 Hz, 2H), 2.08 (t, J = 6.6 Hz, 4H), 1.66 (s, 12H), 1.32 (dd, J = 9.9, 3.5 Hz, 4H).

^{13}C NMR (126 MHz, $CDCl_3$): δ 146.69, 128.00, 126.44, 126.33, 47.39, 30.26, 28.83, 28.35.

HRMS (ESI) m/z ($[M+Na]^+$) calcd for $C_{22}H_{30}NaS_2$: 381.1681. Found: 381.1636.

1,4-Bis((2-phenylpropan-2-yl)thio)benzene (26).

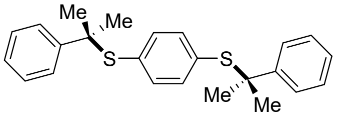

This compound was prepared according to the method A except using 2-phenyl-2-propanol (98.0 mg, 0.72 mmol, 240 mol %). After purification by column chromatography (SiO_2: 5% ethyl acetate in petroleum ether), the title compound was isolated in 85% yield (48.4 mg, 0.128 mmol) as a pale yellow solid.

^{1}H NMR (500 MHz, $CDCl_3$): δ 7.39–7.32 (m, 4H), 7.25 (t, J = 7.6 Hz, 4H), 7.19 (t, J = 7.2 Hz, 2H), 6.89 (s, 4H), 1.65 (s, 12H).

^{13}C NMR (126 MHz, CDCl3): δ 146.13, 135.95, 133.60, 127.92, 126.51, 51.26, 29.69.

M.p.: 137-139 ºC.

(4-Fluorophenyl)(trityl)sulfane (27).

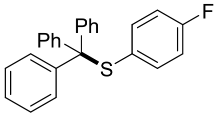

This compound was prepared according to the method A. After purification by column chromatography (SiO_2: petroleum ether), the title compound was isolated in 67% yield (74.5 mg, 0.201 mmol) as a colorless oil.

^{1}H NMR (500 MHz, $CDCl_3$): δ 7.41–7.38 (m, 6H), 7.22 (dd, J = 8.4, 6.4 Hz, 6H), 7.20–7.15 (m, 3H), 6.95–6.89 (m, 2H), 6.71–6.66 (m, 2H).

^{13}C NMR (126 MHz, $CDCl_3$): δ 144.39, 137.55, 137.48, 129.93, 127.71, 126.71, 115.28, 115.11, 71.05.

^{19}F NMR (471 MHz, $CDCl_3$): δ -112.63 (s).

HRMS (ESI) m/z ($[M+Na]^+$) calcd for $C_{25}H_{19}FNaS$: 393.1084. Found: 393.1099.

(1,1-Diphenylethyl)(4-fluorophenyl)sulfane (28).

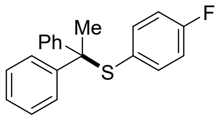

This compound was prepared according to the method A. After purification by column chromatography (SiO_2: petroleum ether), the title compound was isolated in 69% yield (63.8 mg, 0.207 mmol) as a colorless oil.

^{1}H NMR (500 MHz, $CDCl_3$): δ 7.41–7.33 (m, 4H), 7.27 (t, J = 7.4 Hz, 4H), 7.24–7.19 (m, 2H), 7.02–6.95 (m, 2H), 6.84–6.75 (m, 2H), 1.90 (s, 3H).

^{13}C NMR (126 MHz, DMSO-d_6): δ 141.31, 134.07, 134.00, 123.57, 123.12, 121.96, 110.63, 110.46, 55.02, 25.54.

^{19}F NMR (471 MHz, $CDCl_3$): δ -112.22 (s).

(4-Fluorophenyl)(2-phenylbut-3-yn-2-yl)sulfane (29).

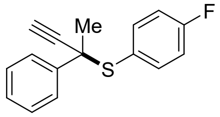

This compound was prepared according to the method A. After purification by column chromatography (SiO_2: petroleum ether), the title compound was isolated in 83% yield (63.8 mg, 0.249 mmol) as a yellow oil.

^{1}H NMR (500 MHz, $CDCl_3$): δ 7.57–7.49 (m, 2H), 7.31–7.28 (m, 2H), 7.27 (s, 1H), 7.27–7.21 (m, 2H), 6.95–6.87 (m, 2H), 2.71 (s, 1H), 1.94 (s, 3H).

^{13}C NMR (126 MHz, $CDCl_3$): δ 141.64, 138.69, 138.62, 128.07, 127.53, 126.70, 115.49, 115.32, 85.94, 74.90, 29.68.

^{19}F NMR (471 MHz, $CDCl_3$): δ -111.65 (s).

HRMS (ESI) m/z ($[M+H]^+$) calcd for $C_{16}H_{14}FS$: 257.0795. Found: 257.0776.

(4-Fluorophenyl)(1-phenylethyl)sulfane (30).

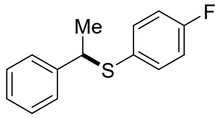

This compound was prepared according to the method A except using $Cu(OTf)_2$ (8.7 mg, 0.024 mmol, 8 mol %), DCE (1 mL) and running at 40°C. After purification by column chromatography (SiO_2: 5% ethyl acetate in petroleum ether), the title compound was isolated in 64% yield (44.6 mg, 0.192 mmol, 0% *ee*) as a colorless oil.

^{1}H NMR (500 MHz, $CDCl_3$): δ 7.28–7.19 (m, 7H), 6.97–6.82 (m, 2H), 4.22 (q, J = 7.0 Hz, 1H), 1.61 (d, J = 7.0 Hz, 3H).

^{13}C NMR (126 MHz, $CDCl_3$): δ 143.01, 135.74, 135.67, 128.36, 127.30, 127.18, 115.80, 115.63, 48.90, 21.92.

^{19}F NMR (471 MHz, $CDCl_3$): δ -113.92 (s).

HRMS (ESI) m/z ($[M+H]^+$) calcd for $C_{14}H_{14}FS$: 271.0354. Found: 271.0561.

HPLC analysis: CHIRALCEL OD-H column, 0.5% *i*PrOH in hexane, 0.5 mL/min, 254 nm UV detector,t_R (minor) = 9.0 min, t_R (major) = 8.7 min.

(4-Fluorophenyl)(1-(4-fluorophenyl)ethyl)sulfane (31).

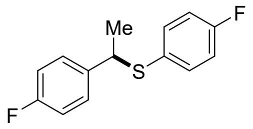

This compound was prepared according to the method A except using $Cu(OTf)_2$ (8.7 mg, 0.024 mmol, 8 mol %), DCE (1 mL) and running at 40°C. After purification by column chromatography (SiO_2: petroleum ether), the title compound was isolated in 60% yield (45.1 mg, 0.180 mmol) as a pale yellow oil.

^{1}H NMR (500 MHz, $CDCl_3$): δ 7.25–7.19 (m, 2H), 7.19–7.13 (m, 2H), 6.97–6.87 (m, 4H), 4.20 (q, J = 7.0 Hz, 1H), 1.59 (d, J = 7.0 Hz, 3H).

^{13}C NMR (126 MHz, $CDCl_3$): δ 135.92, 135.86, 128.83, 128.77, 115.88, 115.71, 115.23, 115.06, 48.21, 29.71, 21.97.

^{19}F NMR (471 MHz, $CDCl_3$): δ -113.59 (s), -115.31 (s).

HRMS (ESI) m/z ($[M+K]^+$) calcd for $C_{14}H_{12}F_2KS$: 289.0259. Found: 289.0455.

4-((1-(p-tolyl)ethyl)thio)phenol (32).

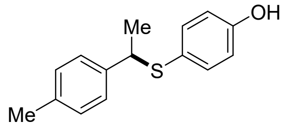

This compound was prepared according to the method A except using $Cu(OTf)_2$ (8.7 mg, 0.024 mmol, 8 mol %), DCE (1 mL) and running at 40°C. After purification by column chromatography (SiO_2: 5% ethyl acetate in petroleum ether), the title compound was isolated in 96% yield (70.4 mg, 0.288 mmol) as a white solid.

^{1}H NMR (500 MHz, $CDCl_3$): δ 7.20–7.16 (m, 2H), 7.13–7.10 (m, 2H), 7.06 (d, J = 7.9 Hz, 2H), 6.71–6.64 (m, 2H), 5.01 (s, OH), 4.14 (q, J = 7.0 Hz, 1H), 2.31 (s, 3H), 1.56 (d, J = 7.0 Hz, 3H).

^{13}C NMR (126 MHz, DMSO-d_6): δ 150.84, 135.49, 131.90, 131.39, 124.23, 122.45, 120.73, 110.96, 44.12, 17.15, 16.35.

HRMS (ESI) m/z ($[M+Na]^+$) calcd for $C_{15}H_{16}NaOS$: 267.0814. Found: 267.0330.

M.p.: 94-96 ºC.

(4-Fluorophenyl)(1-(4-methoxyphenyl)ethyl)sulfane (33).

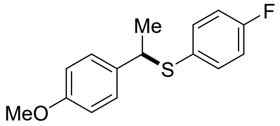

This compound was prepared according to the method A except using $Cu(OTf)_2$ (8.7 mg, 0.024 mmol, 8 mol %), DCE (1 mL) and running at 40°C. After purification by column chromatography (SiO_2: petroleum ether), the title compound was isolated in 88% yield (69.3 mg, 0.264 mmol) as a yellow oil.

^{1}H NMR (500 MHz, $CDCl_3$): δ 7.25–7.20 (m, 2H), 7.19–7.12 (m, 2H), 6.93–6.87 (m, 2H), 6.84–6.76 (m, 2H), 4.2–4.16 (m, 1H), 3.78 (s, 3H).

^{13}C NMR (126 MHz, $CDCl_3$): δ 158.65, 135.72, 135.65, 135.03, 128.35, 115.78, 115.61, 113.70, 55.26, 48.31, 22.05.

^{19}F NMR (471 MHz, $CDCl_3$): δ -114.07 (s).

HRMS (ESI) m/z ($[M+NH_4]^+$) calcd for $C_{15}H_{19}FNOS$: 280.1166. Found: 280.0336.

Benzhydryl(4-fluorophenyl)sulfane (34).

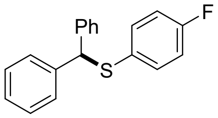

This compound was prepared according to the method A except using $Cu(OTf)_2$ (8.7 mg, 0.024 mmol, 8 mol %), DCE (1 mL) and running at 40°C. After purification by column chromatography (SiO_2: petroleum ether), the title compound was isolated in 53% yield (46.8 mg, 0.159 mmol) as a pale yellow oil.

^{1}H NMR (500 MHz, $CDCl_3$): δ 7.38 (dd, J = 8.5, 1.1 Hz, 4H), 7.28 (dd, J = 8.3, 6.7 Hz, 4H), 7.25–7.17 (m, 4H), 6.91–6.81 (m, 2H), 5.41 (s, 1H).

^{13}C NMR (126 MHz, DMSO-d_6): δ 136.08, 129.39, 129.33, 123.79, 123.64, 122.57, 111.16, 110.98, 53.97.

^{19}F NMR (471 MHz, $CDCl_3$): δ -114.34 (s).

HRMS (ESI) m/z ($[M+K]^+$) calcd for $C_{19}H_{15}FKS$: 333.0510. Found: 333.0745.

(4-Fluorophenyl)(1,2,3,4-tetrahydronaphthalen-1-yl)sulfane (35).

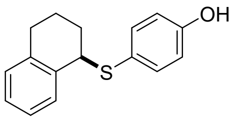

This compound was prepared according to the method A except using $Cu(OTf)_2$ (8.7 mg, 0.024 mmol, 8 mol %), DCE (1 mL) and running at 40°C. After purification by column chromatography (SiO_2: 5% ethyl acetate in petroleum ether), the title compound was isolated in 90% yield (69.2 mg, 0.270 mmol) as a yellow oil.

^{1}H NMR (500 MHz, $CDCl_3$): δ 7.46–7.42 (m, 2H), 7.35 (dd, J = 7.0, 2.1 Hz, 1H), 7.13 (ddd, J =

7.2, 4.7, 1.9 Hz, 2H), 7.10–7.06 (m, 1H), 7.03–6.98 (m, 2H), 4.44 (t, J = 4.1 Hz, 1H), 2.86–2.69 (m, 2H), 2.27–2.15 (m, 1H), 1.96 (dtd, J = 10.6, 9.3, 8.8, 5.5 Hz, 2H), 1.75 (tdd, J = 9.6, 5.1, 2.4 Hz, 1H).

^{13}C NMR (126 MHz CDCl$_3$): δ 137.58, 135.39, 135.23, 135.17, 130.45, 129.31, 127.15, 125.70, 116.11, 115.94, 48.75, 29.10, 28.40, 18.57.

HRMS (ESI) m/z ([M+K]$^+$) calcd for $C_{16}H_{16}KOS$: 295.0553. Found: 295.0573.

3-((4-Fluorophenyl)thio)butan-2-one (36).

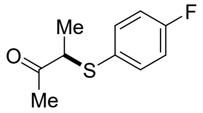

This compound was prepared according to the method A except using Cu(OTf)$_2$ (8.7 mg, 0.024 mmol, 8 mol %), DCE (1 mL) and running at 40°C. After purification by column chromatography (SiO$_2$: 5% ethyl acetate in petroleum ether), the title compound was isolated in 64% yield (35.0 mg, 0.136 mmol) as a pale yellow oil.

^{1}H NMR (500 MHz, CDCl$_3$): δ 7.40–7.34 (m, 2H), 7.03–6.98 (m, 2H), 3.67 (q, J = 7.0 Hz, 1H), 2.29 (s, 3H), 1.37 (d, J = 7.0 Hz, 3H).

^{13}C NMR (126 MHz, CDCl$_3$): δ 205.08, 135.96, 135.89, 116.34, 116.17, 52.42, 29.71, 15.87.

^{19}F NMR (471 MHz, CDCl$_3$): δ -112.45 (s).

HRMS (ESI) m/z ([M+H]$^+$) calcd for $C_{10}H_{12}FOS$: 199.0587. Found: 199.0577.

Cyclohexyl(1-(*p*-tolyl)ethyl)sulfane (37).

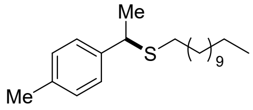

This compound was prepared according to the method A except using Cu(OTf)$_2$ (8.7 mg, 0.024 mmol, 8 mol %), DCE (1 mL) and running at 40°C. After purification by column chromatography (SiO$_2$: petroleum ether), the title compound was isolated in 63% yield (60.6 mg, 0.189 mmol) as a colorless oil.

^{1}H NMR (500 MHz, CDCl$_3$): δ 7.22 (d, J = 8.0 Hz, 2H), 7.11 (d, J = 7.9 Hz, 2H), 3.91 (q, J = 7.0 Hz, 1H), 2.33 (s, 3H), 2.32–2.23 (m, 2H), 1.54 (d, J = 7.0 Hz, 3H), 1.48 (dq, J = 14.8, 7.3, 6.6 Hz, 2H), 1.25 (d, J = 2.4 Hz, 18H), 0.88 (t, J = 6.9 Hz, 3H).

^{13}C NMR (126 MHz, CDCl$_3$): δ 141.21, 136.52, 129.10, 127.11, 43.73, 31.94, 31.31, 29.67, 29.65, 29.61, 29.50, 29.40, 29.37, 29.22, 28.96, 22.71, 21.08, 14.14.

Cyclohexyl(1-(*p*-tolyl)ethyl)sulfane (38).

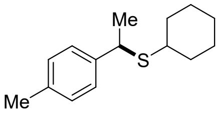

This compound was prepared according to the method A except using $Cu(OTf)_2$ (8.7 mg, 0.024 mmol, 8 mol %), DCE (1 mL) and running at 40°C. After purification by column chromatography (SiO_2: petroleum ether), the title compound was isolated in 59% yield (41.5 mg, 0.177 mmol) as a colorless oil.

^{1}H NMR (500 MHz, $CDCl_3$): δ 7.23 (d, J = 8.0 Hz, 2H), 7.11 (d, J = 7.9 Hz, 2H), 4.01 (q, J = 7.0 Hz, 1H), 2.39 (tt, J = 10.5, 3.6 Hz, 1H), 2.33 (s, 3H), 1.96 (d, J = 12.9 Hz, 1H), 1.73 (t, J = 11.2 Hz, 2H), 1.66 (dd, J = 10.0, 3.9 Hz, 1H), 1.53 (d, J = 7.0 Hz, 3H), 1.35–1.12 (m, 6H).

^{13}C NMR (126 MHz, $CDCl_3$): δ 141.67, 136.42, 129.12, 127.00, 42.70, 42.12, 33.88, 33.30, 26.01, 25.89, 25.85, 23.20, 21.10.

Benzyl(4-fluorophenyl)sulfane (39).

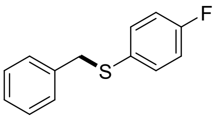

This compound was prepared according to the method A except using $Cu(OTf)_2$ (8.7 mg, 0.024 mmol, 8 mol %), DCE (1 mL) and running at 80°C. After purification by column chromatography (SiO_2: petroleum ether), the title compound was isolated in 76% yield (49.7 mg, 0.228 mmol) as a colorless oil.

^{1}H NMR (500 MHz, $CDCl_3$): δ 7.29–7.19 (m, 7H), 6.98–6.89 (m, 2H), 4.03 (s, 2H).

^{13}C NMR (126 MHz, $CDCl_3$): δ 137.52, 133.46, 133.40, 128.85, 128.46, 127.20, 115.99, 115.82, 40.46.

^{19}F NMR (471 MHz, $CDCl_3$): δ -114.84 (s).

HRMS (ESI) m/z ($[M+NH_4]^+$) calcd for $C_{13}H_{15}FNS$: 236.0904. Found: 236.0833.

([1,1'-Biphenyl]-4-ylmethyl)(4-fluorophenyl)sulfane (40).

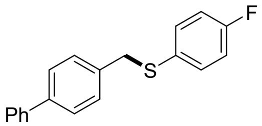

This compound was prepared according to the method A except using $Cu(OTf)_2$ (8.7 mg, 0.024 mmol, 8 mol %), DCE (1 mL) and running at 80°C. After purification by column chromatography (SiO_2: petroleum ether), the title compound was isolated in 74% yield (65.4 mg, 0.222 mmol) as a white solid.

^{1}H NMR (500 MHz, $CDCl_3$): δ 7.57 (dd, J = 8.3, 1.3 Hz, 2H), 7.52–7.48 (m, 2H), 7.42 (t, J = 7.7 Hz, 2H), 7.35 (t, J = 1.3 Hz, 0H), 7.33–7.24 (m, 5H), 7.00–6.89 (m, 2H), 4.07 (s, 2H).

^{13}C NMR (126 MHz, $CDCl_3$): δ 140.67, 140.10, 136.60, 133.50, 133.44, 129.27, 128.79, 127.33, 127.18, 127.02, 116.05, 115.88, 40.20.

^{19}F NMR (471 MHz, $CDCl_3$): δ -114.75 (s).

M.p.: 157-159 ºC.

(4-Fluorophenyl)(4-(trifluoromethyl)benzyl)sulfane (41).

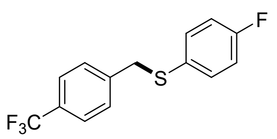

This compound was prepared according to the method A except using $Cu(OTf)_2$ (8.7 mg, 0.024 mmol, 8 mol %), DCE (1 mL) and running at 80°C. After purification by column chromatography (SiO_2: petroleum ether), the title compound was isolated in 57% yield (48.9 mg, 0.171 mmol) as a colorless oil.

^{1}H NMR (500 MHz, $CDCl_3$): δ 7.52 (d, J = 8.1 Hz, 2H), 7.27 (dd, J = 16.8, 7.6 Hz, 4H), 7.00–6.91 (m, 2H), 4.04 (s, 2H).

^{13}C NMR (126 MHz, $CDCl_3$): δ 163.36, 161.39, 141.87, 133.98, 133.92, 129.11, 125.45, 125.42, 125.39, 125.36, 116.20, 116.02, 40.15.

^{19}F NMR (471 MHz, $CDCl_3$): δ -62.47 (s), -113.97 (s).

HRMS (ESI) m/z ($[M+K]^+$) calcd for $C_{14}H_{10}F_4KS$: 325.0071. Found: 325.0271.

(4-Fluorophenyl)(2-methylbenzyl)sulfane (42).

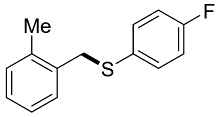

This compound was prepared according to the method A except using $Cu(OTf)_2$ (8.7 mg, 0.024 mmol, 8 mol %), DCE (1 mL) and running at 80°C. After purification by column chromatography (SiO_2: petroleum ether), the title compound was isolated in 65% yield (45.3 mg, 0.195 mmol) as a yellow oil.

^{1}H NMR (500 MHz, $CDCl_3$): δ 7.30–7.26 (m, 2H), 7.15 (d, J = 4.0 Hz, 2H), 7.06 (dd, J = 8.3, 3.3 Hz, 1H), 7.01 (d, J = 7.4 Hz, 1H), 6.98–6.92 (m, 2H), 4.02 (s, 2H), 2.36 (s, 3H).

^{13}C NMR (126 MHz, $CDCl_3$): δ 163.25, 136.72, 133.97, 133.91, 131.06, 130.58, 129.90, 127.61, 126.00, 116.03, 115.86, 38.78, 19.22.

^{19}F NMR (471 MHz, $CDCl_3$): δ -114.60 (s).

HRMS (ESI) m/z ($[M+K]^+$) calcd for $C_{14}H_{13}FKS$: 271.0354. Found: 271.0641.

(4-Fluorophenyl)(3-methylbenzyl)sulfane (43).

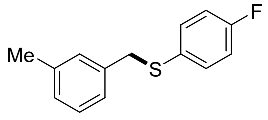

This compound was prepared according to the method A except using

$Cu(OTf)_2$ (8.7 mg, 0.024 mmol, 8 mol %), DCE (1 mL) and running at 80°C. After purification by column chromatography (SiO_2: petroleum ether), the title compound was isolated in 99% yield (69.0 mg, 0.297 mmol) as a yellow oil.

^{1}H NMR (500 MHz, $CDCl_3$): δ 7.28–7.24 (m, 2H), 7.15 (t, J = 7.8 Hz, 1H), 7.06–7.02 (m, 2H), 7.02–6.99 (m, 1H), 6.96–6.92 (m, 2H), 4.00 (s, 2H), 2.30 (s, 3H).

^{13}C NMR (126 MHz, $CDCl_3$): δ 138.14, 137.32, 133.27, 133.20, 129.60, 128.35, 127.98, 125.88, 115.96, 115.79, 40.40, 21.34.

^{19}F NMR (471 MHz, $CDCl_3$): δ -115.00 (s).

HRMS (ESI) m/z ($[M+K]^+$) calcd for $C_{14}H_{13}FKS$: 271.0354. Found: 271.0570.

(4-Fluorophenyl)(4-methylbenzyl)sulfane (44).

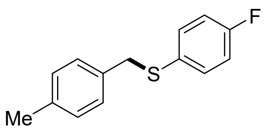

This compound was prepared according to the method A except using $Cu(OTf)_2$ (8.7 mg, 0.024 mmol, 8 mol %), DCE (1 mL) and running at 80°C. After purification by column chromatography (SiO_2: petroleum ether), the title compound was isolated in 67% yield (23.0 mg, 0.105 mmol) as a white solid.

^{1}H NMR (500 MHz, $CDCl_3$): δ 7.29–7.24 (m, 2H), 7.09 (q, J = 8.0 Hz, 4H), 6.94 (t, J = 8.7 Hz, 2H), 4.00 (s, 2H), 2.31 (s, 3H).

^{13}C NMR (126 MHz, $CDCl_3$): δ 136.87, 134.38, 133.21, 133.15, 129.17, 128.72, 115.97, 115.79, 40.11, 21.12.

^{19}F NMR (471 MHz, $CDCl_3$): δ -115.07 (s).

HRMS (ESI) m/z ($[M+K]^+$) calcd for $C_{14}H_{13}FKS$: 271.0354. Found: 271.0556.

M.p.: 52-54 ºC.

(3,5-Bis(trifluoromethyl)benzyl)(4-fluorophenyl)sulfane (45).

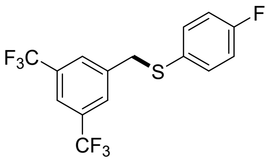

This compound was prepared according to the method A except using $Cu(OTf)_2$ (8.7 mg, 0.024 mmol, 8 mol %), DCE (1 mL) and running at 80°C. After purification by column chromatography (SiO_2: petroleum ether), the title compound was isolated in 59% yield (62.7 mg, 0.177 mmol) as a yellow oil.

^{1}H NMR (500 MHz, $CDCl_3$): δ 7.73 (s, 1H), 7.56 (s, 2H), 7.25 (dd, J = 7.5, 4.6 Hz, 2H), 6.97 (t, J = 8.4 Hz, 2H), 4.07 (s, 2H).

^{13}C NMR (126 MHz, $CDCl_3$): δ 163.77, 161.79, 140.47, 134.97, 131.83, 131.57, 128.91, 124.24, 121.13, 121.10, 121.07, 121.04, 121.01, 116.41, 116.23.

^{19}F NMR (471 MHz, $CDCl_3$): δ -63.02 (s), -112.90 (s).

(4-Fluorophenyl)(naphthalen-1-ylmethyl)sulfane (46).

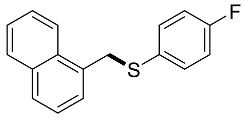

This compound was prepared according to the method A except using $Cu(OTf)_2$ (8.7 mg, 0.024 mmol, 8 mol %), DCE (1 mL) and running at 80°C. After purification by column chromatography (SiO_2: petroleum ether), the title compound was isolated in 66% yield (53.1 mg, 0.198 mmol) as a white solid.

^{1}H NMR (500 MHz, $CDCl_3$): δ 8.11 (d, J = 8.3 Hz, 1H), 7.86 (dd, J = 8.1, 1.3 Hz, 1H), 7.75 (d, J = 8.2 Hz, 1H), 7.52 (dddd, J = 23.4, 8.0, 6.8, 1.4 Hz, 2H), 7.32–7.28 (m, 1H), 7.27–7.25 (m, 1H), 7.24 (s, 1H), 7.17 (dd, J = 6.9, 1.1 Hz, 1H), 6.97–6.87 (m, 2H), 4.47 (s, 2H).

^{13}C NMR (126 MHz, $CDCl_3$): δ 133.99, 133.93, 132.85, 131.34, 128.84, 128.31, 127.39, 126.23, 125.86, 125.15, 123.88, 116.00, 115.82, 38.59.

^{19}F NMR (471 MHz, $CDCl_3$): δ -114.57 (s).

HRMS (ESI) m/z ($[M+K]^+$) calcd for $C_{17}H_{13}FKS$: 307.0354. Found: 307.0556.

M.p.: 72-74 ºC.

2-(((4-Fluorophenyl)thio)methyl)thiophene (47).

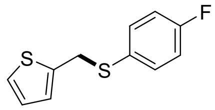

This compound was prepared according to the method A except using $Cu(OTf)_2$ (8.7 mg, 0.024 mmol, 8 mol %), DCE (1 mL) and running at 80°C. After purification by column chromatography (SiO_2: petroleum ether), the title compound was isolated in 40% yield (26.9 mg, 0.120 mmol) as a yellow oil.

^{1}H NMR (500 MHz, $CDCl_3$): δ 7.34–7.30 (m, 2H), 7.17 (d, J = 5.1 Hz, 1H), 6.99–6.94 (m, 2H), 6.88–6.84 (m, 1H), 6.78 (d, J = 3.0 Hz, 1H), 4.23 (s, 2H).

^{13}C NMR (126 MHz $CDCl_3$): δ 163.33, 140.82, 133.92, 133.86, 130.23, 126.69, 126.35, 125.05, 116.09, 115.92, 35.07.

^{19}F NMR (471 MHz, $CDCl_3$): δ -114.26 (s).

HRMS (ESI) m/z ($[M+K]^+$) calcd for $C_{11}H_9FKS_2$: 262.9761. Found: 262.9978.

Butyl(3-methylbenzyl)sulfane (48).

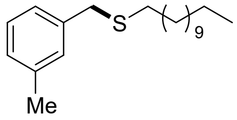

This compound was prepared according to the method A except using $Cu(OTf)_2$ (8.7 mg, 0.024 mmol, 8 mol %), DCE (1 mL) and running at 80°C. After purification by column chromatography (SiO_2: petroleum ether), the title compound was isolated in 48% yield (44.1 mg, 0.144 mmol) as a colorless oil.

^{1}H NMR (500 MHz, $CDCl_3$): δ 7.19 (t, J = 7.5 Hz, 1H), 7.13 (s, 1H), 7.09 (d, J = 7.5 Hz, 1H), 7.04 (d, J = 7.5 Hz, 1H), 3.66 (s, 2H), 2.43–2.39 (m, 2H), 2.34 (s, 3H), 1.58–1.52 (m, 3H), 1.37 (s, 2H), 1.25 (s, 16H), 0.88 (t, J = 6.9 Hz, 4H).

^{13}C NMR (126 MHz $CDCl_3$): δ 138.57, 138.10, 129.56, 128.30, 127.64, 125.88, 36.28, 31.94, 31.48, 29.68, 29.66, 29.62, 29.54, 29.37, 29.27, 29.26, 28.92, 22.71, 21.39, 14.14.

Cyclohexyl(3-methylbenzyl)sulfane (49).

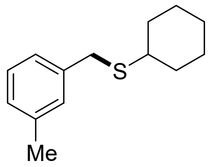

This compound was prepared according to the method A except using $Cu(OTf)_2$ (8.7 mg, 0.024 mmol, 8 mol %), DCE (1 mL) and running at 80°C. After purification by column chromatography (SiO_2: petroleum ether), the title compound was isolated in 42% yield (27.8 mg, 0.126 mmol) as a colorless oil.

^{1}H NMR (500 MHz, $CDCl_3$): δ 7.18 (t, J = 7.5 Hz, 1H), 7.14 (s, 1H), 7.11 (d, J = 7.5 Hz, 1H), 7.04 (d, J = 7.5 Hz, 1H), 3.71 (s, 2H), 2.58 (tt, J = 10.6, 3.7 Hz, 1H), 2.33 (s, 3H), 1.98–1.93 (m, 2H), 1.78–1.71 (m, 2H), 1.62–1.56 (m, 1H), 1.36 (d, J = 9.0 Hz, 1H), 1.35–1.32 (m, 1H), 1.29 (d, J = 6.7 Hz, 1H), 1.27 (d, J = 3.0 Hz, 1H), 1.25–1.22 (m, 1H).

^{13}C NMR (126 MHz $CDCl_3$): δ 138.80, 138.10, 129.51, 128.31, 127.57, 125.80, 43.01, 34.56, 33.40, 26.01, 25.90, 21.40.

Part 4. The Amination of Benzyl Alcohols with Benzenamine

1. General procedure

General procedure for the reaction of benzenamine with benzyl alcohol (method B): To a dried test tube was charged with benzyl alcohol (0.18 mmol, 100 mol %), $Cu(OTf)_2$ (8.7 mg, 0.024 mmol, 8 mol %) at r.t.The tube was capped with a rubber septum. Benzenamine (0.30 mmol, 167 mol %) was added followed by addition of DCE (1 mL) via syringes The reaction mixture was allowed to warm to 70 ºC and stirred overnight. The reaction mixture was purified by column chromatograph to afford the product as a solid or oil.

2. Experimental details

N-(2-phenylpropan-2-yl)aniline (50).

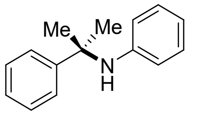

This compound was prepared according to the method B. After purification by column chromatography (SiO_2: 5% ethyl acetate in petroleum ether), the title compound was isolated in 33% yield (12.55 mg, 0.0594 mmol) as a yellow oil.

^{1}H NMR (500 MHz, $CDCl_3$): δ 7.45 (d, J = 8.2 Hz, 2H), 7.30 (t, J = 7.7 Hz, 2H), 7.19 (t, J = 7.3 Hz, 1H), 6.90–6.85 (m, 2H), 6.41 (t, J = 7.3 Hz, 1H), 6.29 (d, J = 7.7 Hz, 2H), 5.88 (s, 1H), 1.56 (s, 6H).

^{13}C NMR (126 MHz, $CDCl_3$): δ 148.47, 147.29, 126.45, 125.82, 115.85, 115.03, 55.42, 30.60.

HRMS (ESI) m/z ($[M+H]^+$) calcd for $C_{15}H_{18}N$: 212.1434. Found: 212.1452.

4-fluoro-N-(1-phenylethyl)aniline (51).

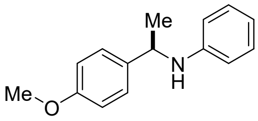

This compound was prepared according to the method B. After purification by column chromatography (SiO_2: 5% ethyl acetate in petroleum ether), the title compound was isolated in 47% yield (30.4 mg, 0.141 mmol) as a yellow oil.

^{1}H NMR (500 MHz, $CDCl_3$): δ 7.27 (d, J = 8.6 Hz, 2H), 7.14–7.03 (m, 2H), 6.85 (d, J = 8.7 Hz, 2H), 6.63 (t, J = 7.3 Hz, 1H), 6.51 (d, J = 7.7 Hz, 2H), 4.44 (q, J = 6.7 Hz, 1H), 1.48 (d, J = 6.7 Hz, 3H).

^{13}C NMR (126 MHz $CDCl_3$): δ 158.30, 148.47, 138.42, 129.07, 127.38, 115.91, 114.15, 113.26, 55.41, 51.86, 25.27.

HRMS (ESI) m/z ([M+H]$^+$) calcd for $C_{15}H_{17}NO$: 228.1383. Found: 228.1361.

Part 5. Mechanism Verification

(4-Fluorophenyl)(1-phenylethyl)sulfane (30).

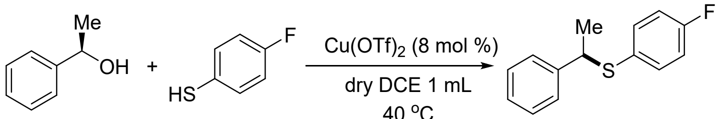

1a: 99% *ee* **2** **30**: 64%, 0% *ee*

This compound was prepared according to the method A except using $Cu(OTf)_2$ (8.7 mg, 0.024 mmol, 8 mol %), DCE (1 mL) and running at 40°C. After purification by column chromatography (SiO_2: 5% ethyl acetate in petroleum ether), the title compound was isolated in 64% yield (44.6 mg, 0.192 mmol, 0% *ee*) as a colorless oil.

^{1}H NMR (500 MHz, $CDCl_3$): δ 7.28–7.19 (m, 7H), 6.97–6.82 (m, 2H), 4.22 (q, J = 7.0 Hz, 1H), 1.61 (d, J = 7.0 Hz, 3H).

^{13}C NMR (126 MHz, $CDCl_3$): δ 143.01, 135.74, 135.67, 128.36, 127.30, 127.18, 115.80, 115.63, 48.90, 21.92.

HRMS (ESI) m/z ([M+H]$^+$) calcd for $C_{14}H_{14}FS$: 271.0354. Found: 271.0561.

HPLC analysis: CHIRALCEL OD-H column, 0.5% *i*PrOH in hexane, 0.5 mL/min, 254 nm UV detector,t_R (minor) = 9.0 min, t_R (major) = 8.7 min.

(Cyclopropyl(phenyl)methyl)(4-fluorophenyl)sulfane (53).

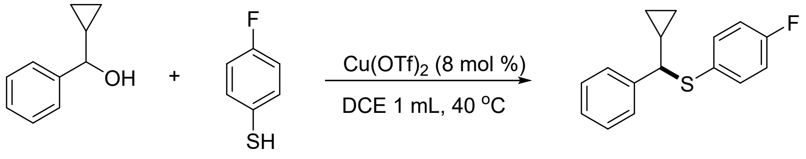

1a **2** **53**: 79 %

The title compound was prepared according the method A except using $Cu(OTf)_2$ (8.7 mg, 0.024 mmol, 8 mol %), DCE (1 mL) and running at 40°C. After purification by a flash column chromatography (SiO_2: petroleum ether), the title compound was isolated in 79% yield (61.2 mg, 0.237 mmol) as a pale yellow oil.

^{1}H NMR (500 MHz, $CDCl_3$): δ 7.28–7.17 (m, 7H), 6.86 (td, J = 8.7, 2.0 Hz, 2H), 3.38 (d, J = 9.7 Hz, 1H), 1.36–1.28 (m, 1H), 0.69 (dd, J = 7.7, 3.2 Hz, 1H), 0.54 (dd, J = 9.1, 4.4 Hz, 1H), 0.33 (dd, J = 9.3, 4.6 Hz, 1H), 0.23 (dd, J = 9.8, 5.1 Hz, 1H).

^{13}C NMR (126 MHz, $CDCl_3$): δ 163.55, 161.58, 141.99, 136.18, 136.11, 128.24, 127.88, 127.12, 115.66, 115.49, 59.97, 16.78, 6.61, 5.18.

^{19}F NMR (471 MHz, $CDCl_3$): δ -113.97 (s).

HRMS (ESI) m/z ($[M+H]^+$) calcd for $C_{16}H_{16}FS$: 297.0510. Found: 297.0720.

Control experiments.

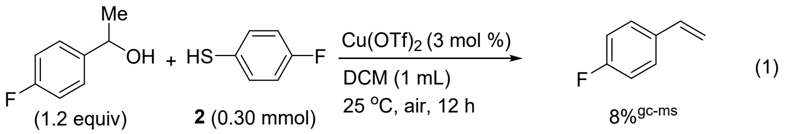

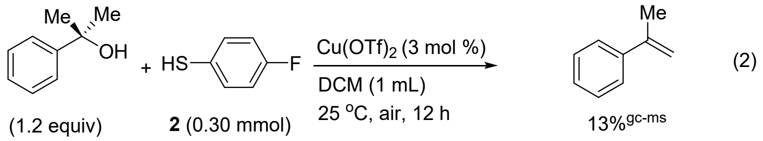

During the reaction of 1-(4-fluorophenyl)ethanol with thiol **2**, we observed the formation of 4-fluorostyrene in ~8 % yield detected by GC-MS (eq 1). Similar elimination product can also be found in reaction of tertiary benzyl alcohol (2-phenyl-2-propanol) with thiol **2** (eq 2). These findings may suggest the occurrence of an E1 reaction, which would compete with an SN1 reaction. The formation of styrene supported a possible elimination of carbocation intermediate, which can be promoted by $Cu(OTf)_2$ in DCM (or DCE) at 25°C or elevated temperatures. Moreover, 2-phenyl-3-butyn-2-ol, 1-phenylethanol, 1-(4-methylphenyl)ethanol, 1-(4-methoxyphenyl)ethanol, 1,2,3,4-tetrahydro-1-naphthol and so on were subjected to the reaction conditions in the presence of thiol **2** to check for a possible E1 reaction. As the result, no elimination was observed. Moreover, in the presence of radical inhibitors such as BHT, the reactions were not inhibited, which exhibited that long-lived radical intermediates might not be involved in the reaction processes. All these above suggested that the SN1 reaction was the most possible mechanism taking place under these conditions.

II. Spectral Data for New Compounds

1. NMR Data for New Compounds

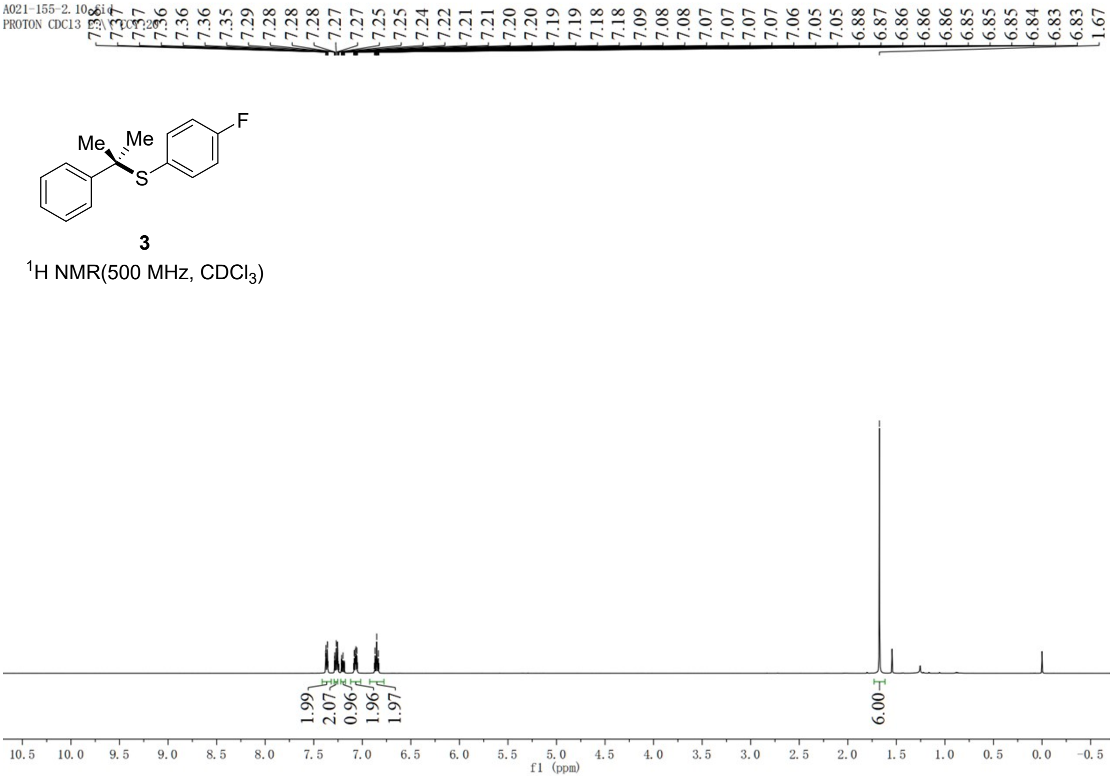

3

^1H NMR(500 MHz, $CDCl_3$)

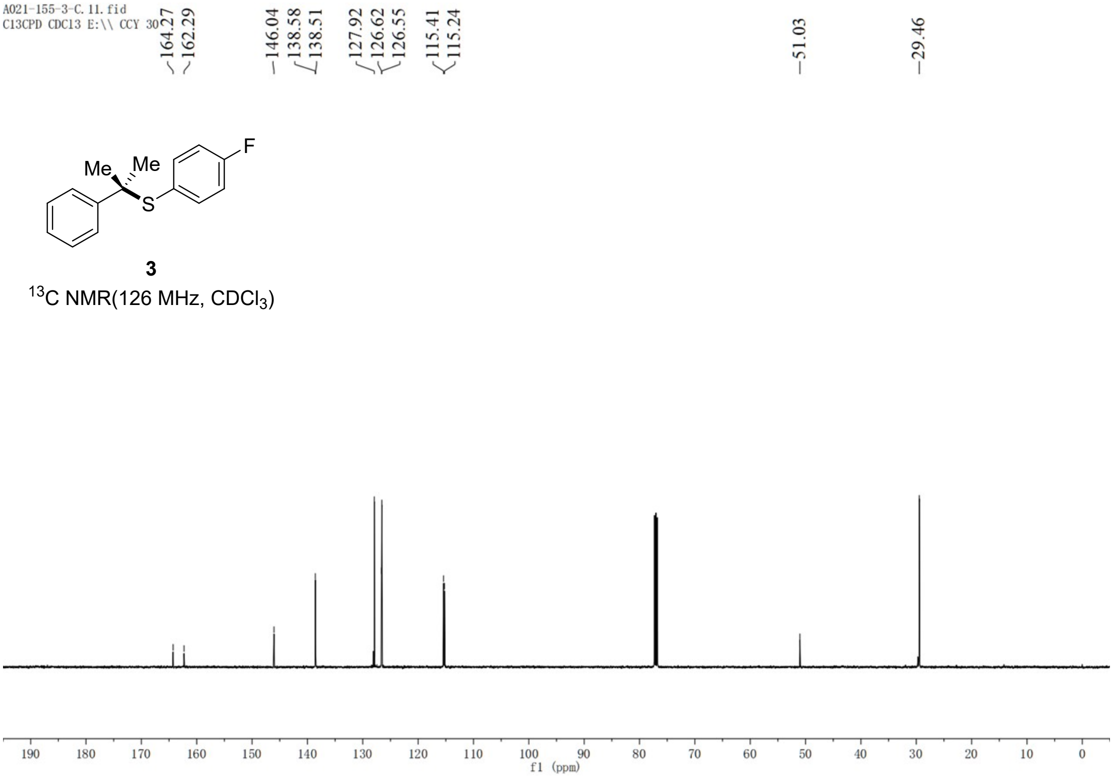

3

^{13}C NMR(126 MHz, $CDCl_3$)

A017-3.11.fid
F19CPD CDCl3 E:\\ CCY 16

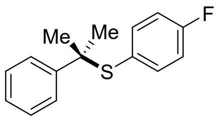

3

^{19}F NMR(471 MHz, CDCl3)

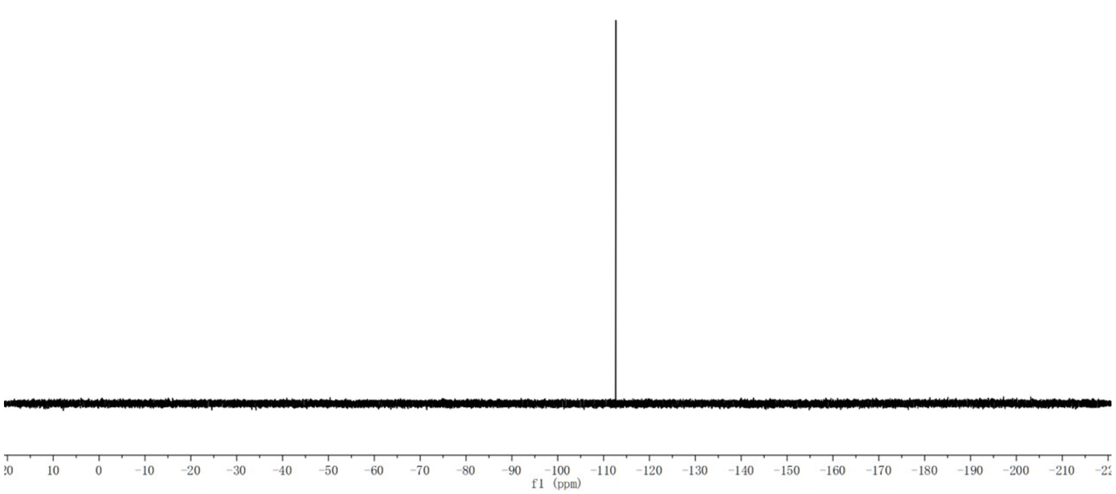

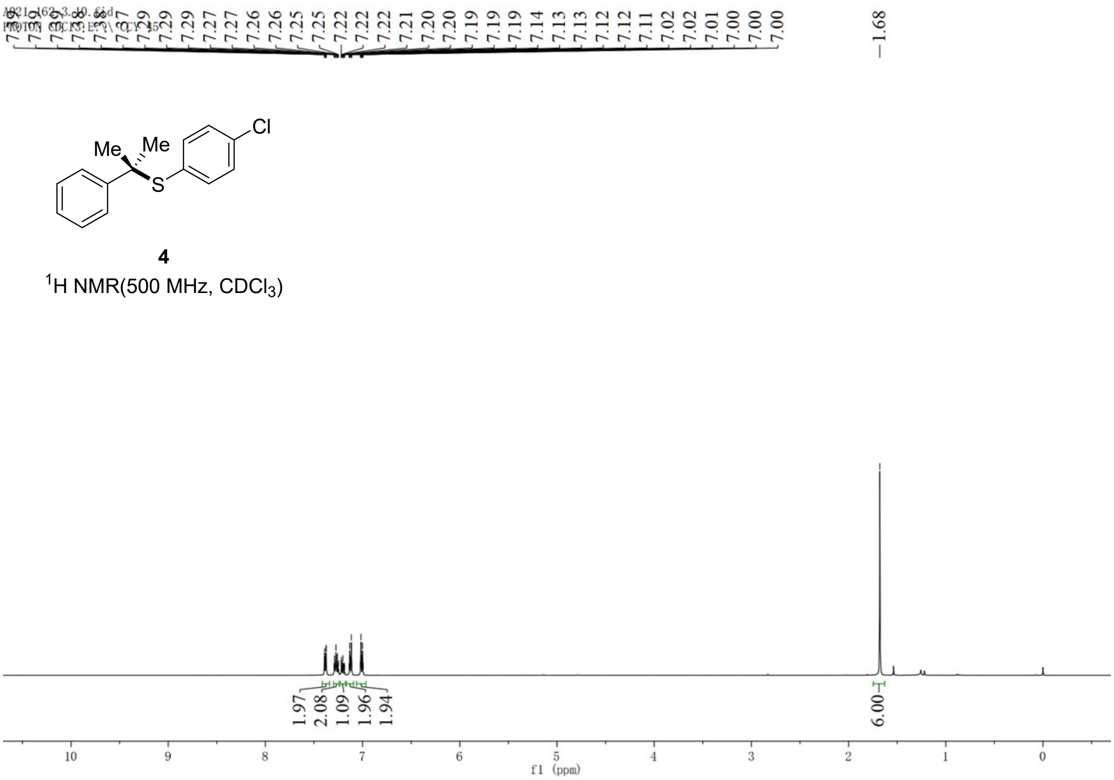

4

^{1}H NMR(500 MHz, $CDCl_3$)

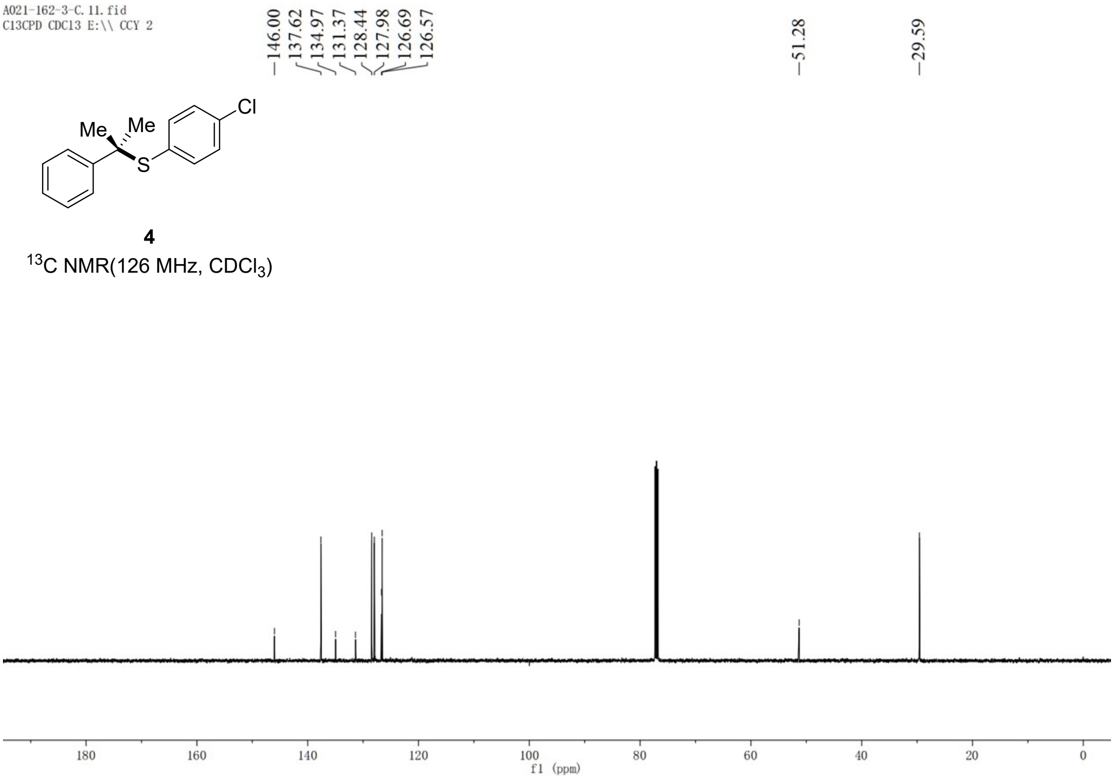

4

^{13}C NMR(126 MHz, $CDCl_3$)

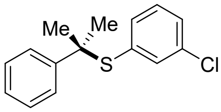

5

^{1}H NMR(500 MHz, $CDCl_3$)

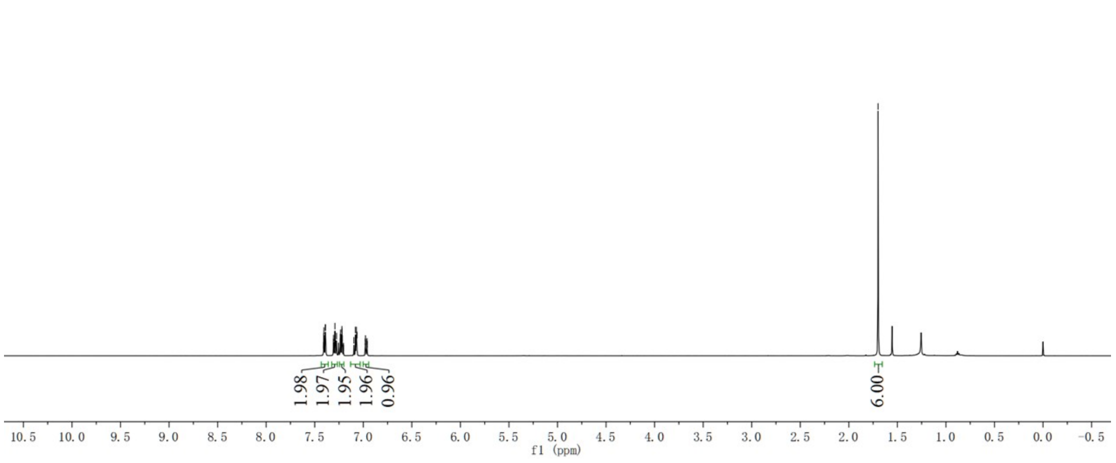

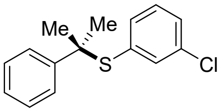

5

^{13}C NMR(126 MHz, $CDCl_3$)

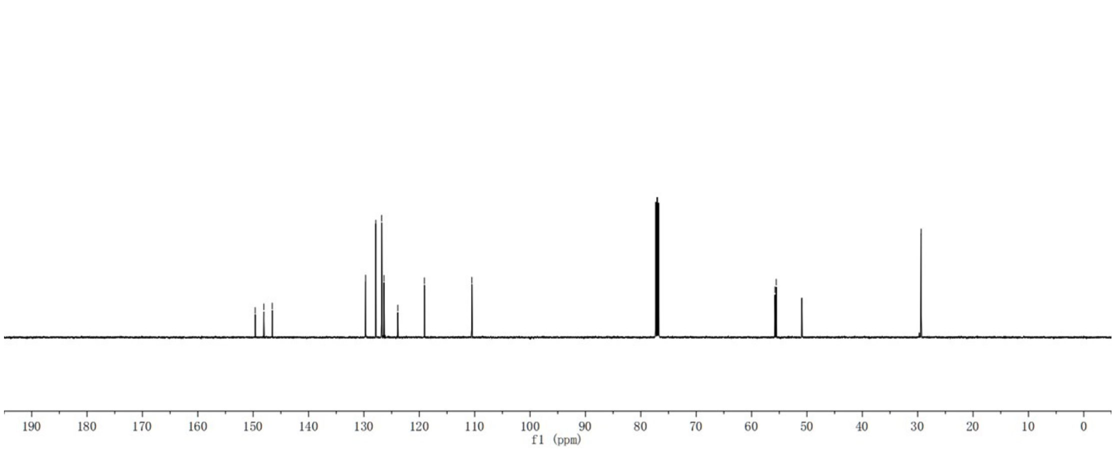

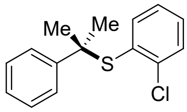

6

^{1}H NMR(500 MHz, $CDCl_3$)

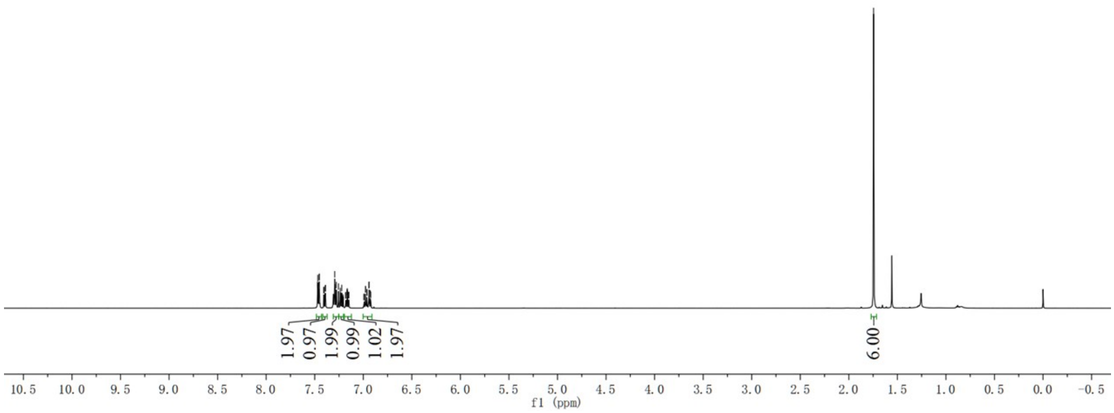

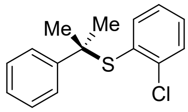

6

^{13}C NMR(126 MHz, $CDCl_3$)

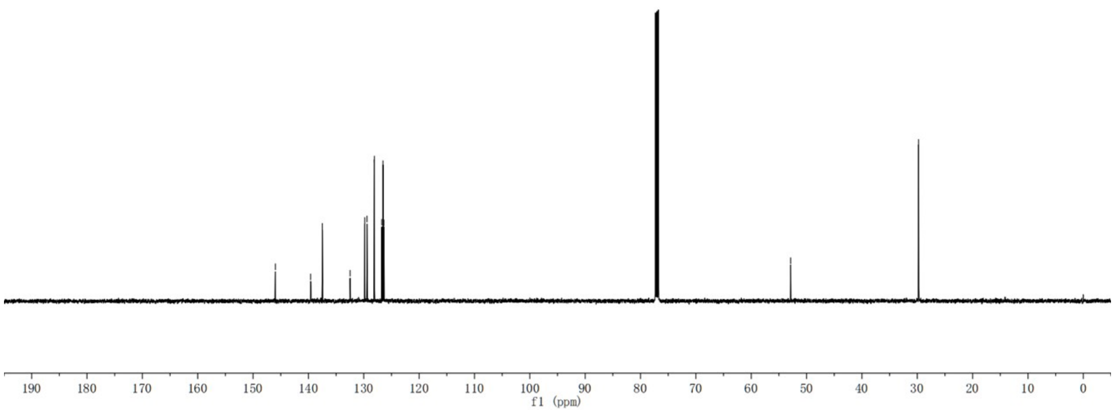

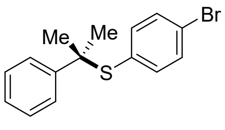

7

^{1}H NMR(500 MHz, $CDCl_3$)

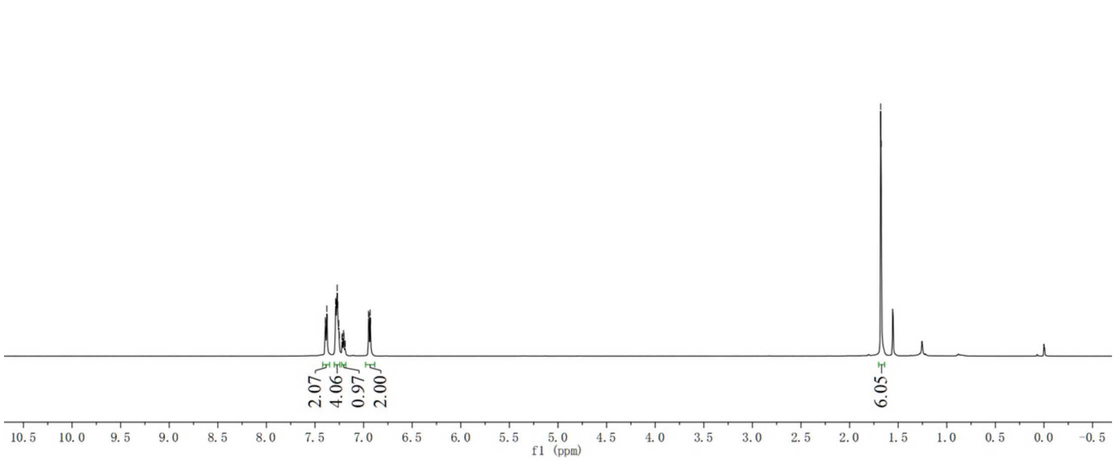

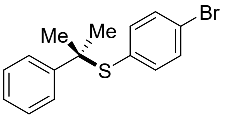

7

^{13}C NMR(126 MHz, $CDCl_3$)

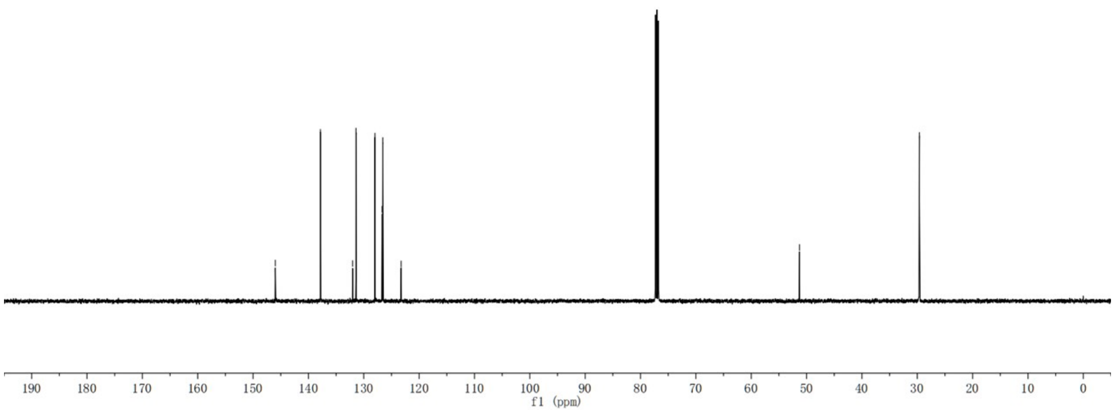

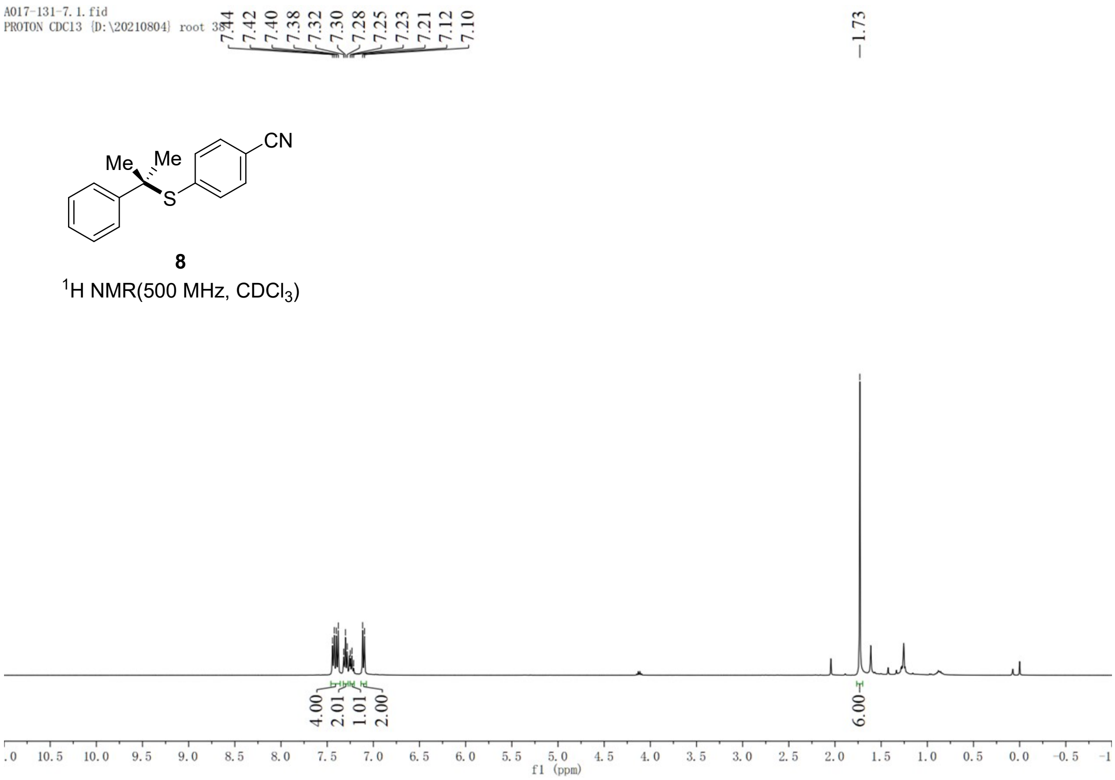
A017-131-7.1.fid
PROTON CDCl3 {D:\20210804} root 38
7.44
7.42
7.40
7.38
7.32
7.30
7.28
7.25
7.23
7.21
7.12
7.10
1.73
Me
Me
S
CN
8
^{1}H NMR(500 MHz, CDCl$_3$)
4.00
2.01
1.01
2.00
6.00
f1 (ppm)

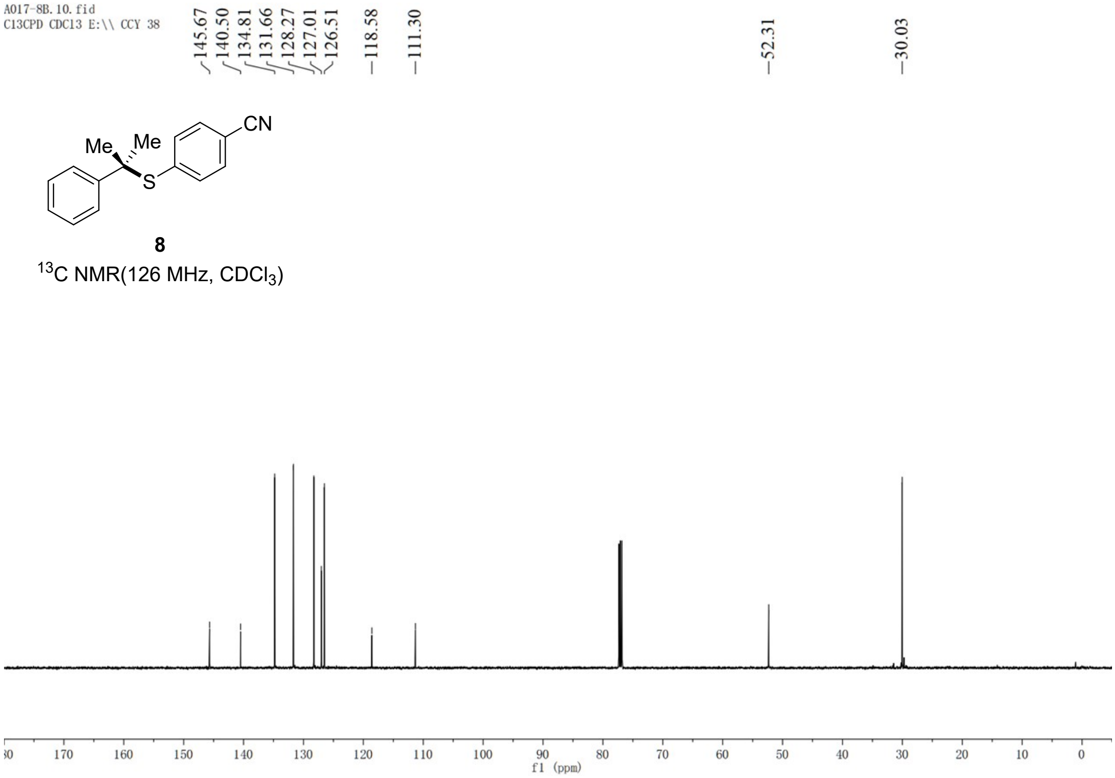
A017-8B.10.fid
C13CPD CDCl3 E:\\ CCY 38
145.67
140.50
134.81
131.66
128.27
127.01
126.51
118.58
111.30
52.31
30.03
Me
Me
S
CN
8
^{13}C NMR(126 MHz, CDCl$_3$)
f1 (ppm)

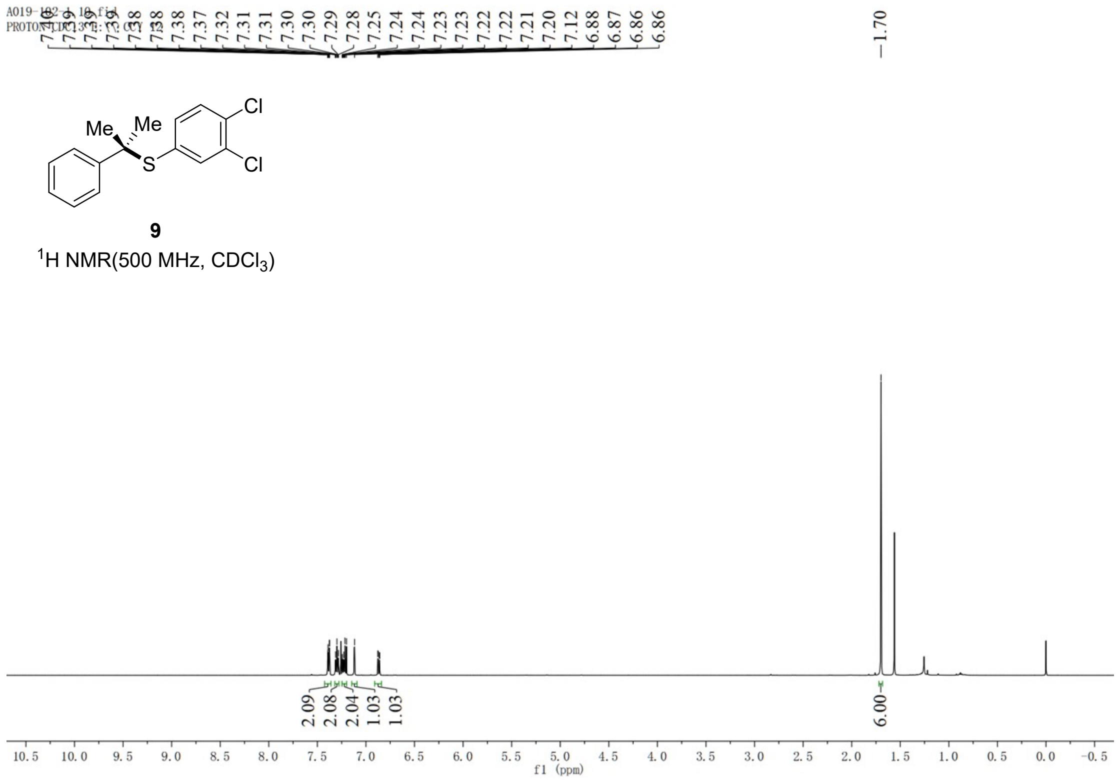

9

^{1}H NMR(500 MHz, $CDCl_3$)

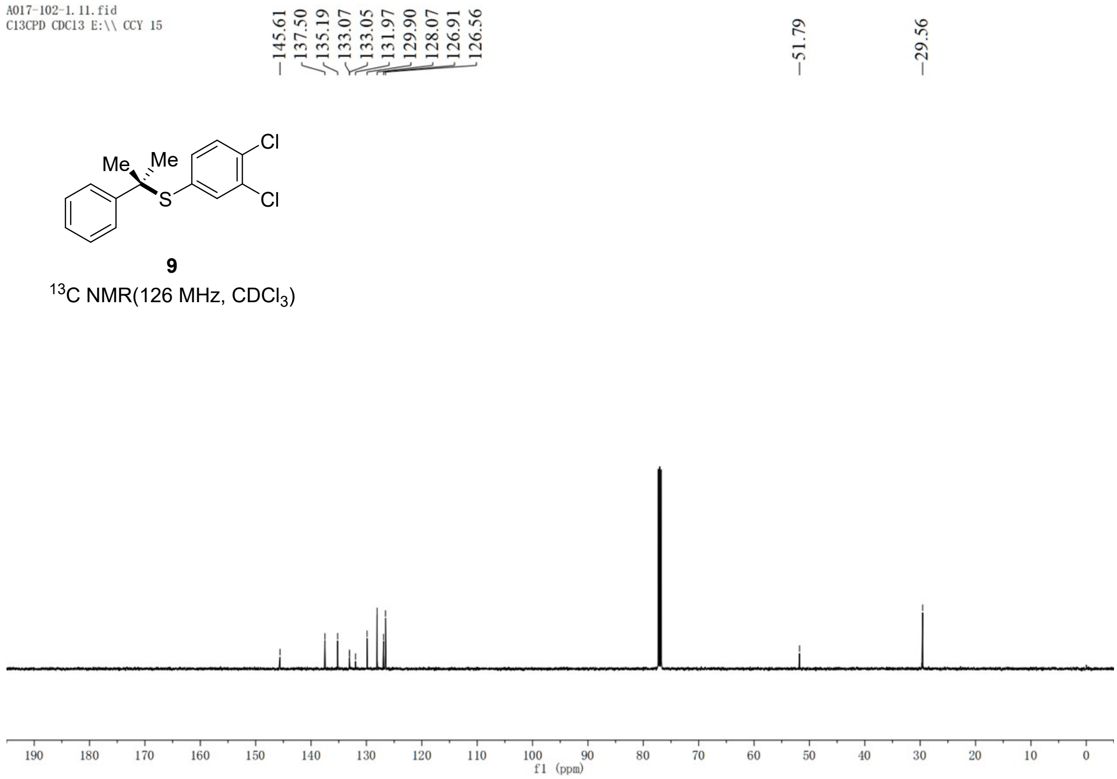

9

^{13}C NMR(126 MHz, $CDCl_3$)

A017-103-4.10.fid
PROTON CDCl3 E:\\ CCY 15

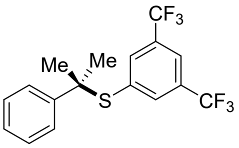

10

^{1}H NMR(500 MHz, $CDCl_3$)

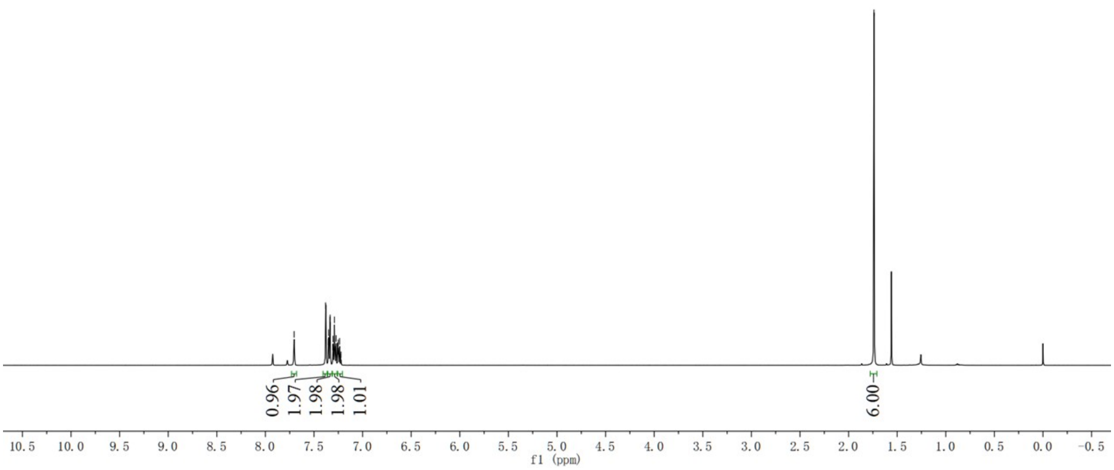

A017-103-4.11.fid
C13CPD CDCl3 E:\\ CCY 8

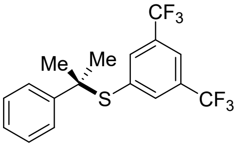

10

^{13}C NMR(126 MHz, $CDCl_3$)

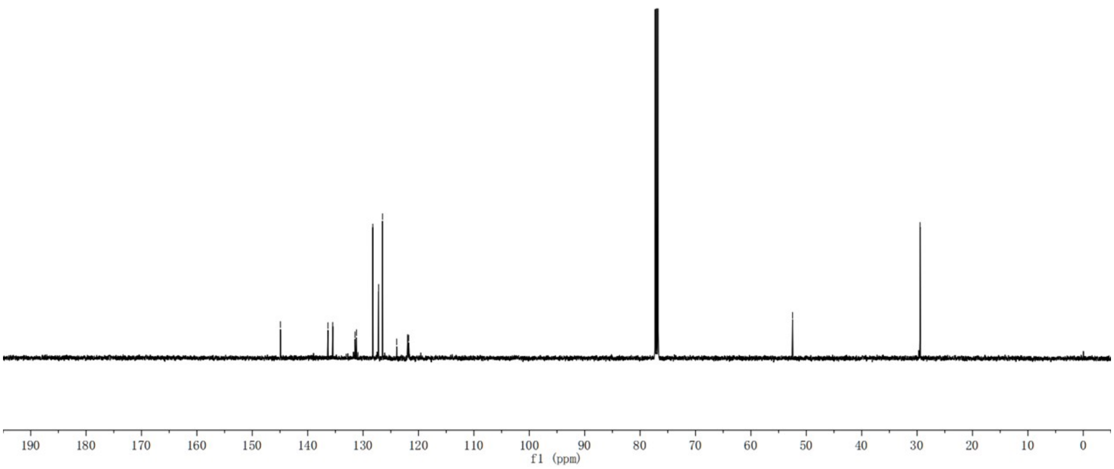

A017-10.11.fid
F19CPD CDCl3 E:\\ CCY 6

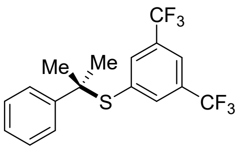

10

^{19}F NMR(471 MHz, $CDCl_3$)

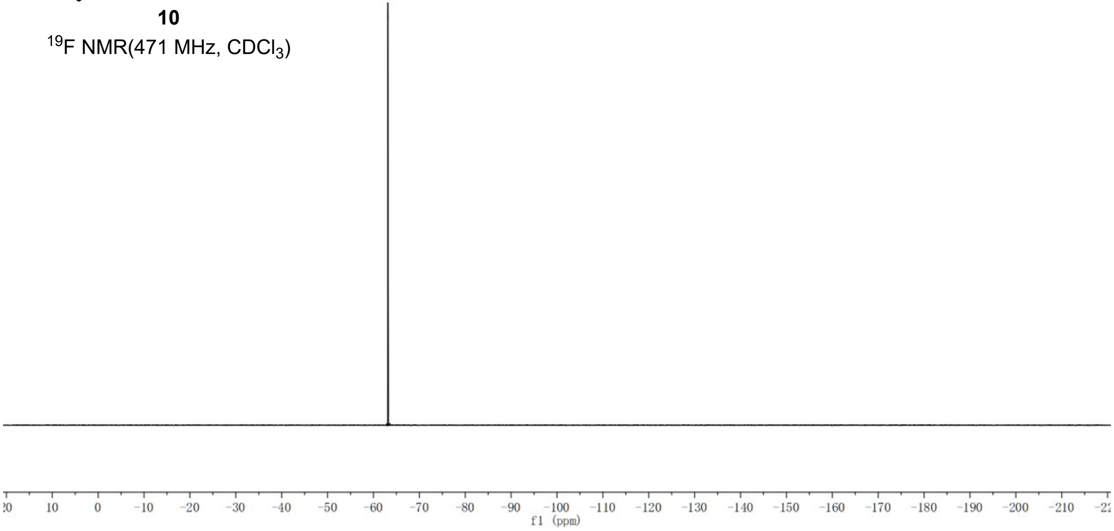

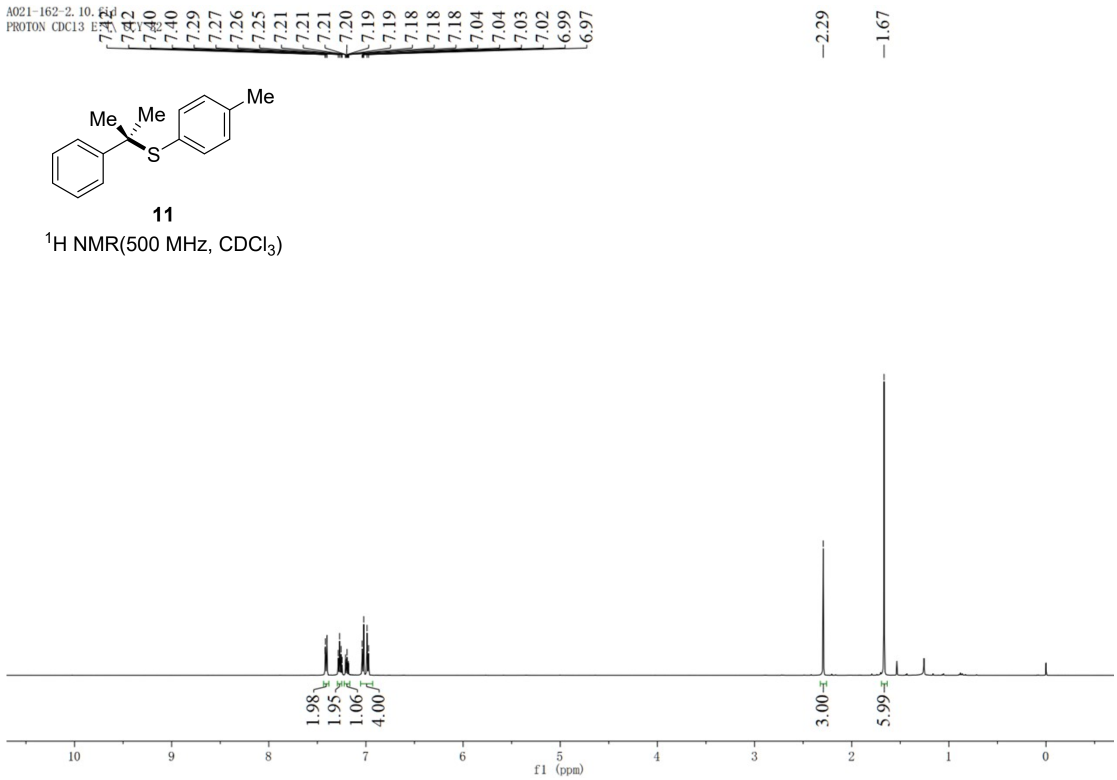

11

^1H NMR(500 MHz, $CDCl_3$)

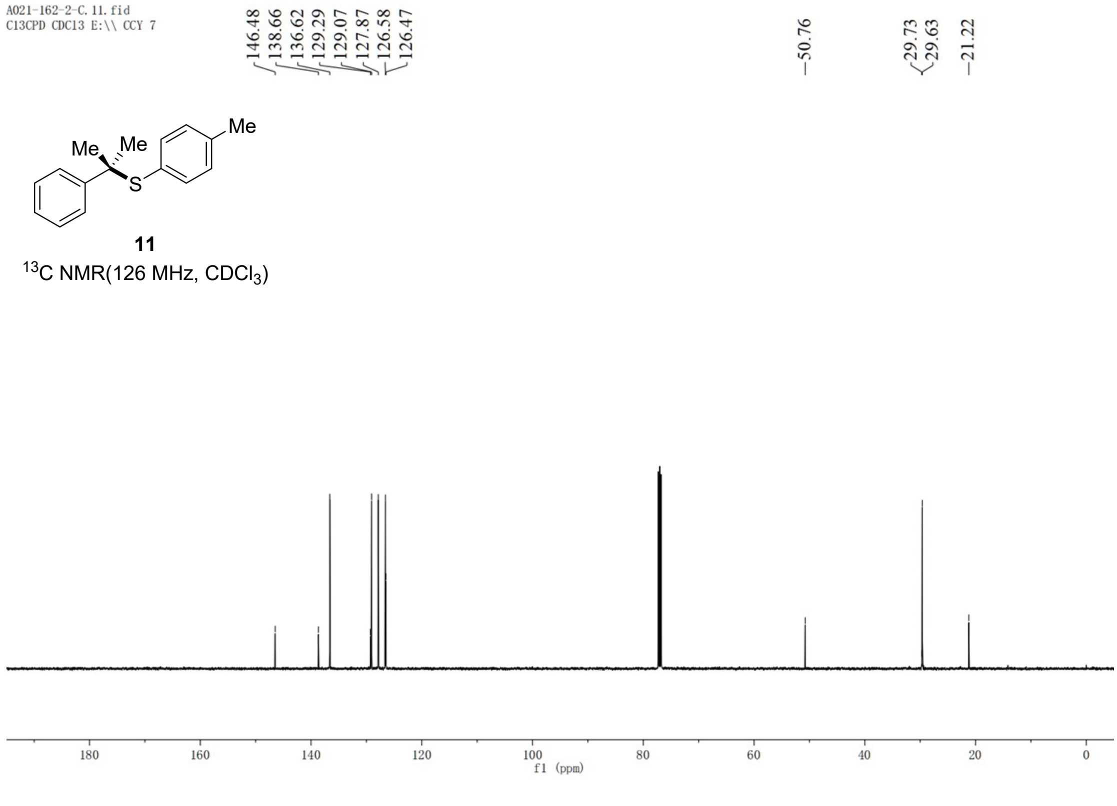

11

^{13}C NMR(126 MHz, $CDCl_3$)

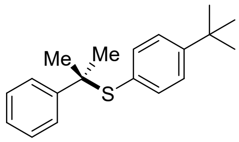

12

^{1}H NMR(500 MHz, $CDCl_3$)

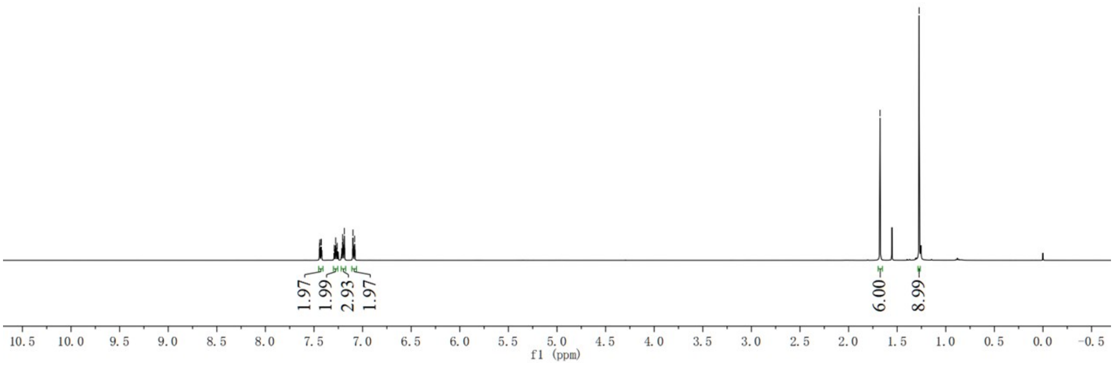

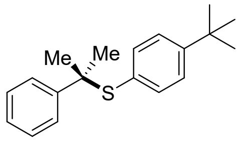

12

^{13}C NMR(126 MHz, $CDCl_3$)

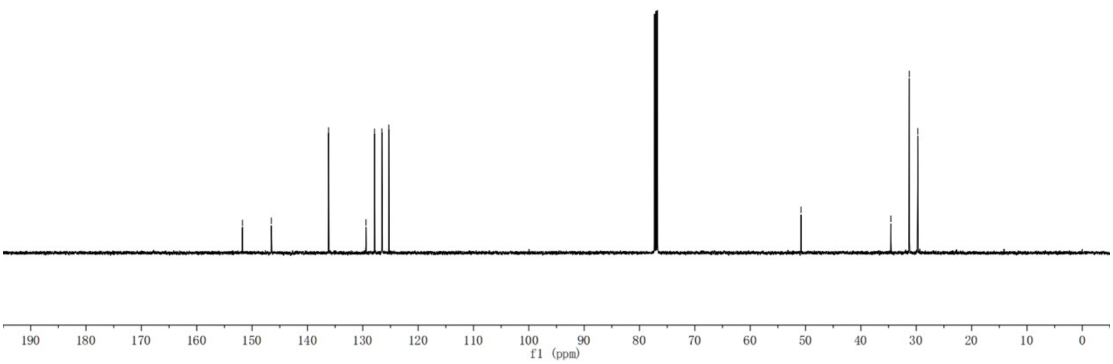

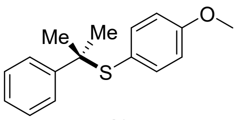

13

^{1}H NMR(500 MHz, $CDCl_3$)

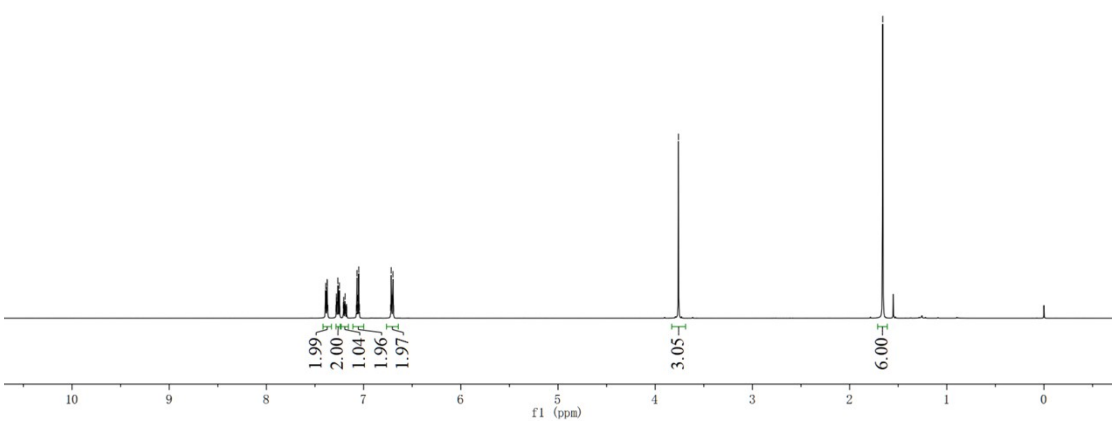

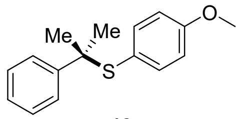

13

^{13}C NMR(126 MHz, $CDCl_3$)

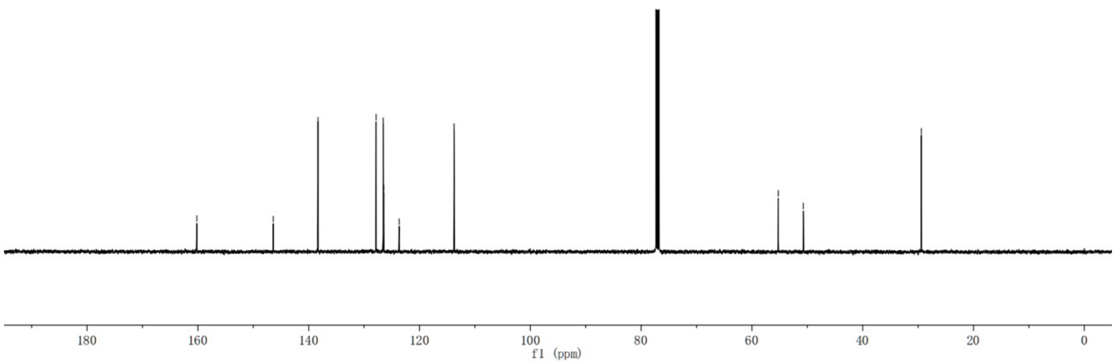

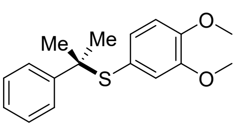

14

^{1}H NMR(500 MHz, $CDCl_3$)

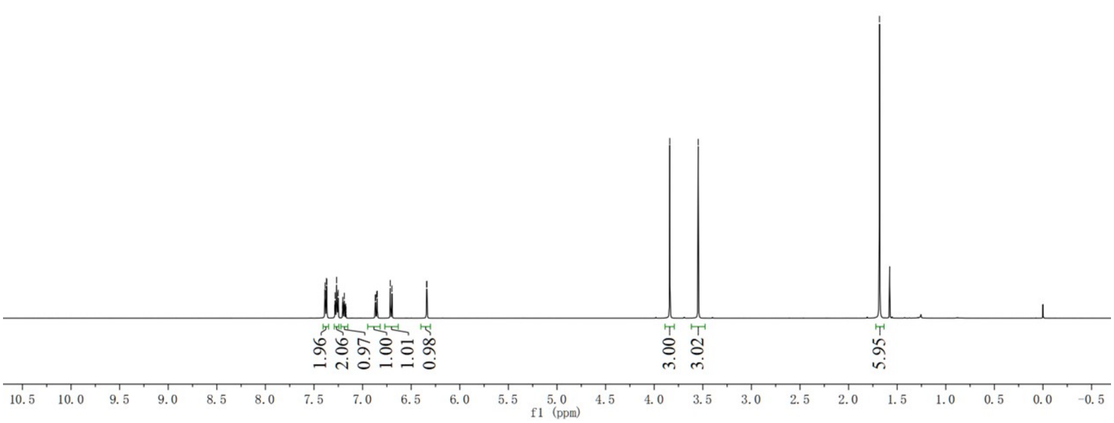

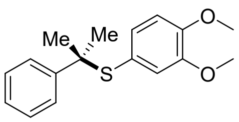

14

^{13}C NMR(126 MHz, $CDCl_3$)

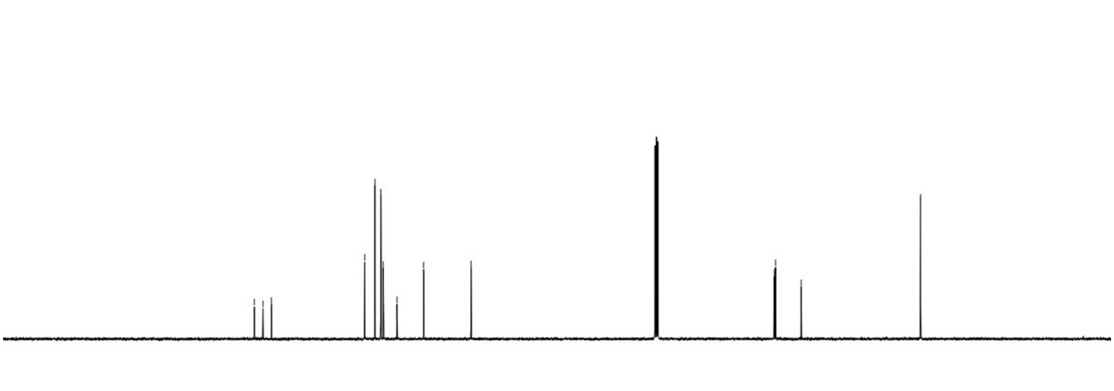

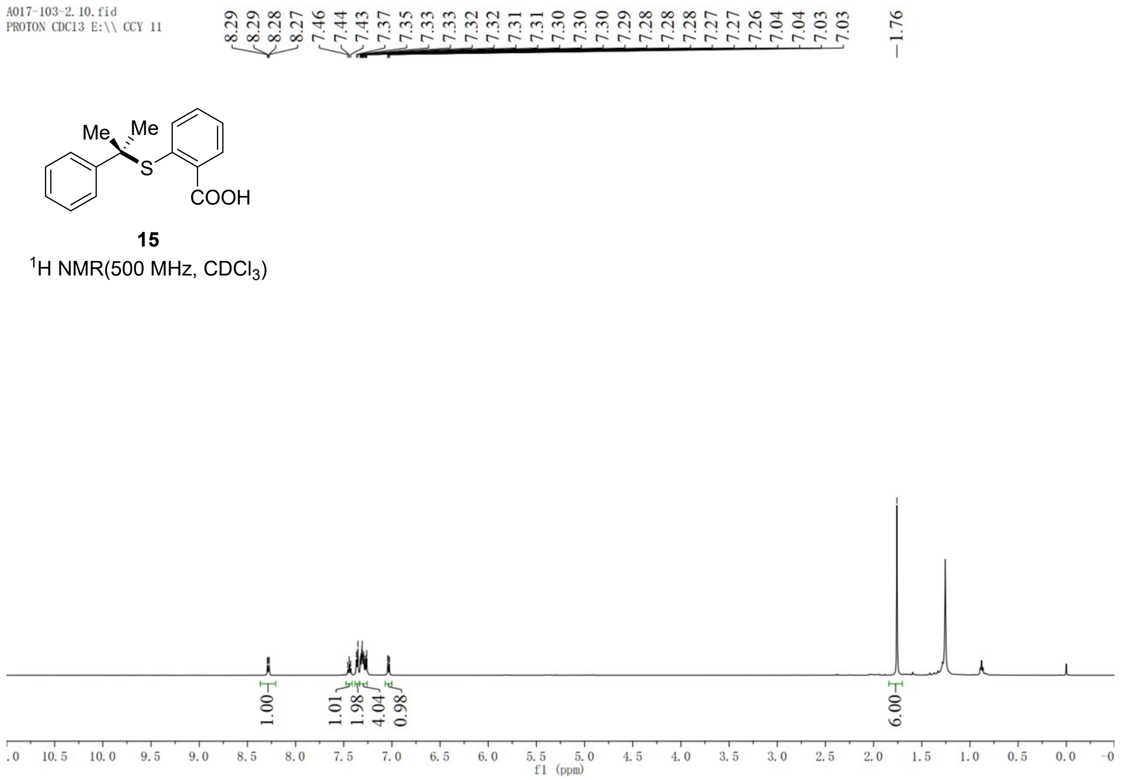
A017-103-2.10.fid
PROTON CDCl3 E:\\ CCY 11
8.29 8.29 8.28 8.27 7.46 7.44 7.43 7.37 7.35 7.33 7.33 7.32 7.32 7.31 7.31 7.30 7.30 7.30 7.29 7.28 7.28 7.28 7.27 7.27 7.26 7.04 7.04 7.03 7.03
1.76
Me
Me
S
COOH
15
^{1}H NMR(500 MHz, $CDCl_3$)
1.00
1.01
1.98
4.04
0.98
6.00
f1 (ppm)

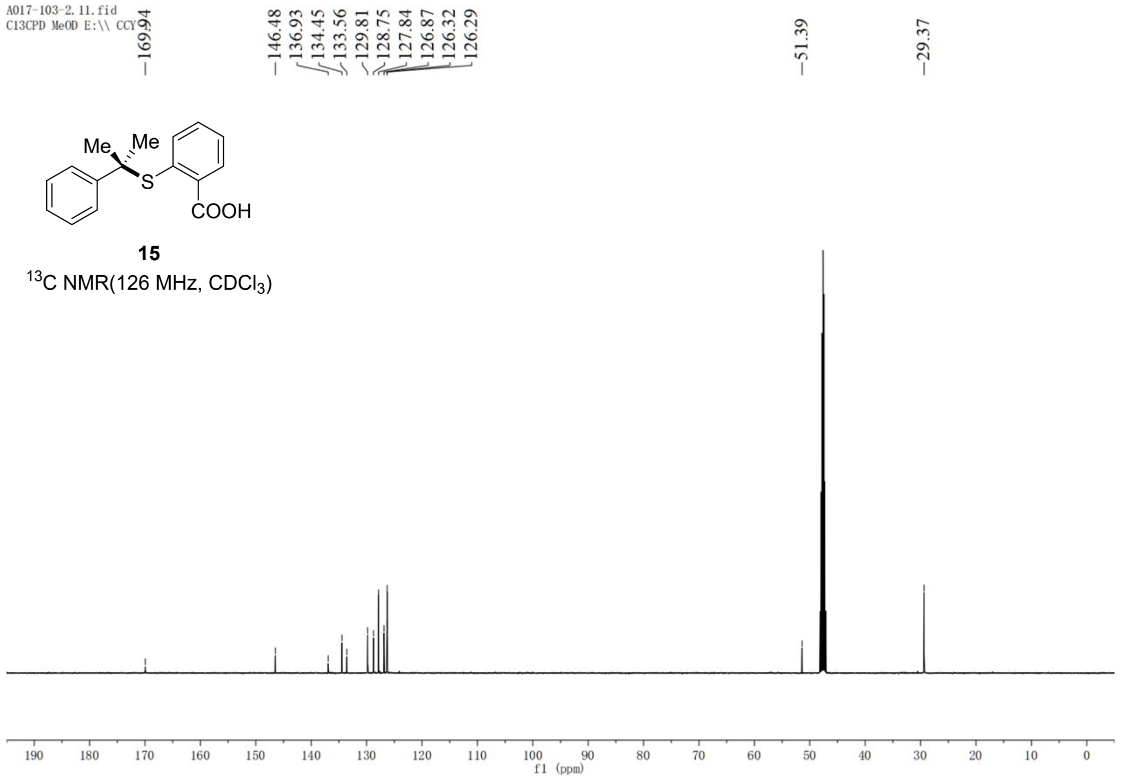
A017-103-2.11.fid
C13CPD MeOD E:\\ CCY
169.94
146.48 136.93 134.45 133.56 129.81 128.75 127.84 126.87 126.32 126.29
51.39
29.37
Me
Me
S
COOH
15
^{13}C NMR(126 MHz, $CDCl_3$)
f1 (ppm)

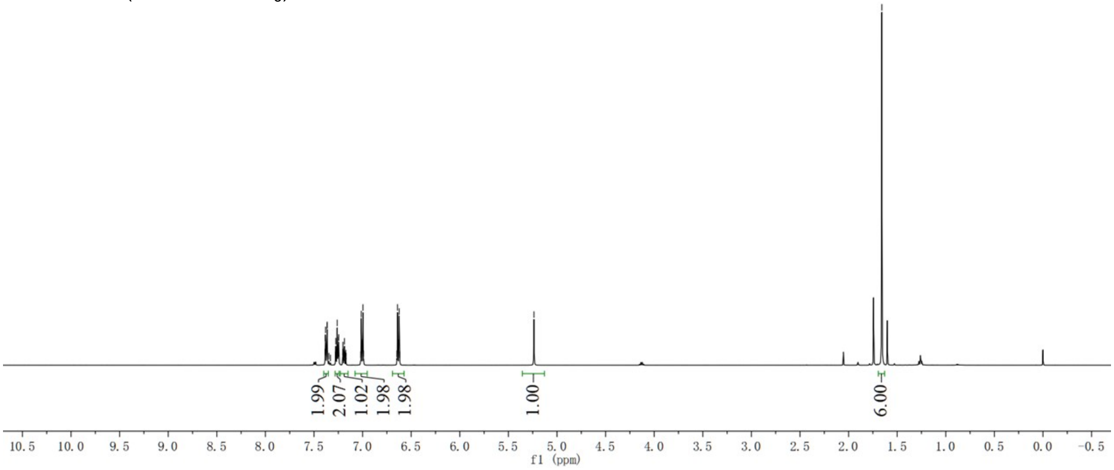

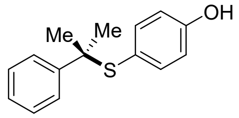

16

^{1}H NMR(500 MHz, $CDCl_3$)

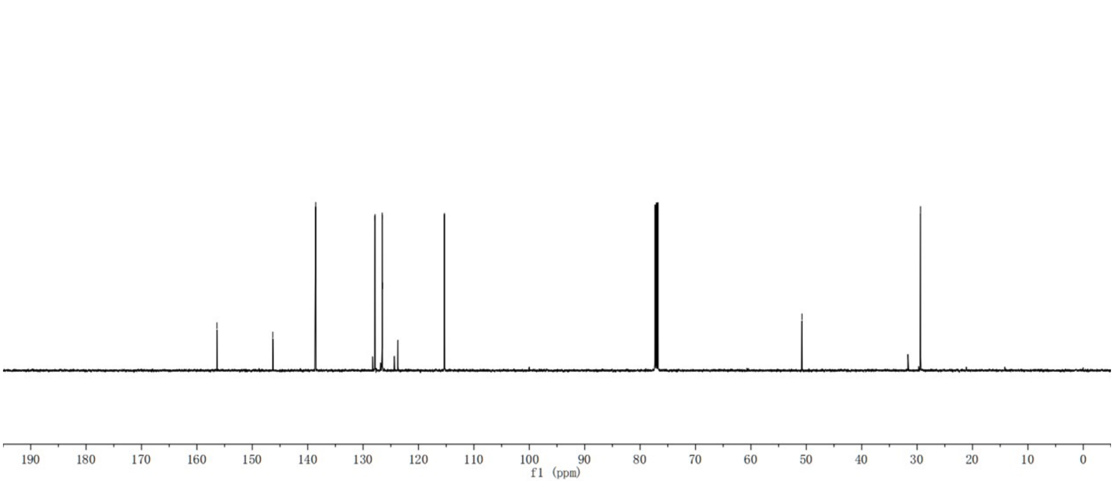

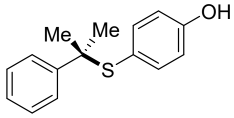

16

^{13}C NMR(126 MHz, $CDCl_3$)

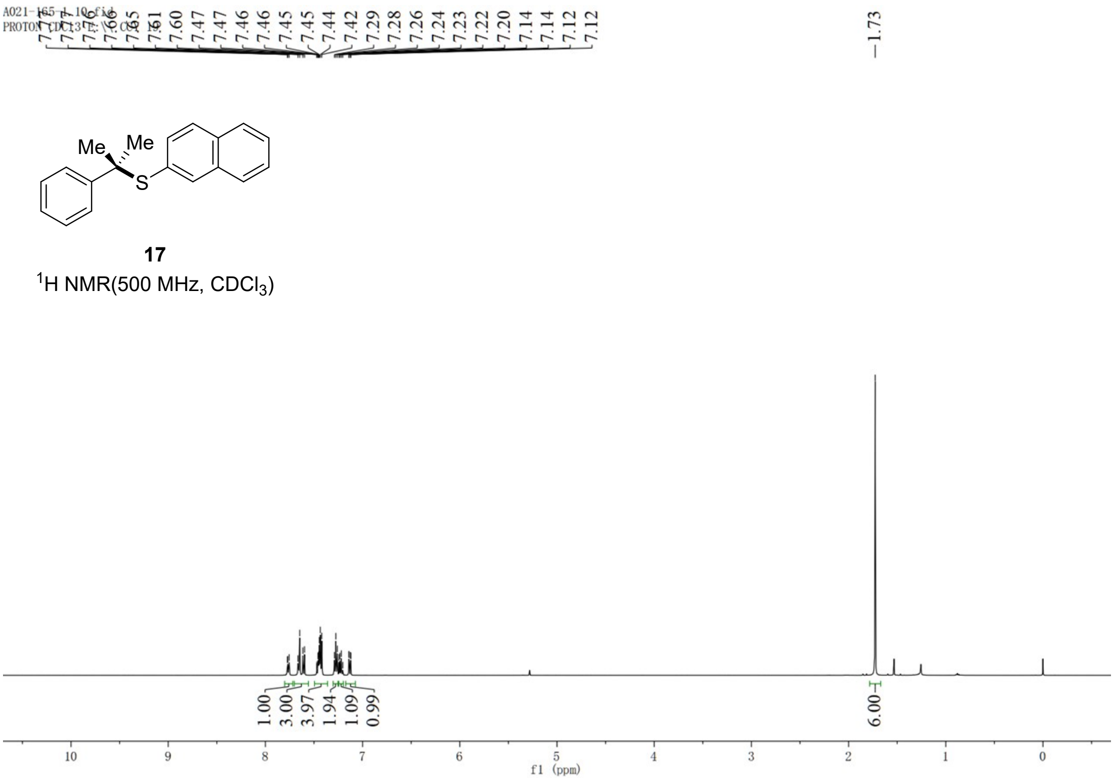

17

^{1}H NMR(500 MHz, $CDCl_3$)

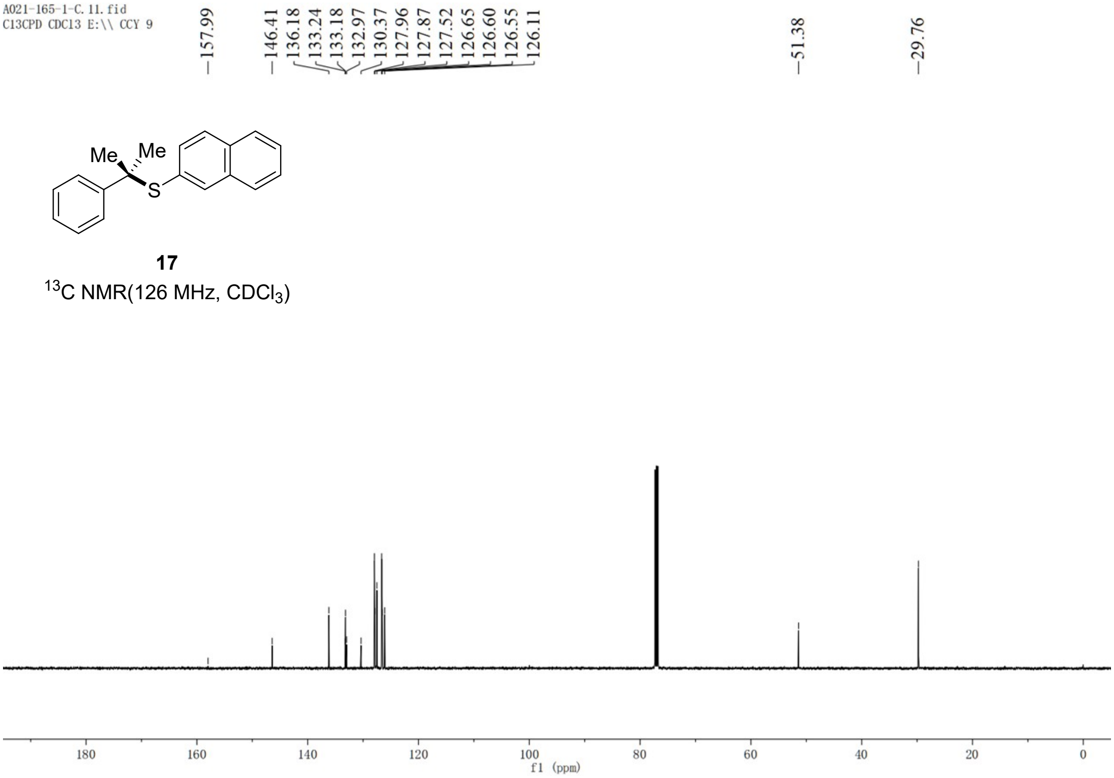

17

^{13}C NMR(126 MHz, $CDCl_3$)

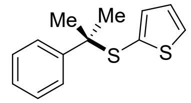

18

^{1}H NMR(500 MHz, $CDCl_3$)

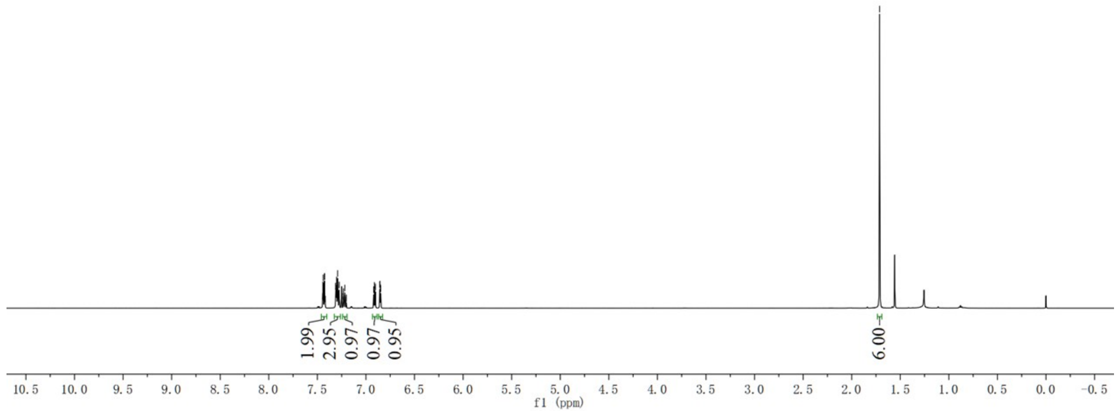

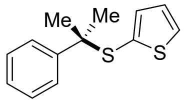

18

^{13}C NMR(126 MHz, $CDCl_3$)

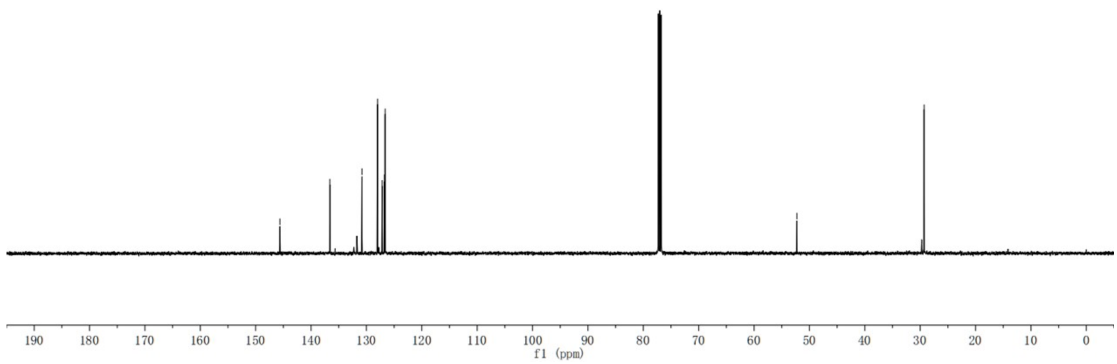

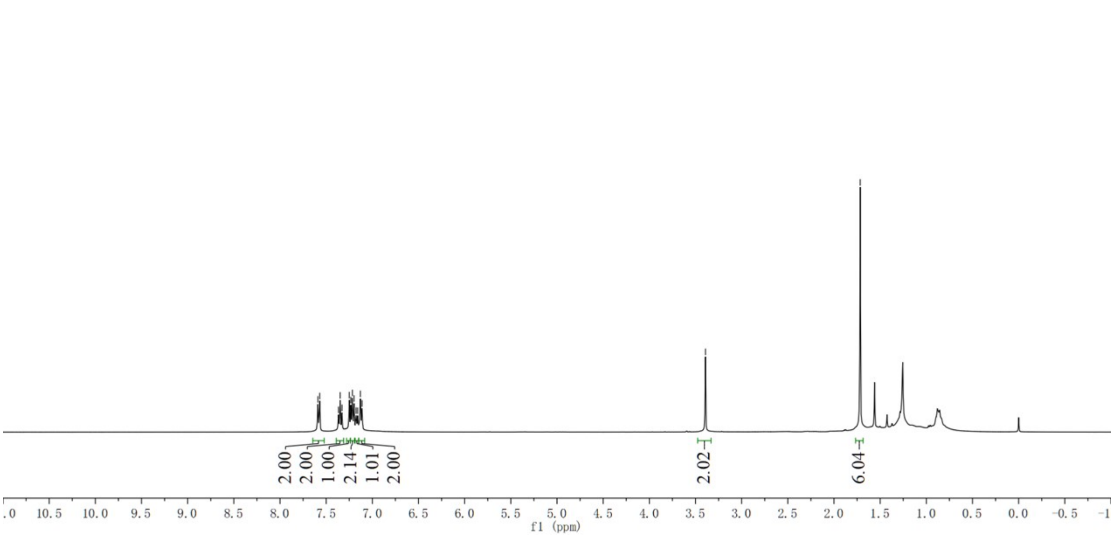

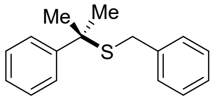

19

^{1}H NMR(500 MHz, $CDCl_3$)

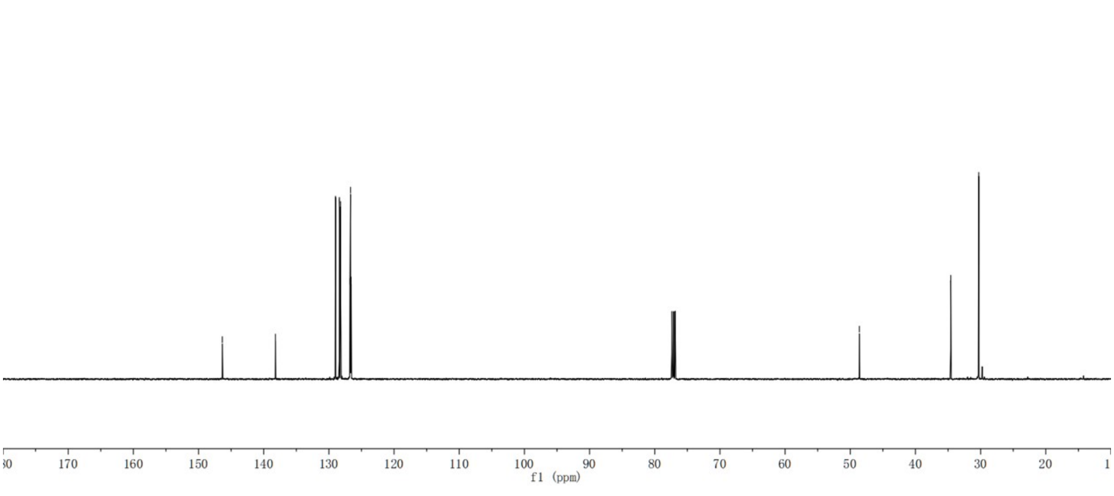

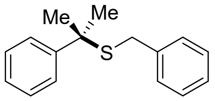

19

^{13}C NMR(126 MHz, $CDCl_3$)

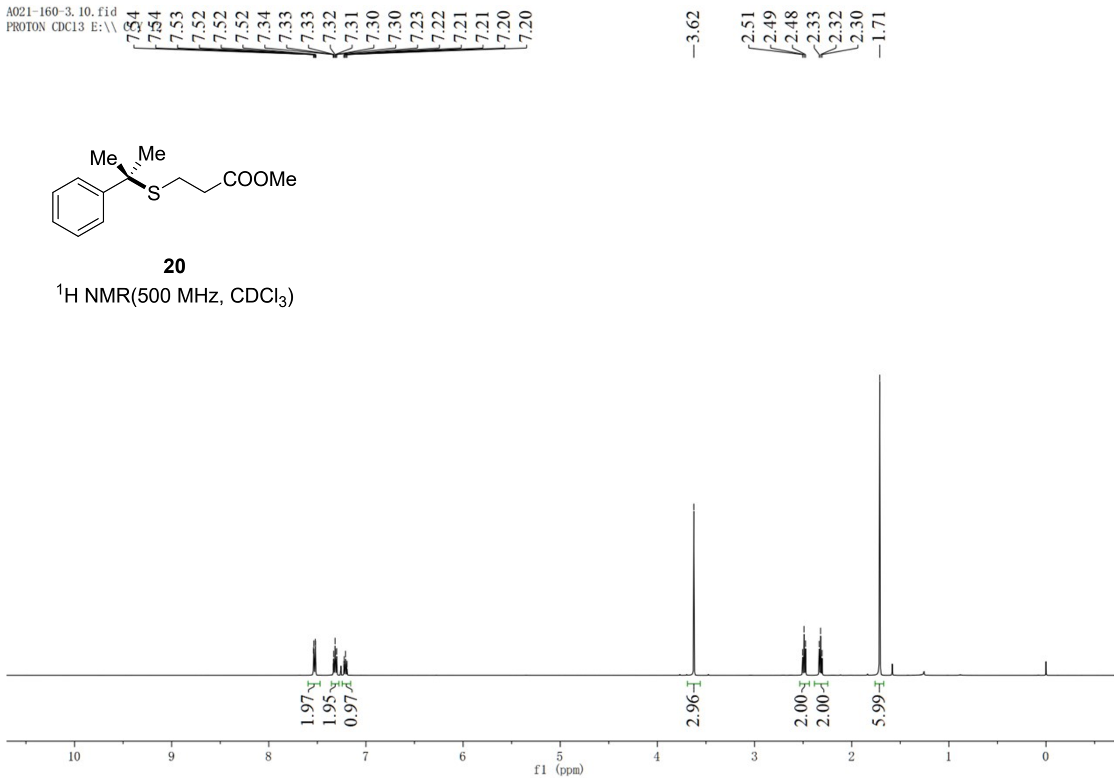

20

^{1}H NMR(500 MHz, $CDCl_3$)

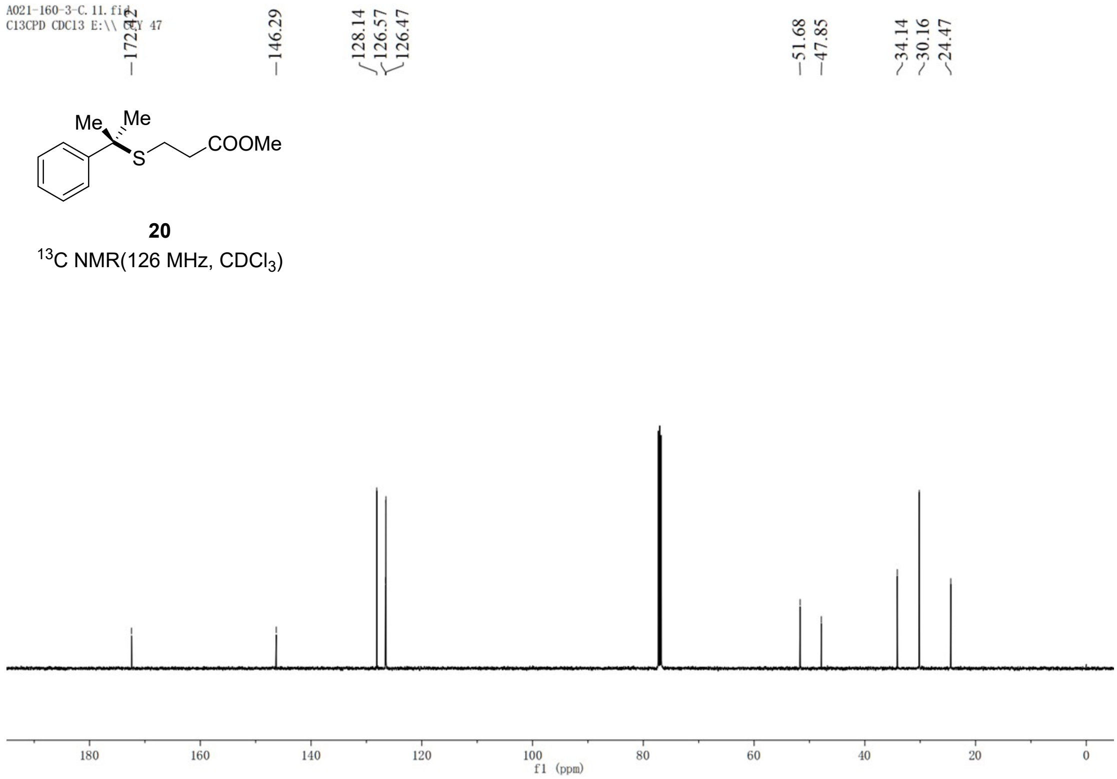

20

^{13}C NMR(126 MHz, $CDCl_3$)

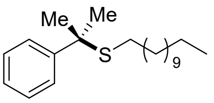

21
^1H NMR(500 MHz, $CDCl_3$)

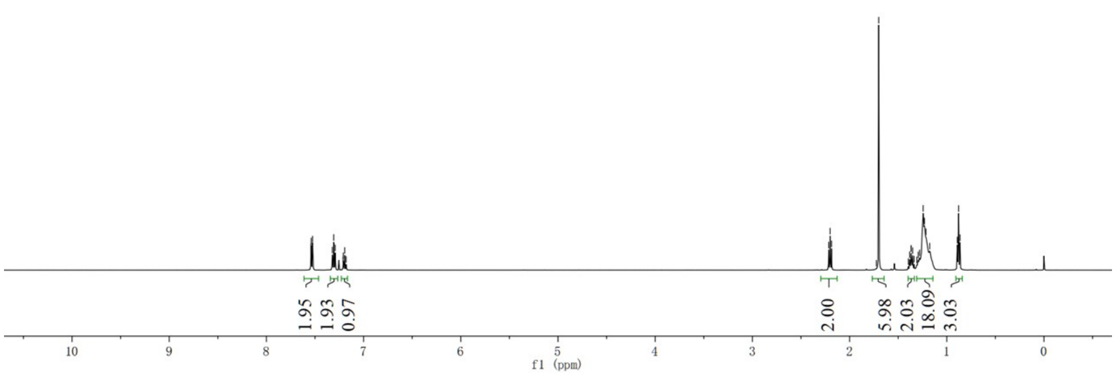

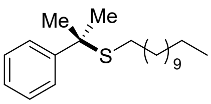

21
^{13}C NMR(126 MHz, $CDCl_3$)

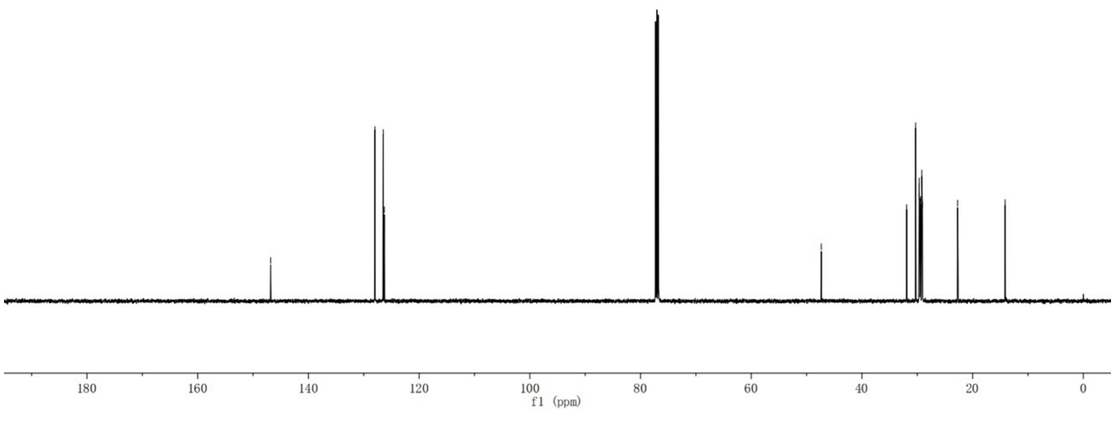

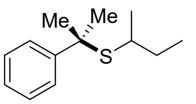

22

^{1}H NMR(500 MHz, $CDCl_3$)

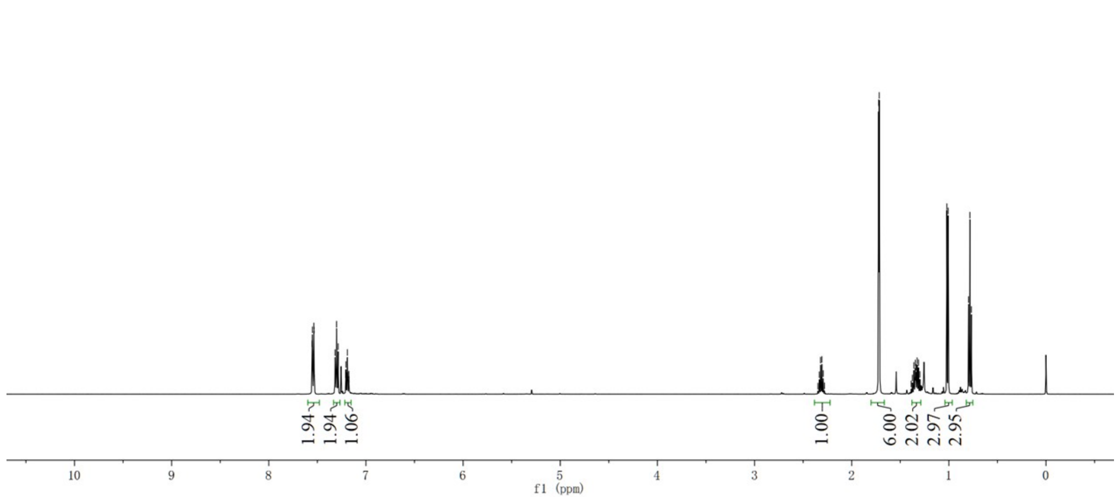

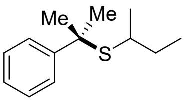

22

^{13}C NMR(126 MHz, $CDCl_3$)

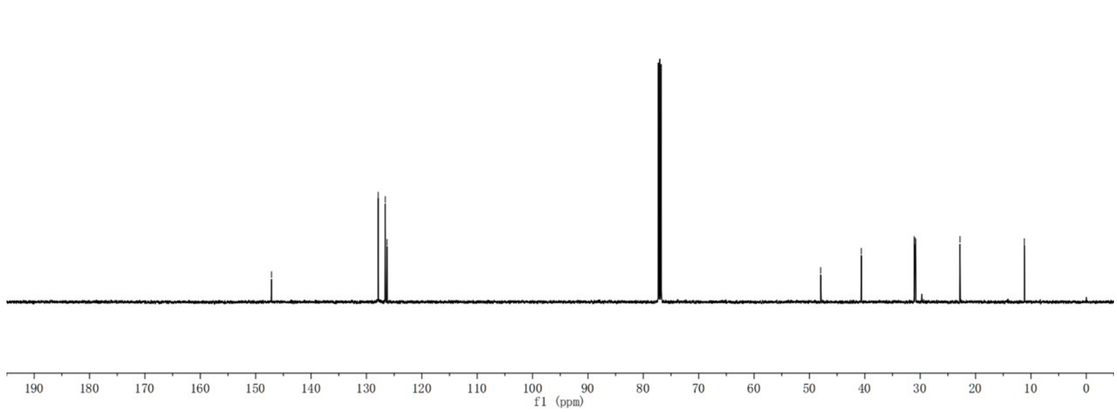

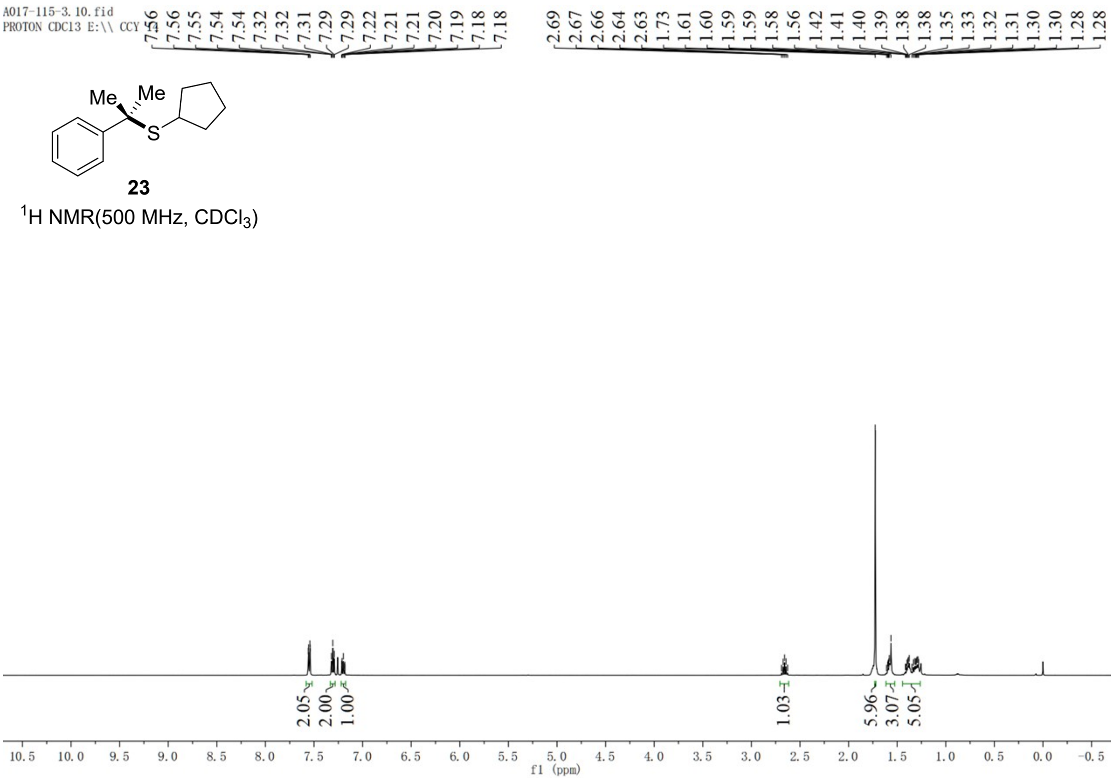

23

^1H NMR(500 MHz, $CDCl_3$)

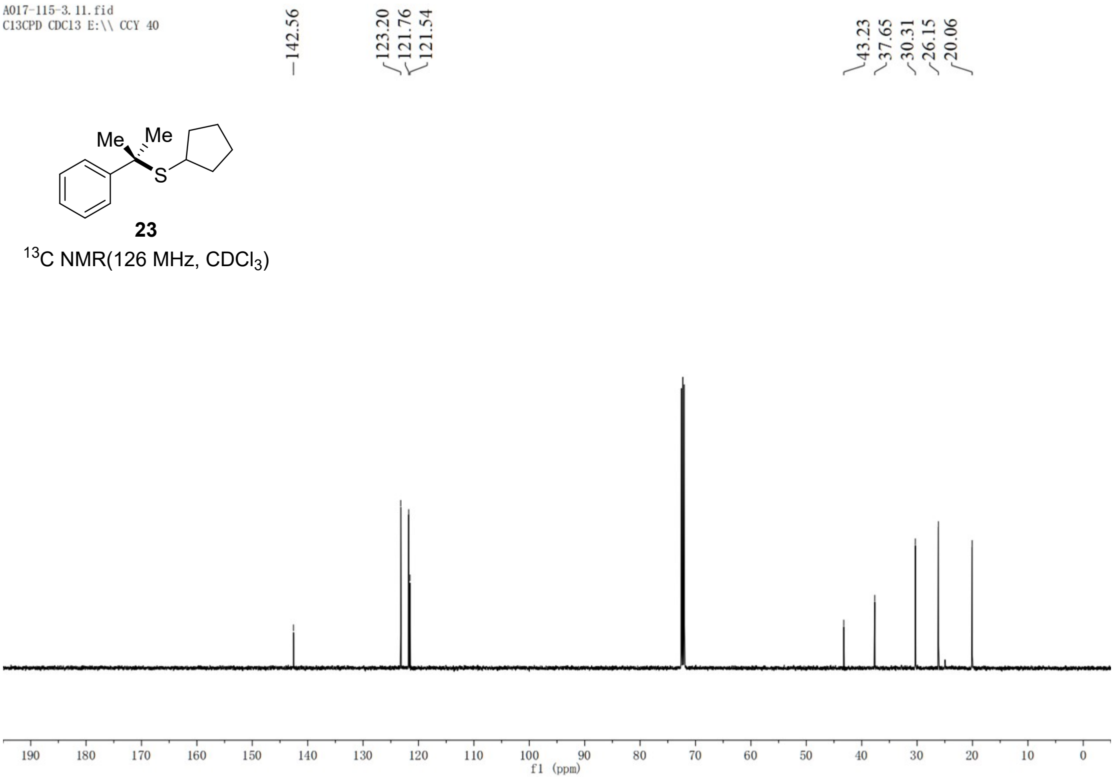

23

^{13}C NMR(126 MHz, $CDCl_3$)

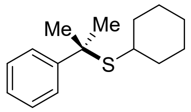

24

^{1}H NMR(500 MHz, $CDCl_3$)

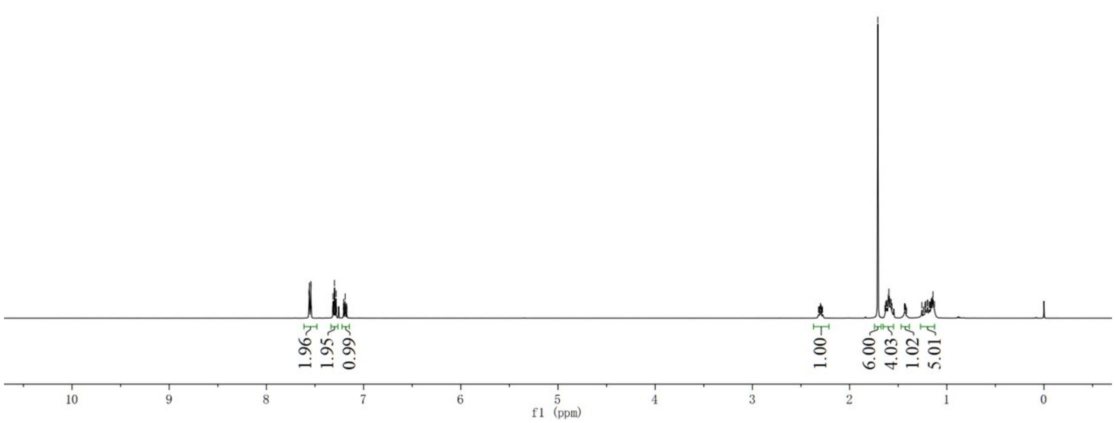

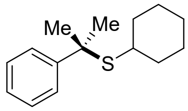

24

^{13}C NMR(126 MHz, $CDCl_3$)

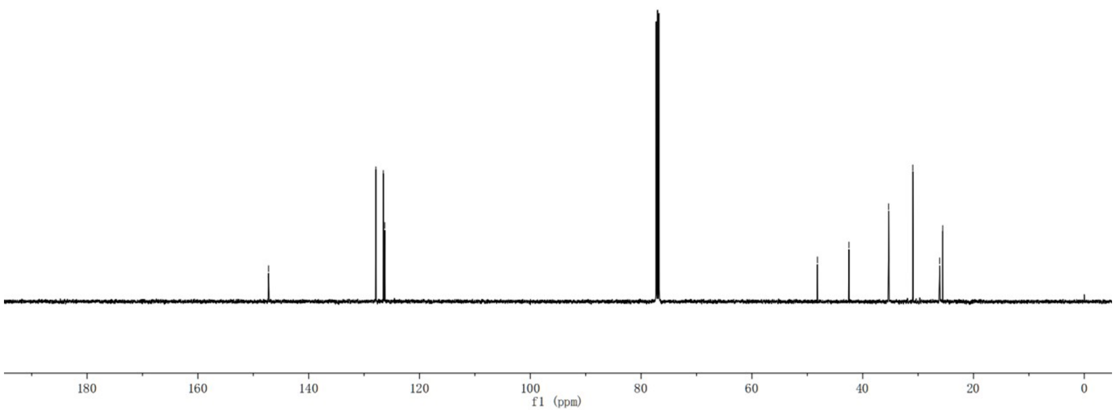

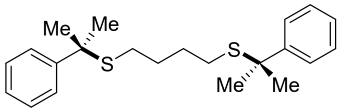

25

^{1}H NMR(500 MHz, $CDCl_3$)

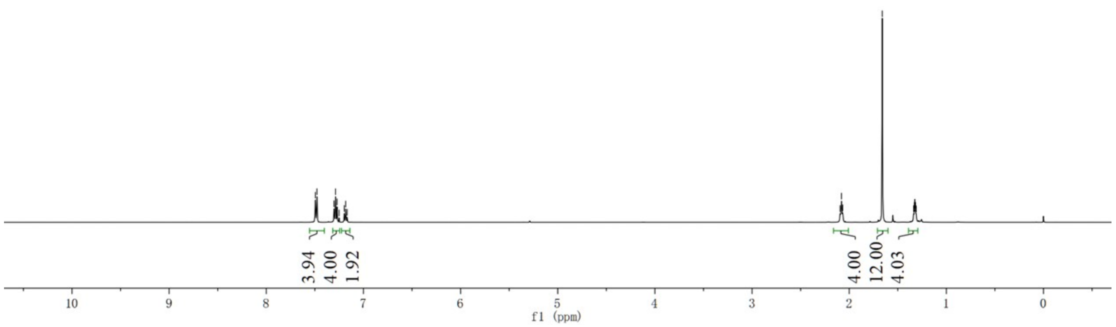

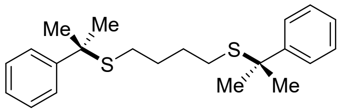

25

^{13}C NMR(126 MHz, $CDCl_3$)

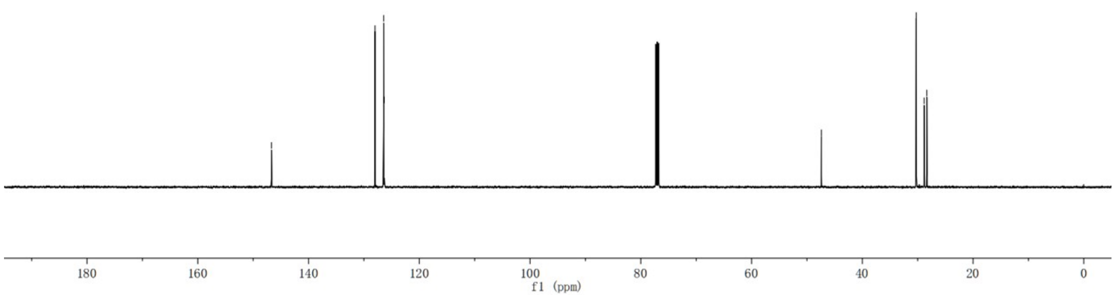

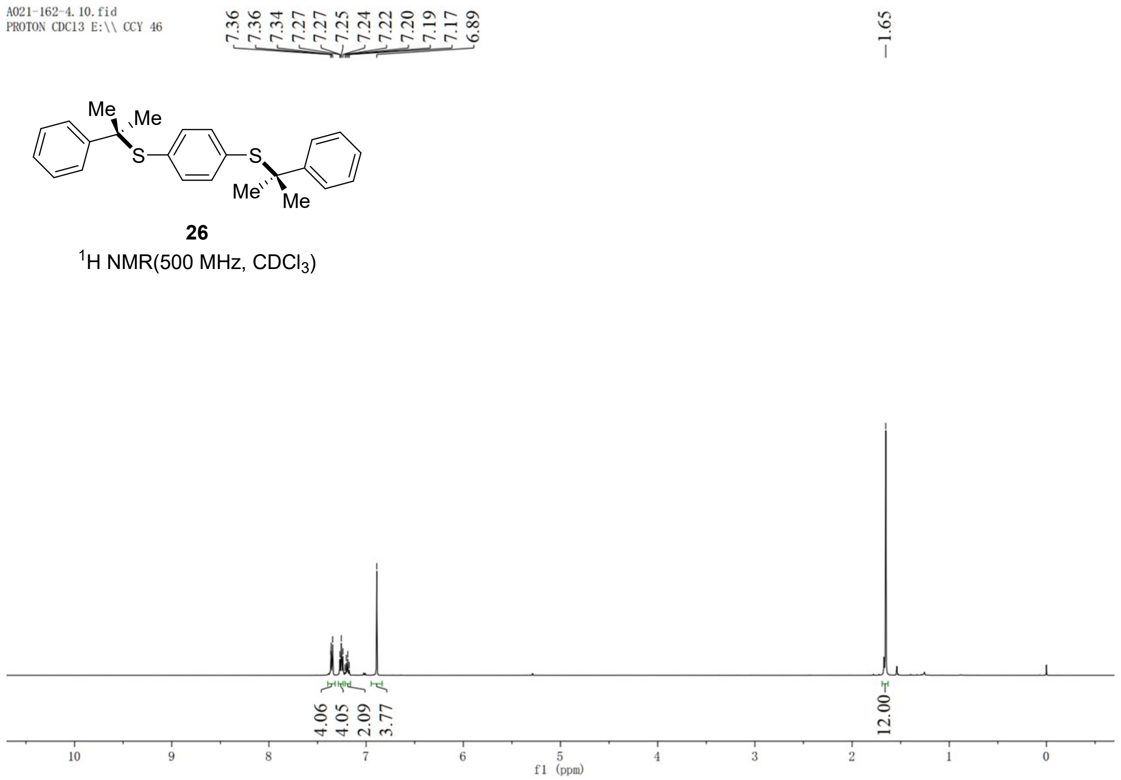
A021-162-4.10.fid
PROTON CDCl3 E:\\ CCY 46
7.36
7.36
7.34
7.27
7.27
7.25
7.24
7.22
7.20
7.19
7.17
6.89
1.65
Me
Me
S
S
Me
Me
26
^{1}H NMR(500 MHz, $CDCl_3$)
4.06
4.05
2.09
3.77
12.00
f1 (ppm)

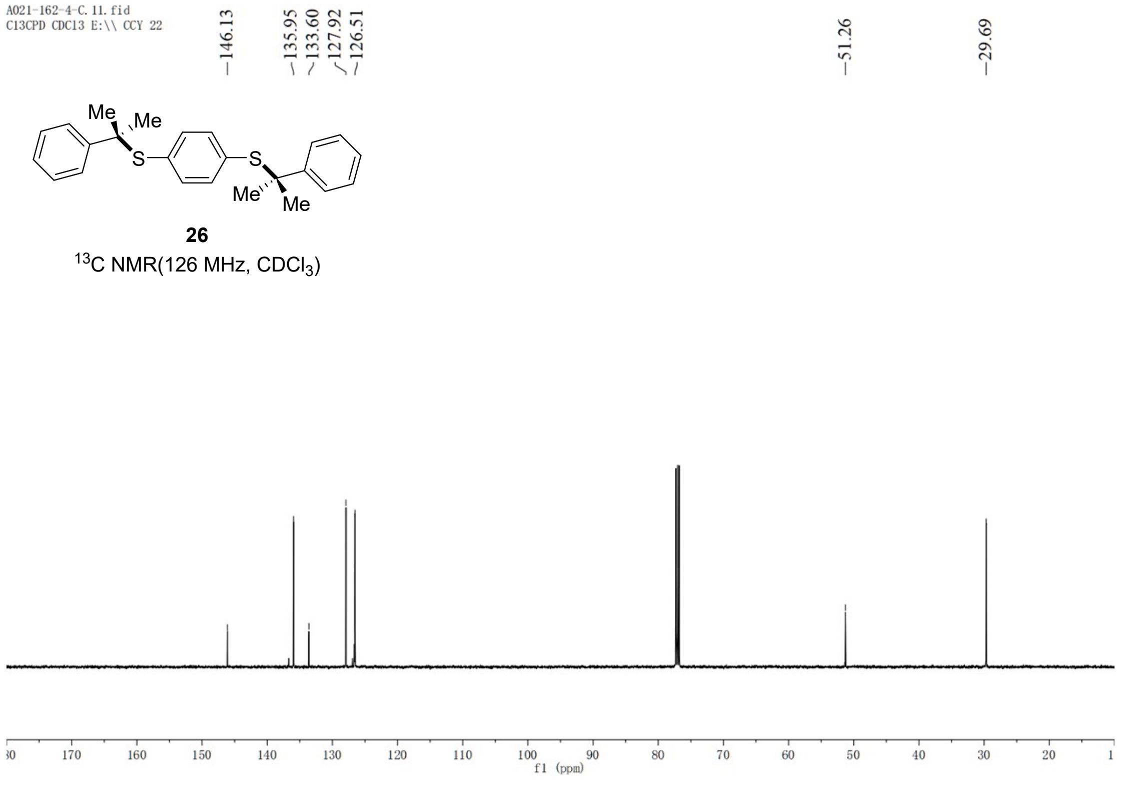
A021-162-4-C.11.fid
C13CPD CDCl3 E:\\ CCY 22
146.13
135.95
133.60
127.92
126.51
51.26
29.69
Me
Me
S
S
Me
Me
26
^{13}C NMR(126 MHz, $CDCl_3$)
f1 (ppm)

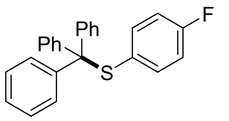

27

^{1}H NMR(500 MHz, $CDCl_3$)

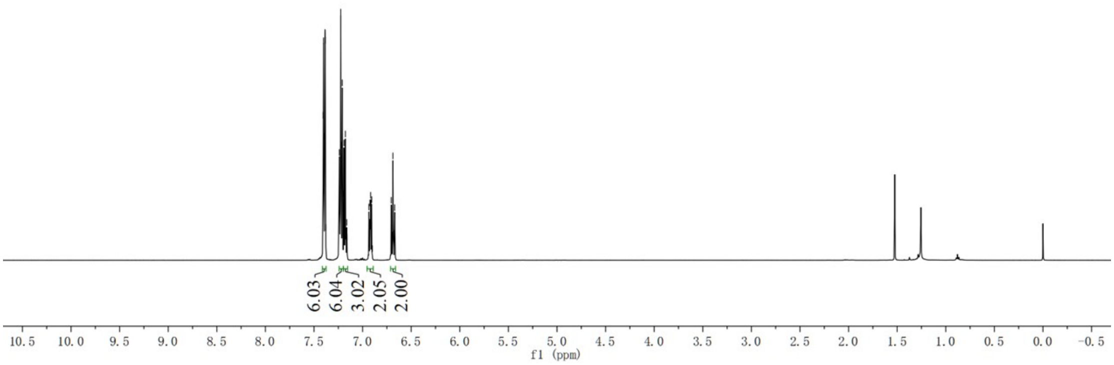

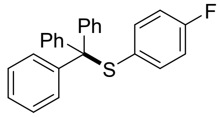

27

^{13}C NMR(126 MHz, $CDCl_3$)

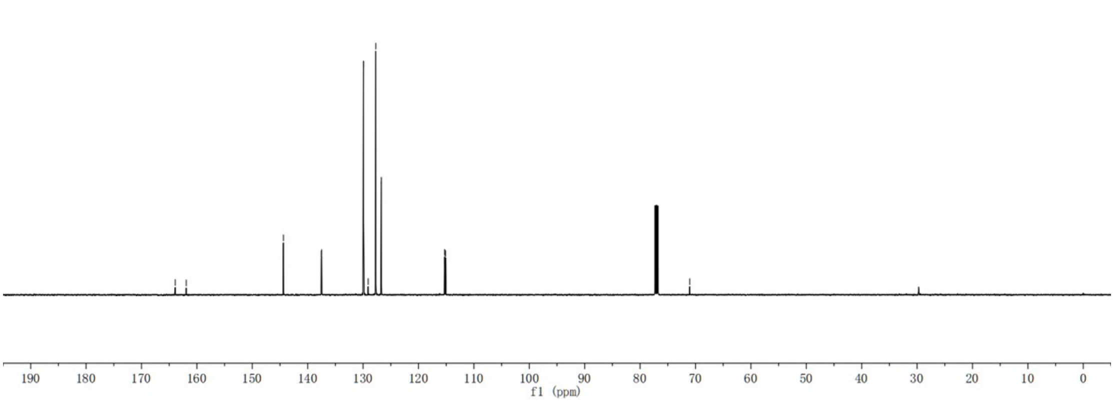

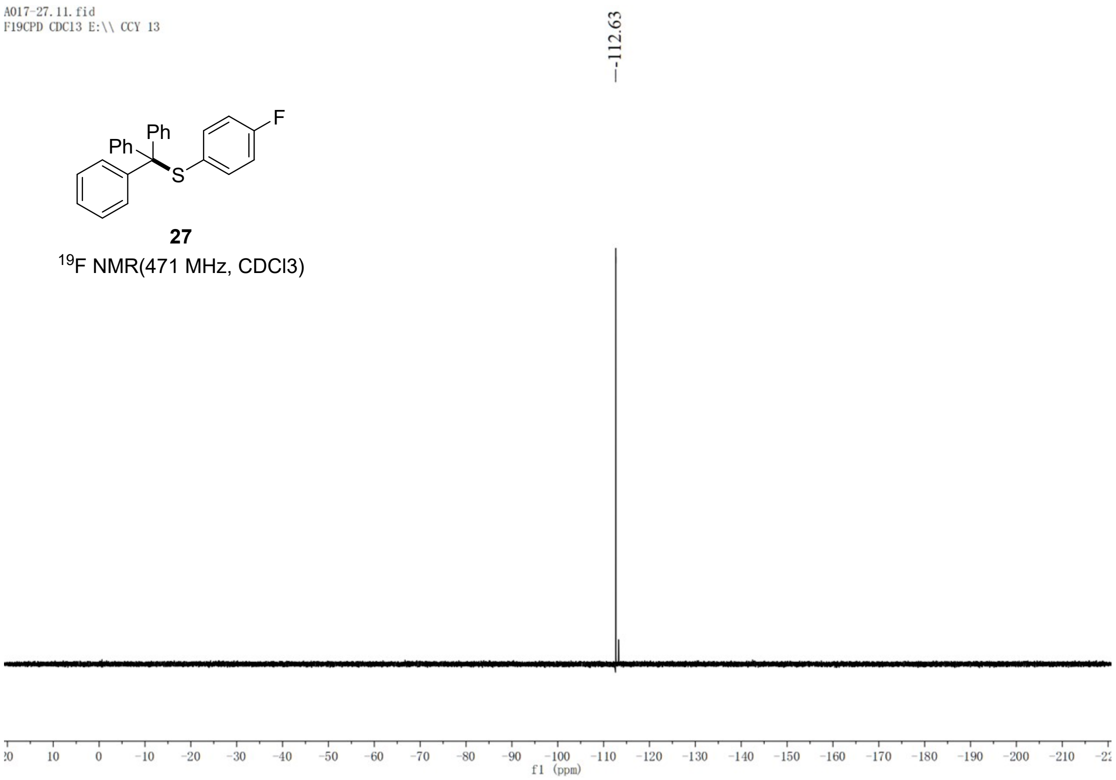
A017-27.11.fid
F19CPD CDCl3 E:\\ CCY 13
-112.63
Ph
Ph
S
F
27
19F NMR(471 MHz, CDCl3)
f1 (ppm)

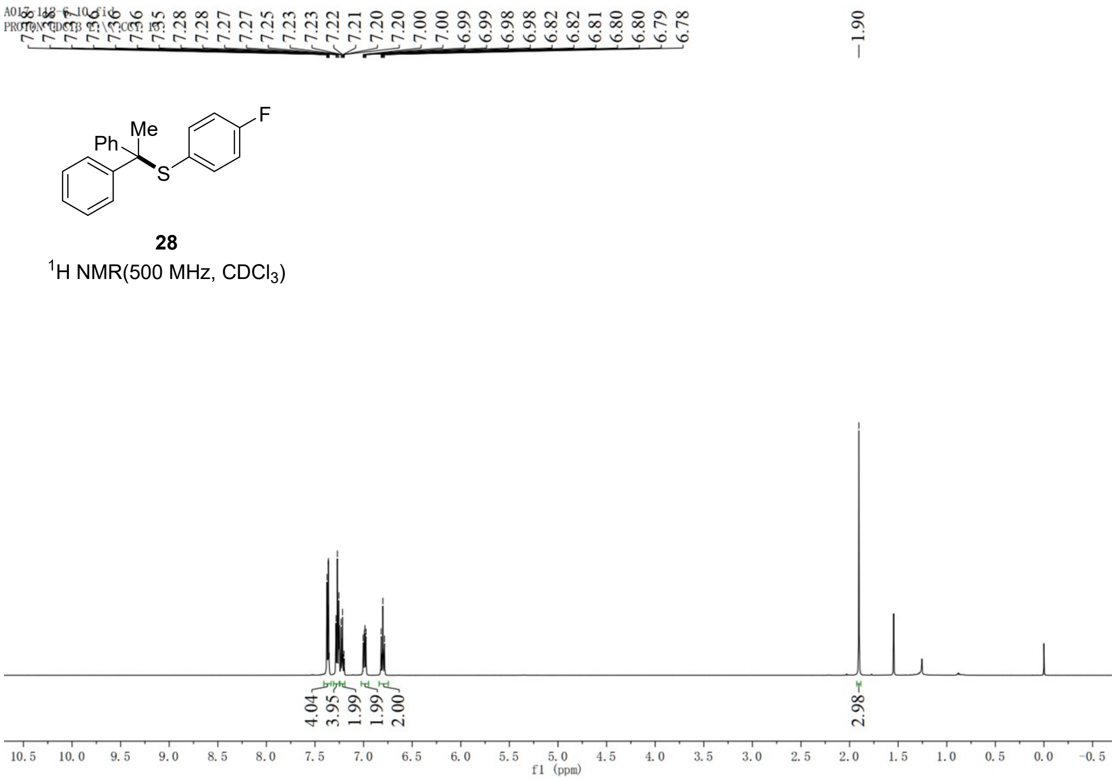

28

^{1}H NMR(500 MHz, $CDCl_3$)

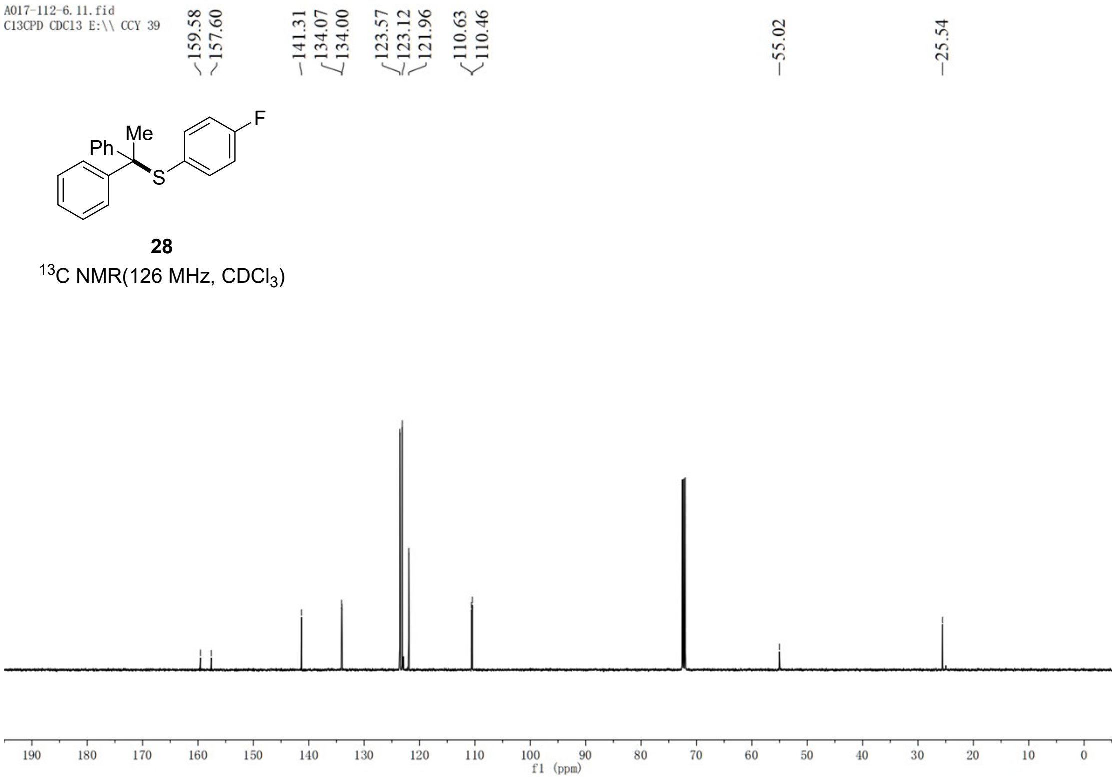

28

^{13}C NMR(126 MHz, $CDCl_3$)

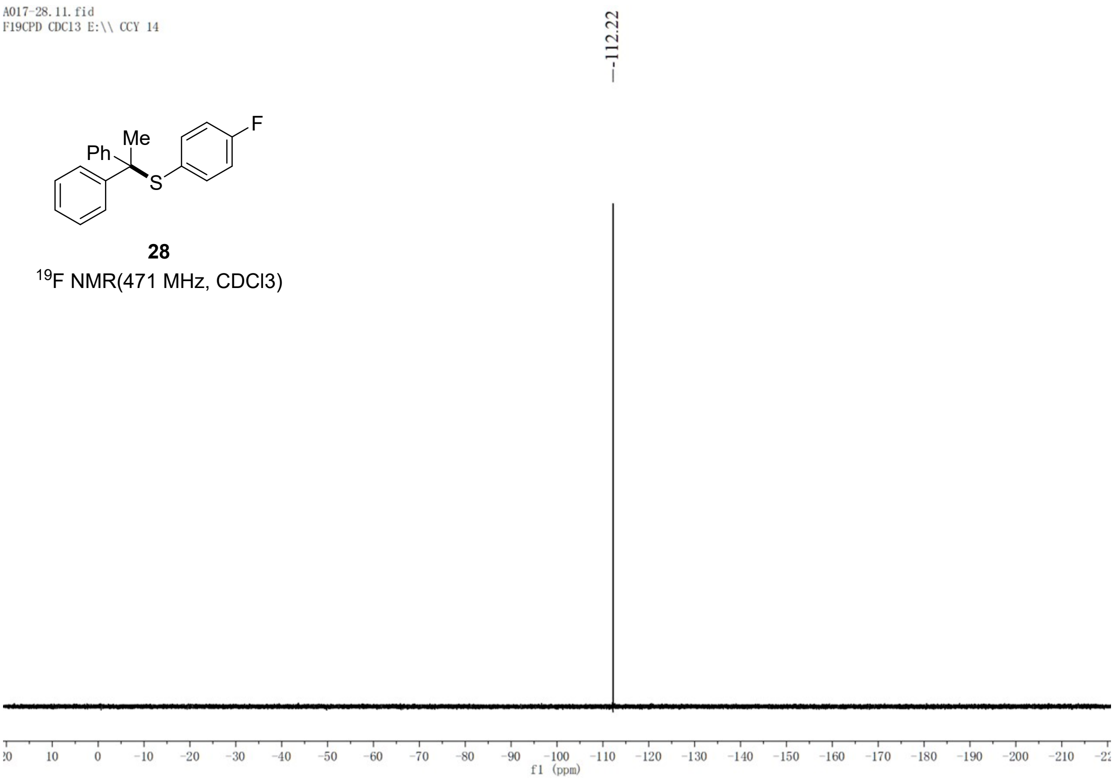

28

^{19}F NMR(471 MHz, CDCl3)

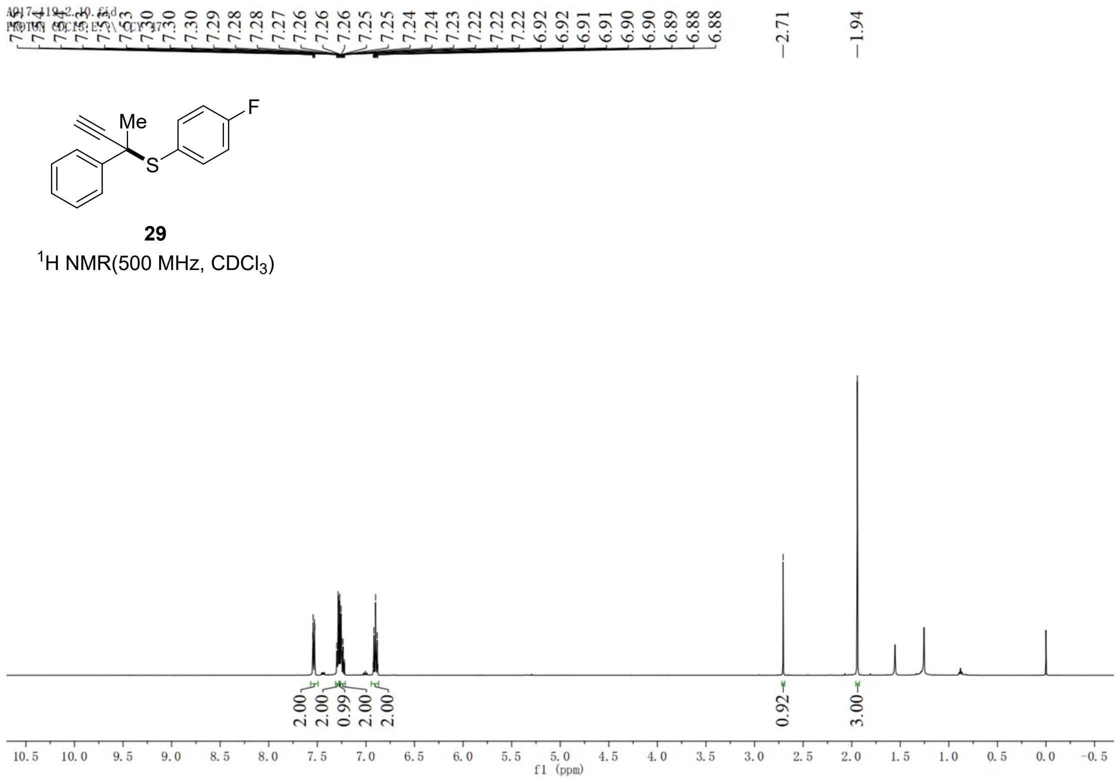

29

^{1}H NMR(500 MHz, $CDCl_3$)

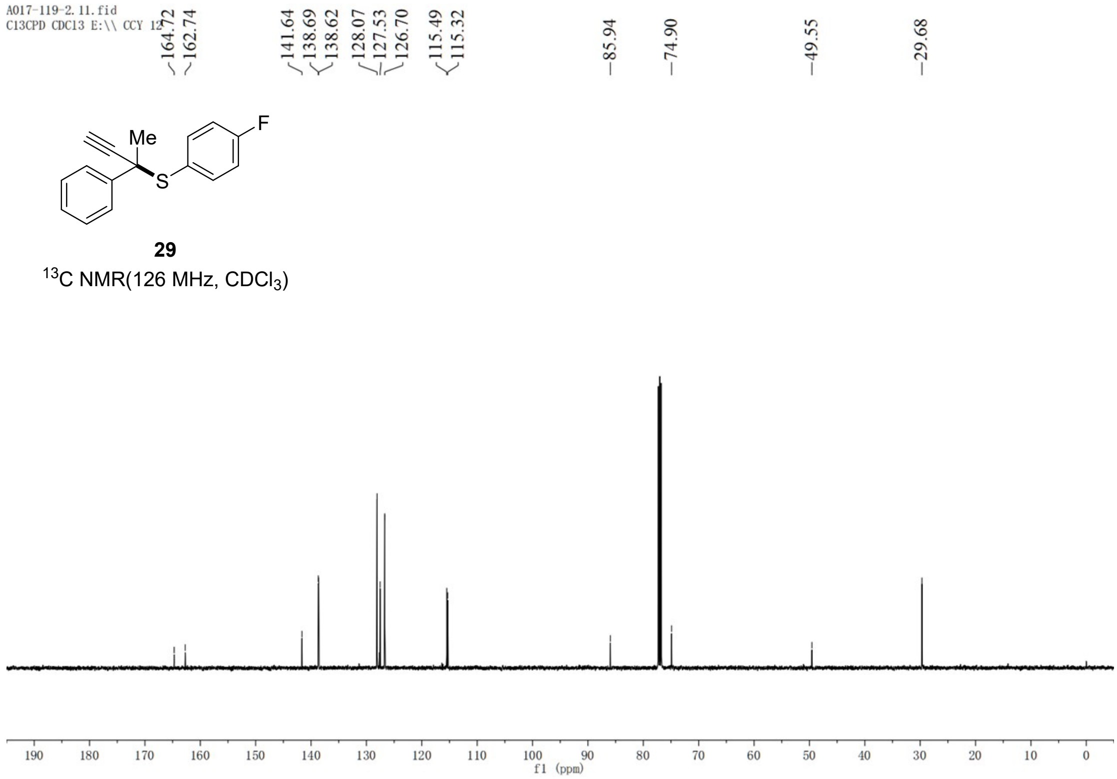

29

^{13}C NMR(126 MHz, $CDCl_3$)

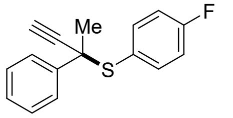

29

^{19}F NMR(471 MHz, CDCl3)

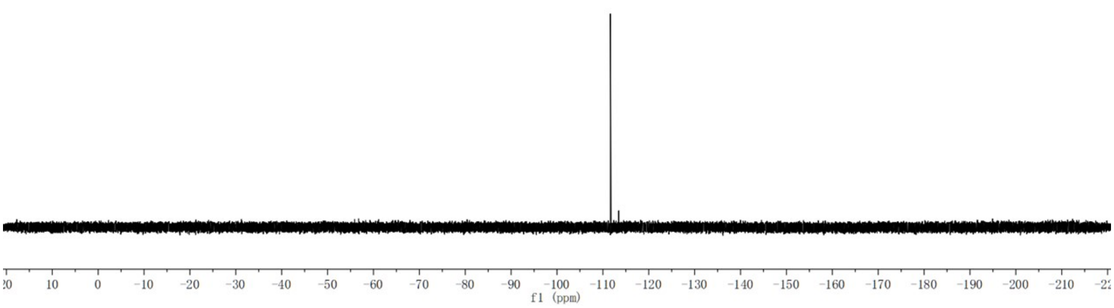

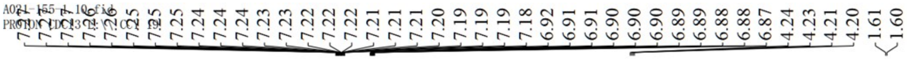

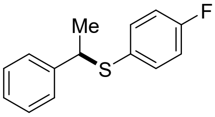

30

^{1}H NMR(500 MHz, $CDCl_3$)

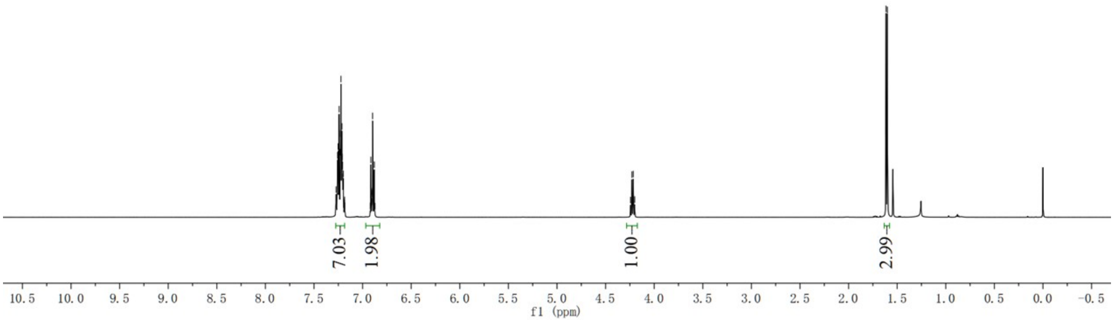

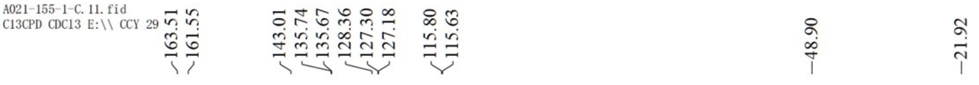

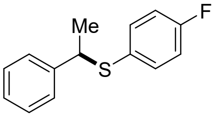

30

^{13}C NMR(126 MHz, $CDCl_3$)

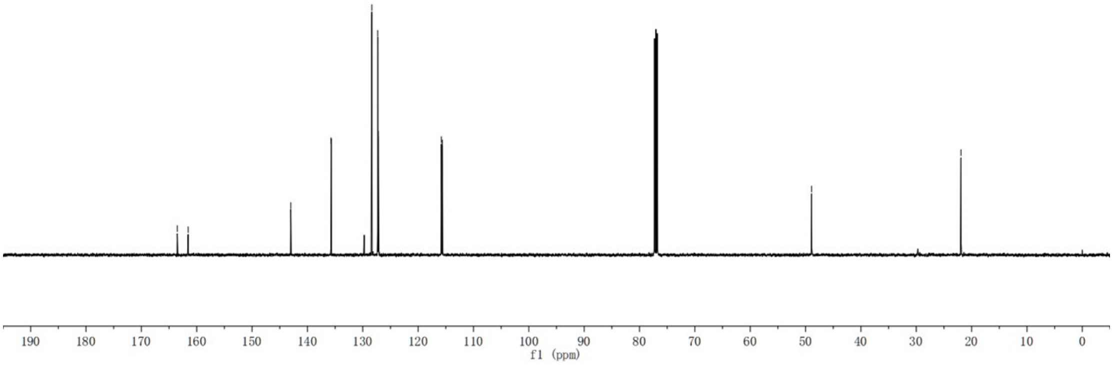

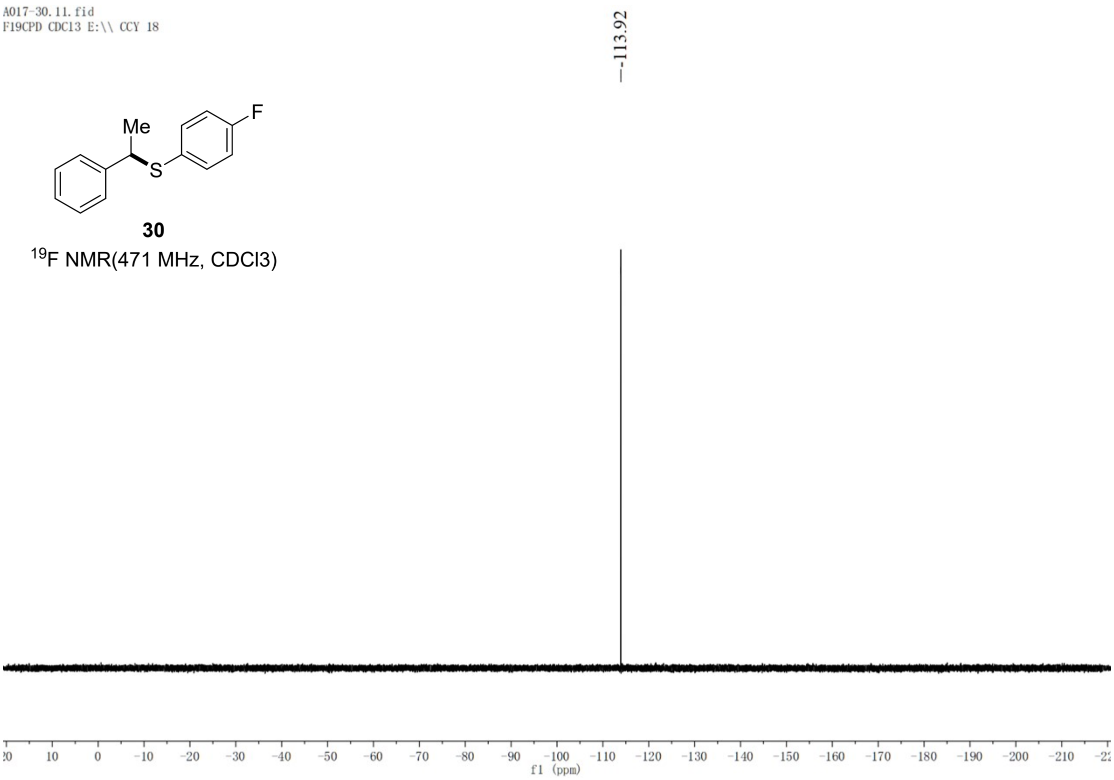

^{19}F NMR(471 MHz, CDCl3)

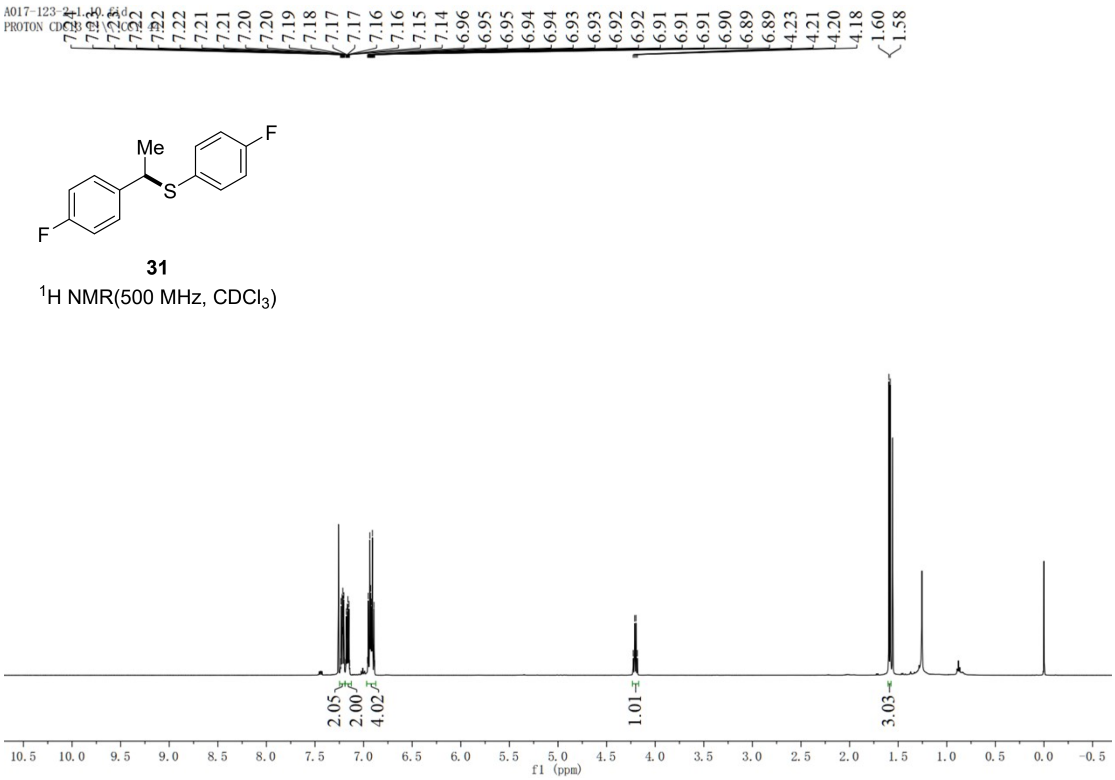

31

^{1}H NMR(500 MHz, $CDCl_3$)

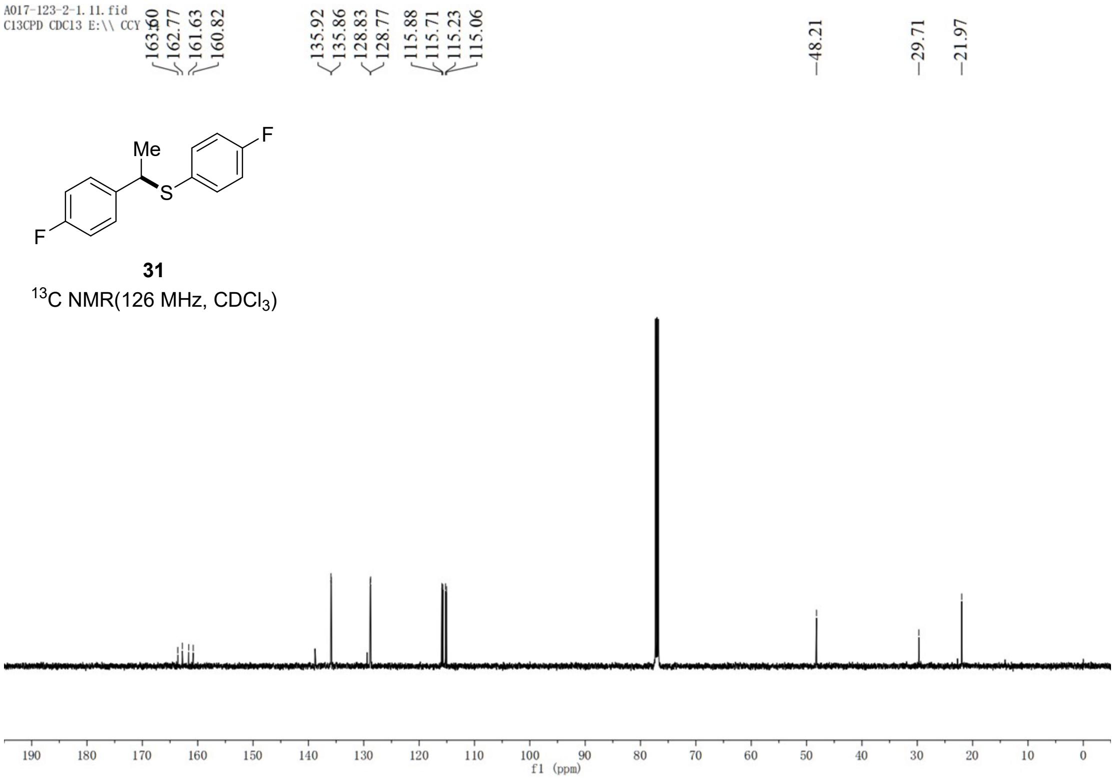

31

^{13}C NMR(126 MHz, $CDCl_3$)

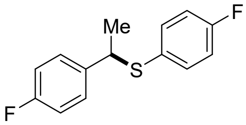

31

^{19}F NMR(471 MHz, CDCl3)

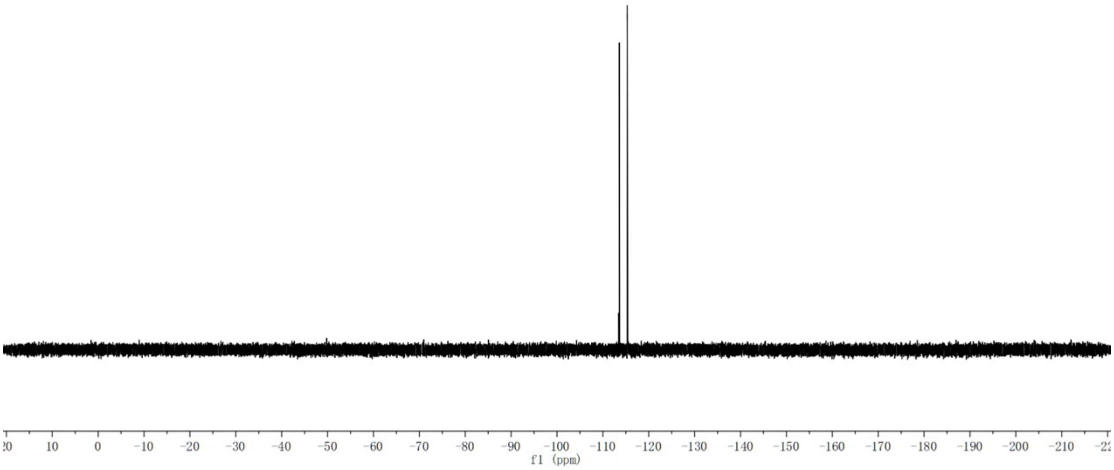

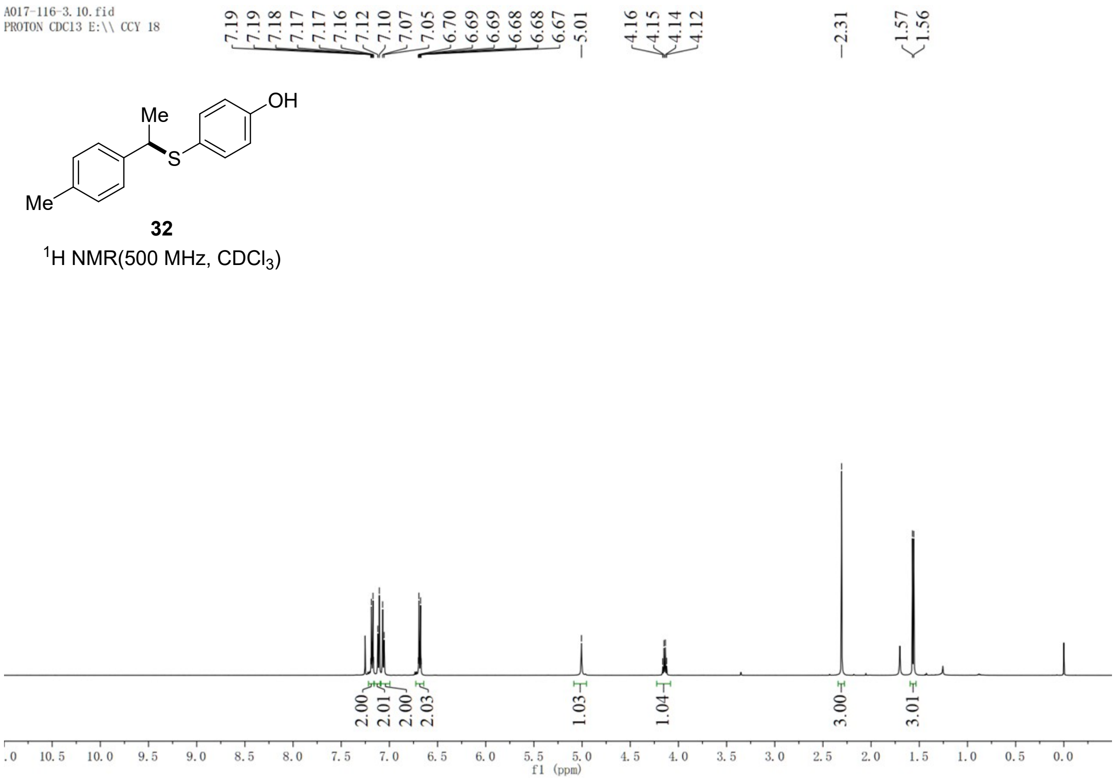
A017-116-3.10.fid
PROTON CDCl3 E:\\ CCY 18
7.19
7.19
7.18
7.17
7.17
7.16
7.12
7.10
7.07
7.05
6.70
6.69
6.69
6.68
6.68
6.67
5.01
4.16
4.15
4.14
4.12
2.31
1.57
1.56
Me
OH
S
Me
32
1H NMR(500 MHz, CDCl3)
2.00
2.01
2.00
2.03
1.03
1.04
3.00
3.01
f1 (ppm)

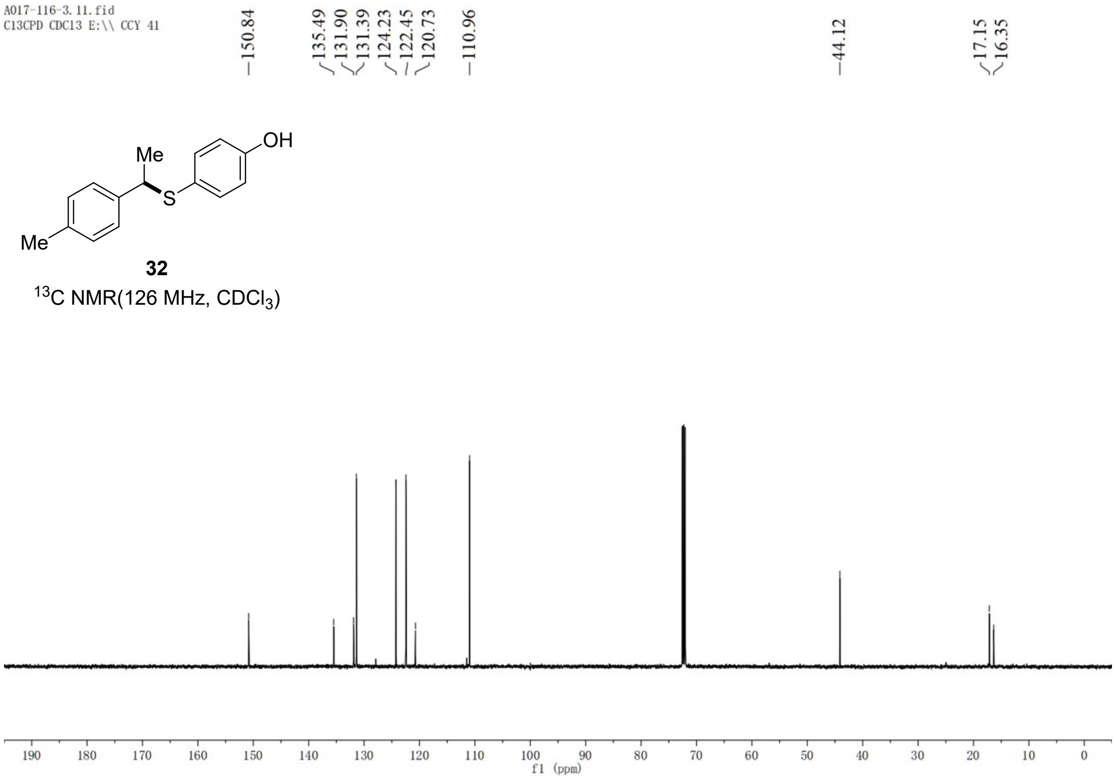
A017-116-3.11.fid
C13CPD CDCl3 E:\\ CCY 41
150.84
135.49
131.90
131.39
124.23
122.45
120.73
110.96
44.12
17.15
16.35
Me
OH
S
Me
32
13C NMR(126 MHz, CDCl3)
f1 (ppm)

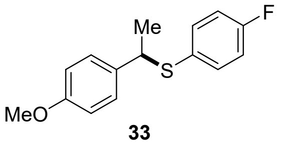

33

^{1}H NMR(500 MHz, $CDCl_3$)

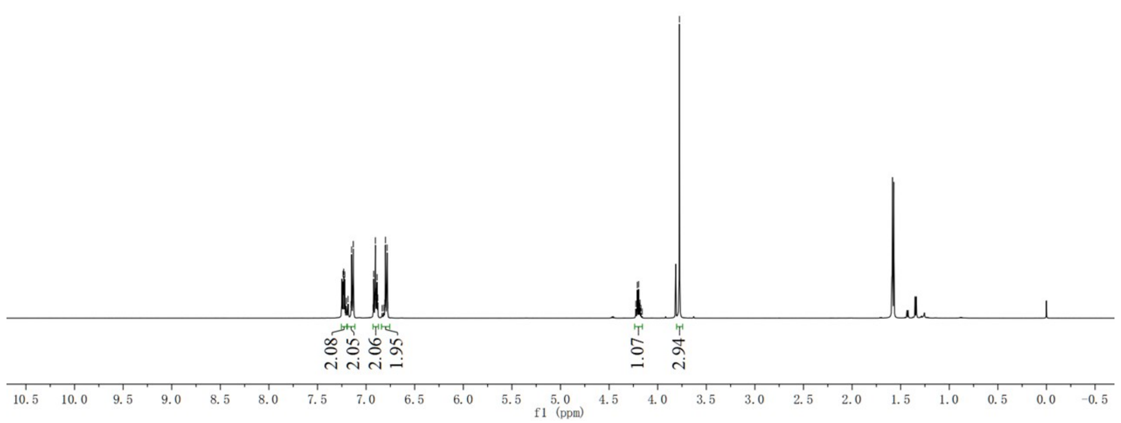

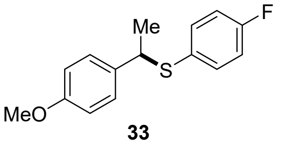

33

^{13}C NMR(126 MHz, $CDCl_3$)

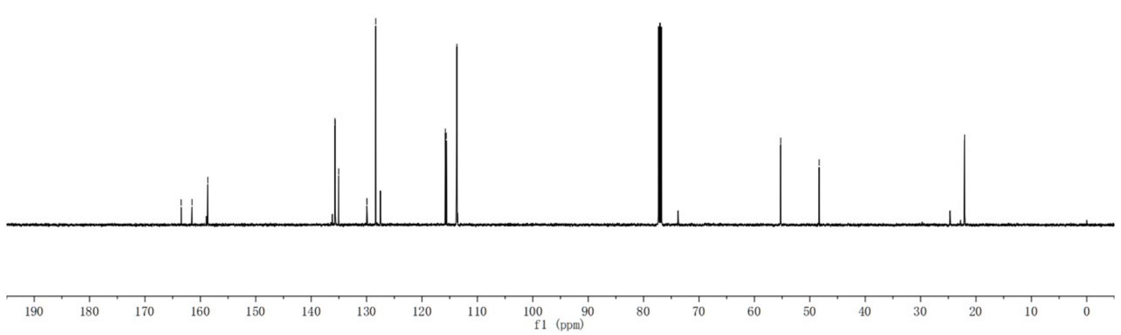

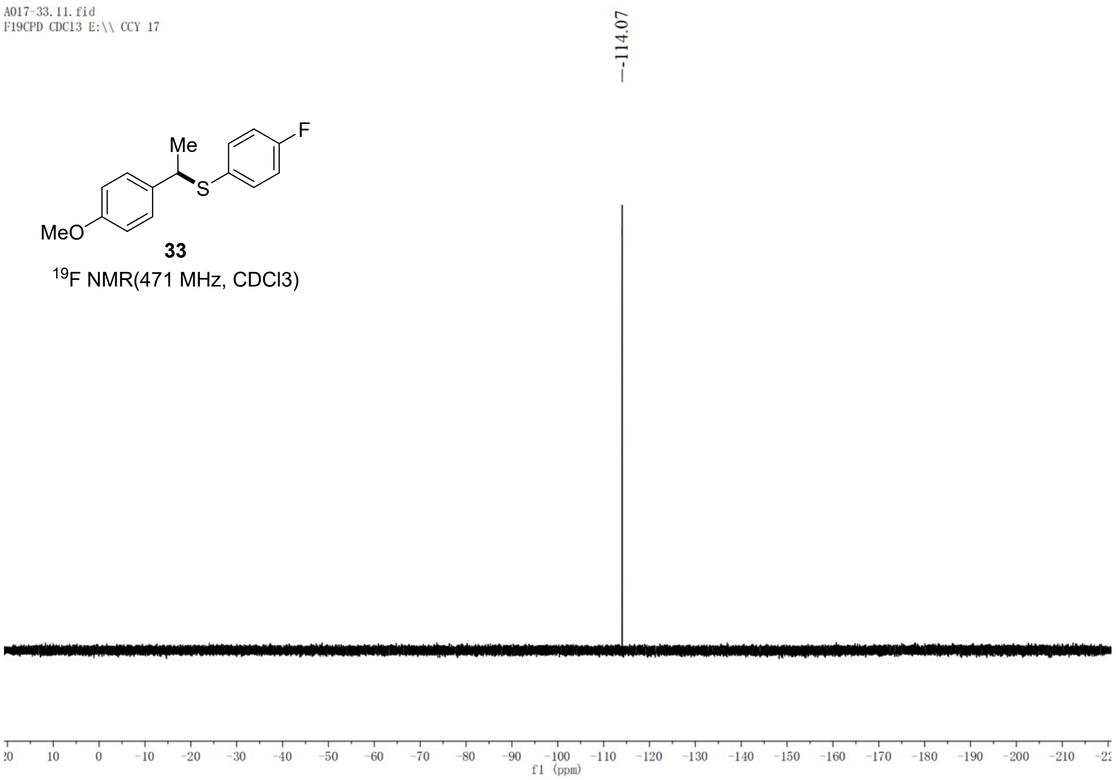
A017-33.11.fid
F19CPD CDCl3 E:\\ CCY 17
-114.07
Me
S
F
MeO
33
19F NMR(471 MHz, CDCl3)
f1 (ppm)

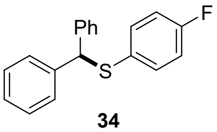

34

^{1}H NMR(500 MHz, $CDCl_3$)

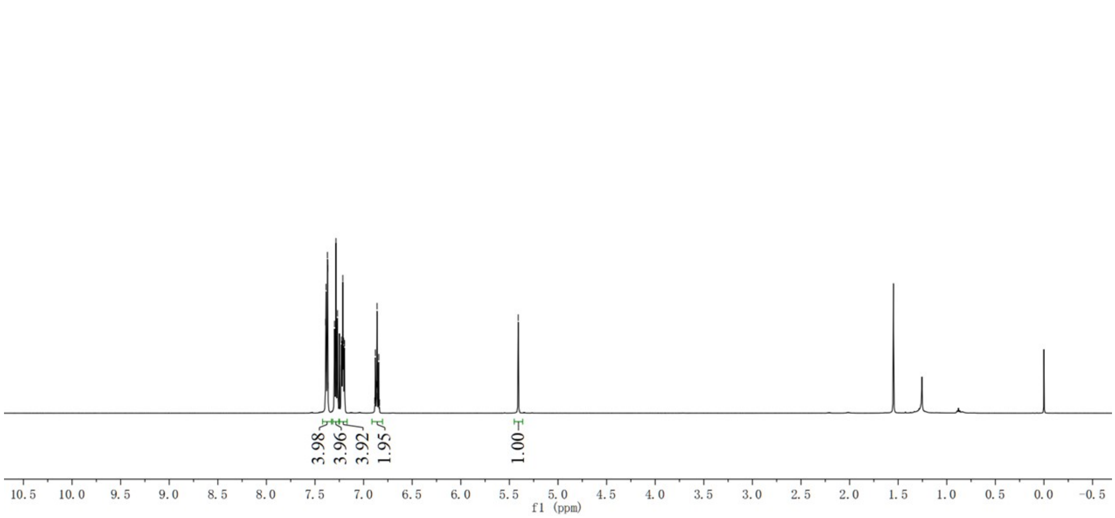

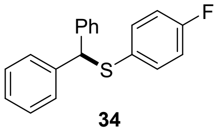

34

^{13}C NMR(126 MHz, $CDCl_3$)

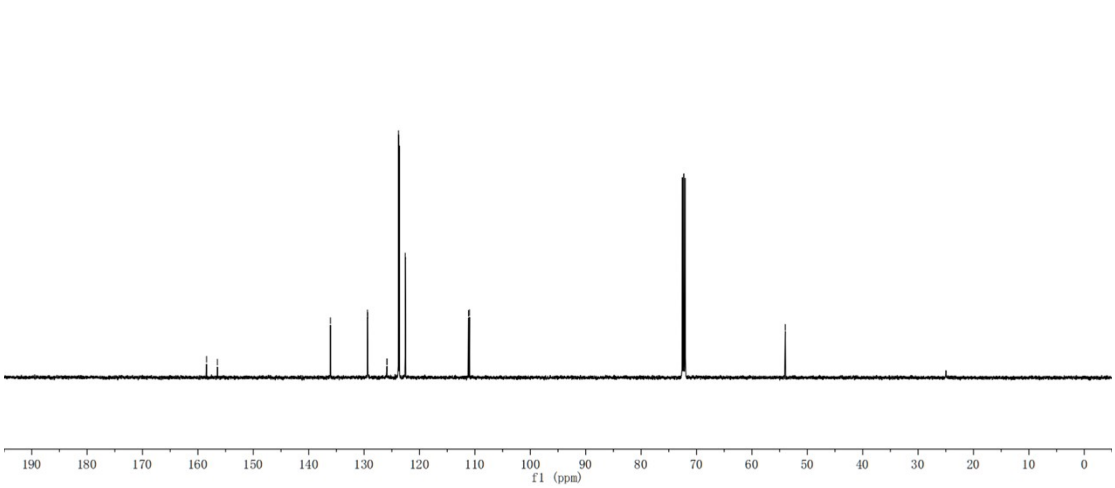

A017-34.11.fid
F19CPD CDCl3 E:\\ CCY 13

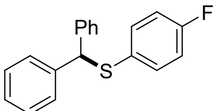

34

^{19}F NMR(471 MHz, CDCl3)

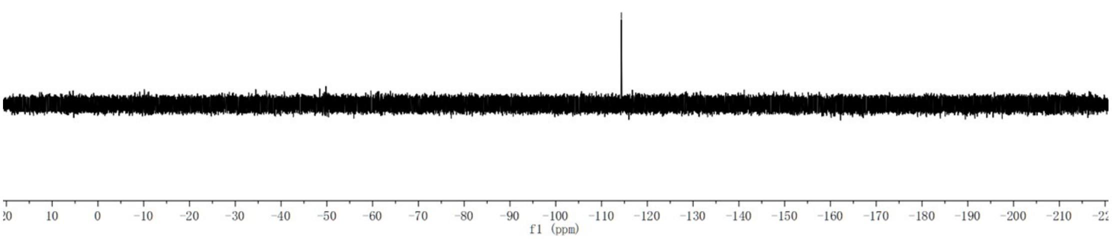

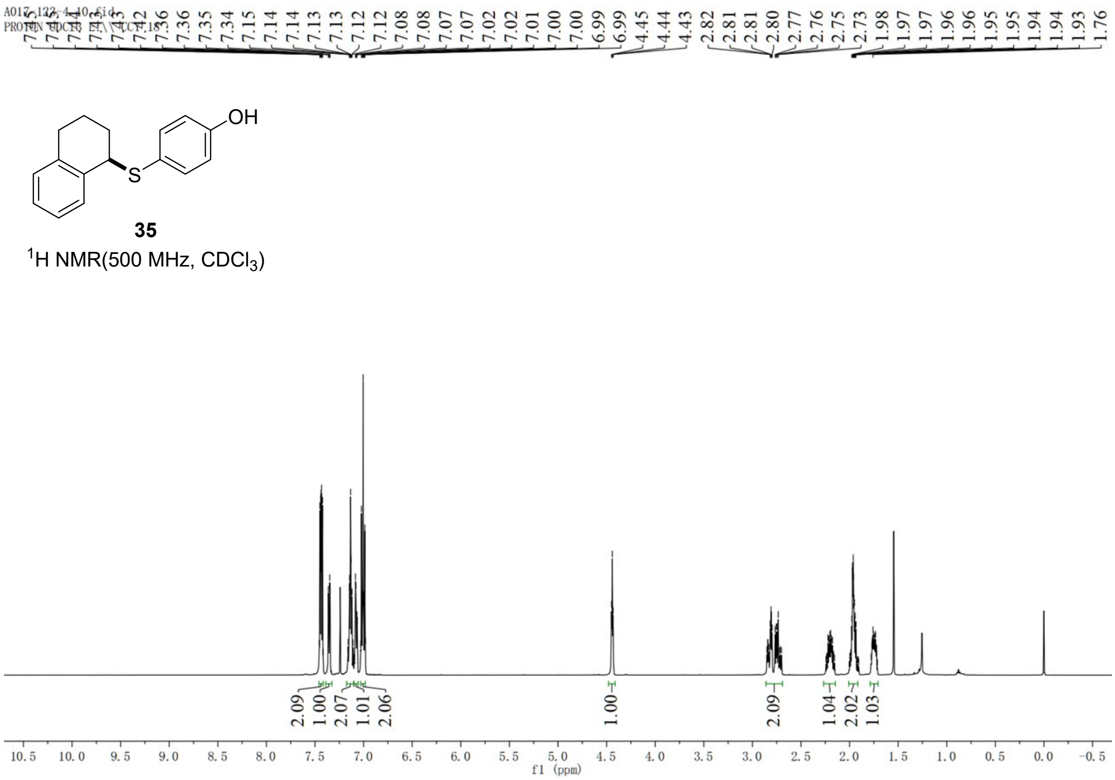

35

^{1}H NMR(500 MHz, $CDCl_3$)

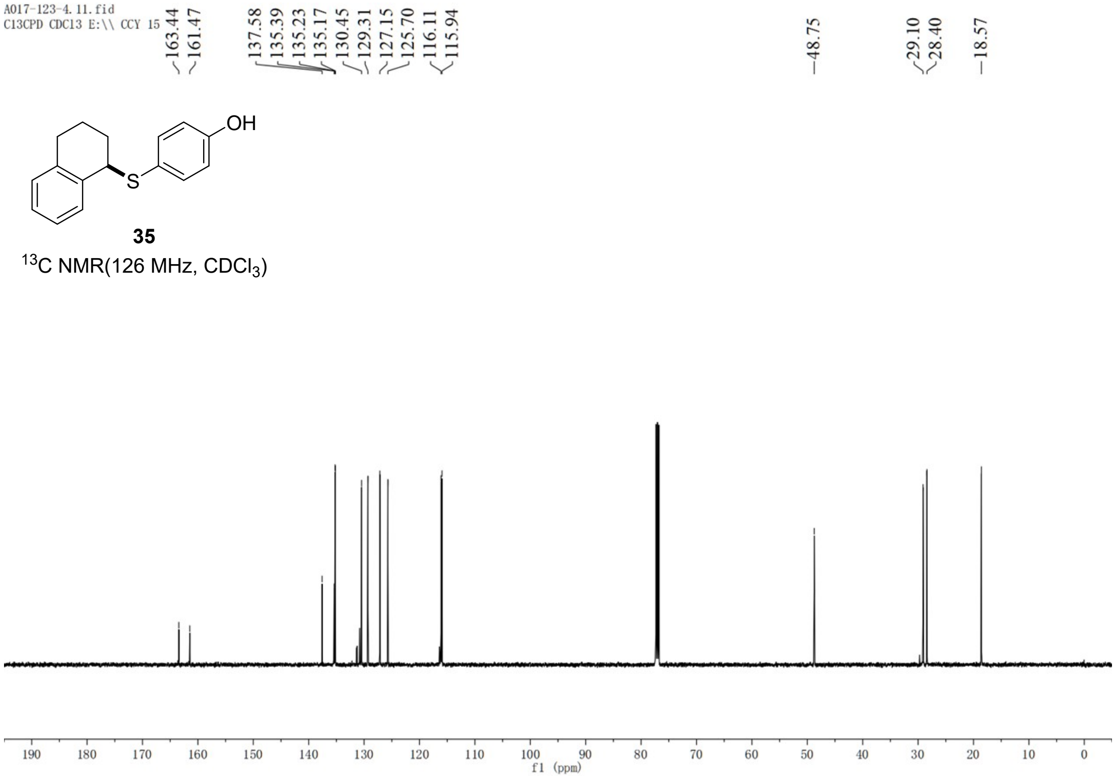

35

^{13}C NMR(126 MHz, $CDCl_3$)

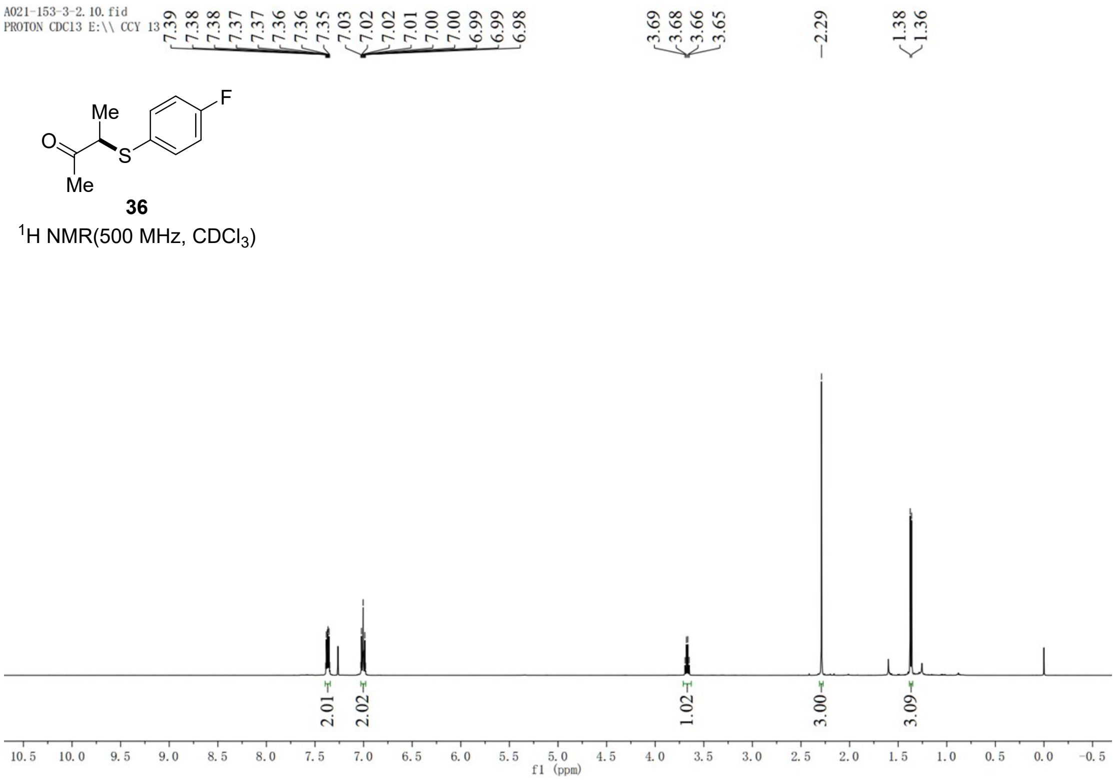
A021-153-3-2.10.fid
PROTON CDCl3 E:\\ CCY 13
Me
O
S
F
Me
36
1H NMR(500 MHz, CDCl3)
f1 (ppm)

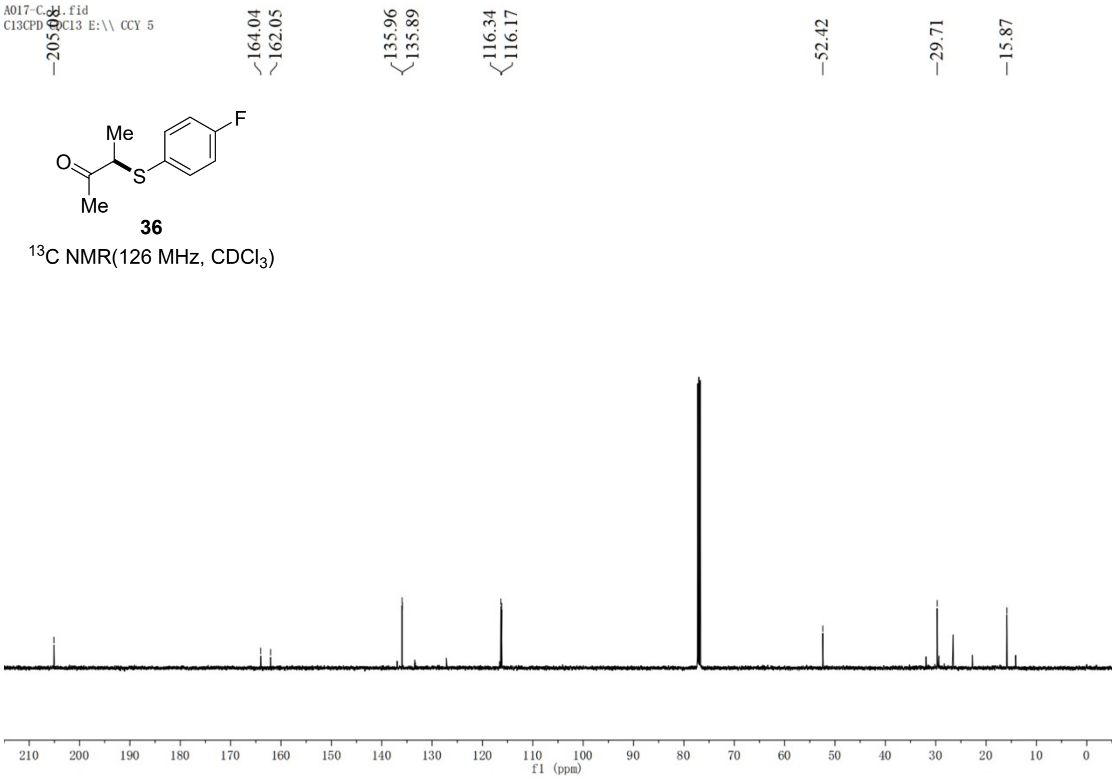
A017-C.1.fid
C13CPD CDCl3 E:\\ CCY 5
Me
O
S
F
Me
36
13C NMR(126 MHz, CDCl3)
f1 (ppm)

A017-36.11.fid
F19CPD CDCl3 E:\\ CCY 14

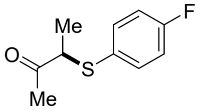

36

^{19}F NMR(471 MHz, CDCl3)

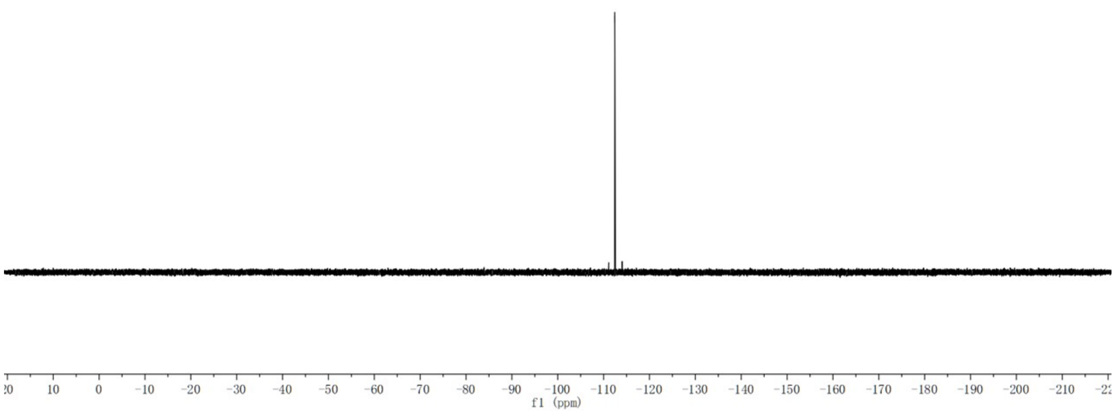

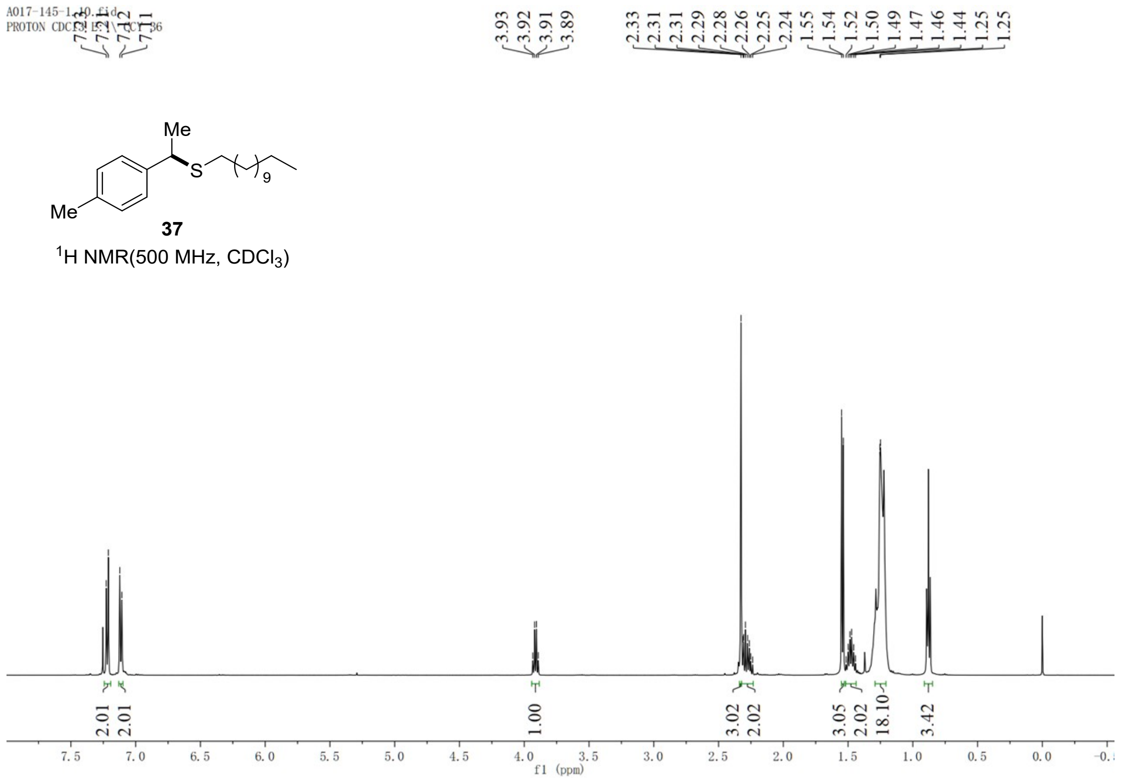
A017-145-1.10.fid
PROTON CDCl3 E:\\ CCY 36
7.23 7.21 7.12 7.11
3.93 3.92 3.91 3.89
2.33 2.31 2.31 2.29 2.28 2.26 2.25 2.24
1.55 1.54 1.52 1.50 1.49 1.47 1.46 1.44 1.25 1.25
Me
S
9
Me
37
1H NMR(500 MHz, CDCl3)
2.01 2.01 1.00 3.02 2.02 3.05 2.02 18.10 3.42
f1 (ppm)

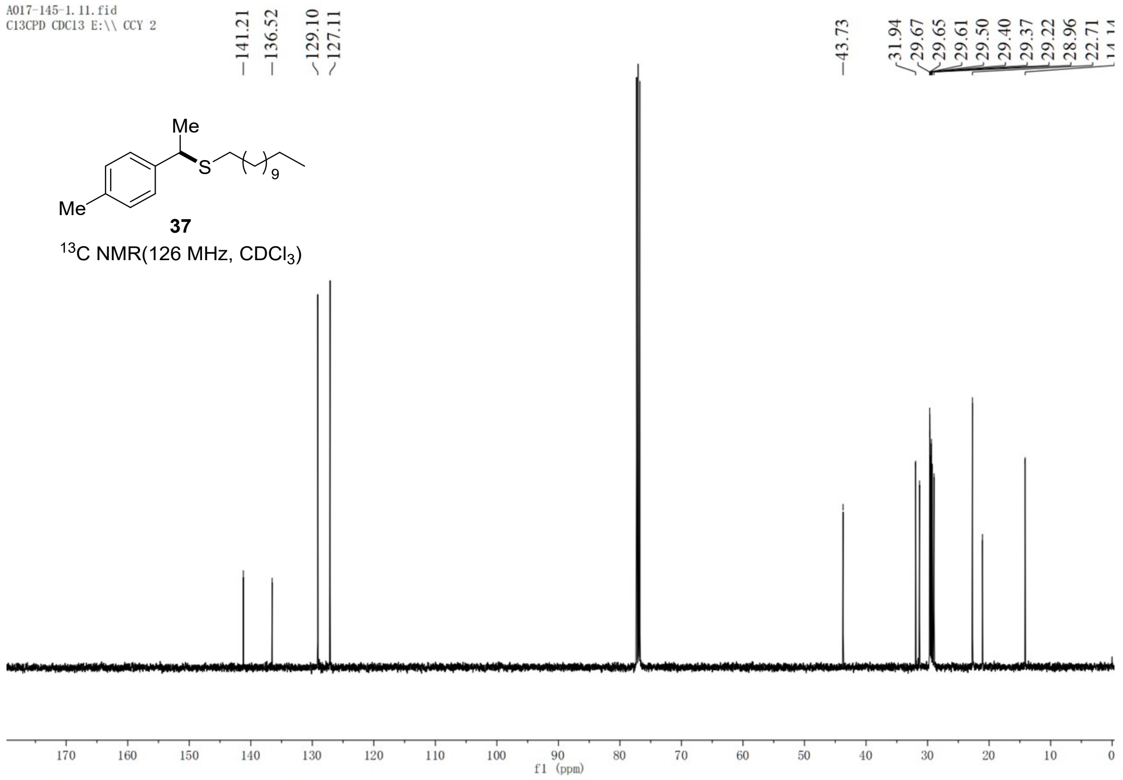
A017-145-1.11.fid
C13CPD CDCl3 E:\\ CCY 2
141.21 136.52 129.10 127.11
43.73
31.94 29.67 29.65 29.61 29.50 29.40 29.37 29.22 28.96 22.71
Me
S
9
Me
37
13C NMR(126 MHz, CDCl3)
f1 (ppm)

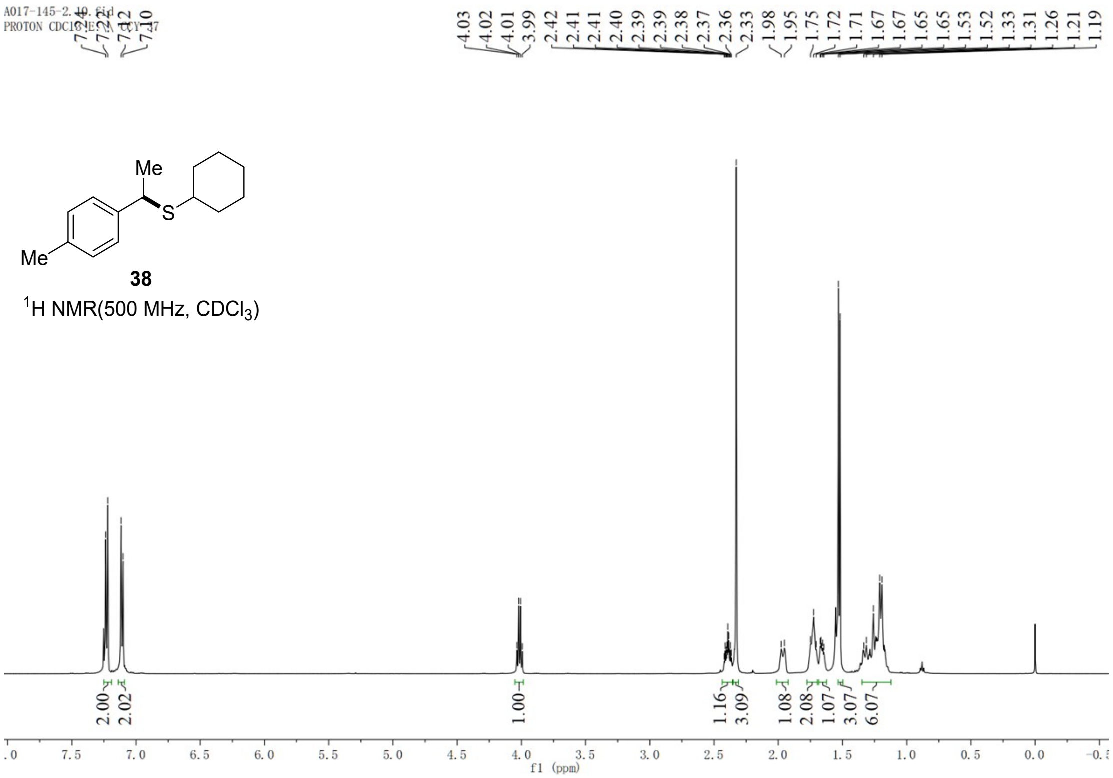
A017-145-2.10.fid
PROTON CDCl3 E:\\ CCY 47
7.24 7.22 7.12 7.10
4.03 4.02 4.01 3.99 2.42 2.41 2.41 2.40 2.39 2.39 2.38 2.37 2.36 2.33 1.98 1.95 1.75 1.72 1.71 1.67 1.67 1.65 1.65 1.53 1.52 1.33 1.31 1.26 1.21 1.19
Me
S
Me
38
^{1}H NMR(500 MHz, CDCl$_3$)
2.00 2.02 1.00 1.16 3.09 1.08 2.08 1.07 3.07 6.07
f1 (ppm)

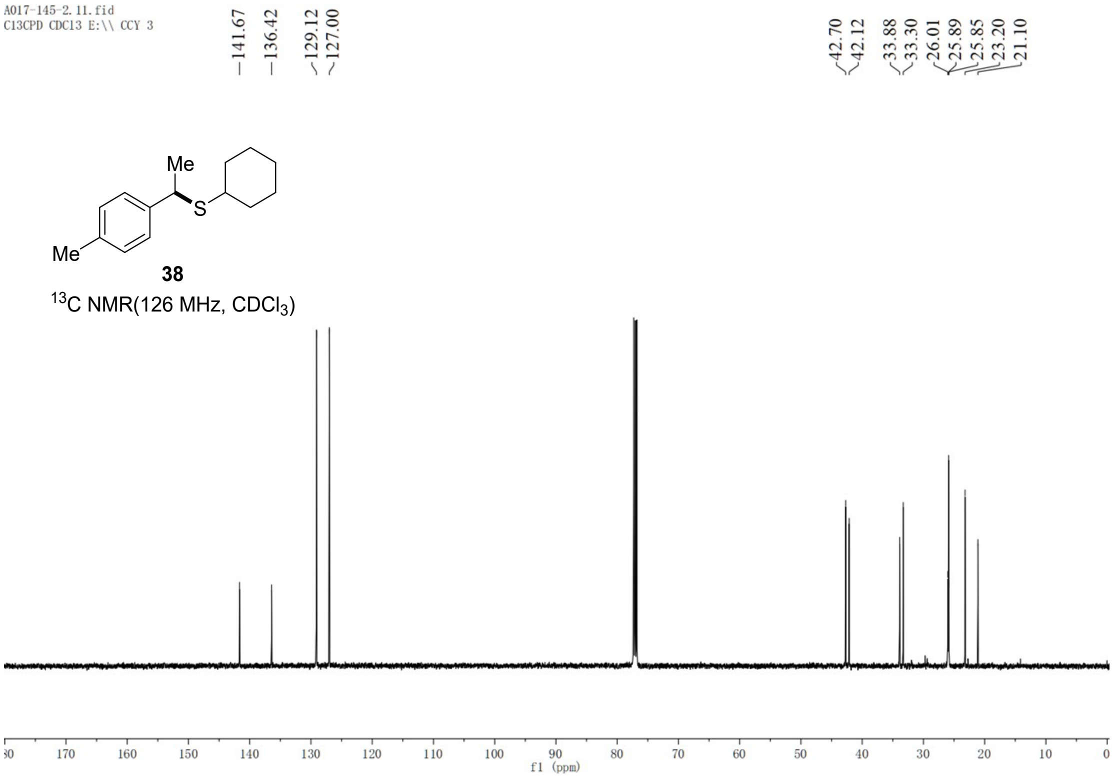
A017-145-2.11.fid
C13CPD CDCl3 E:\\ CCY 3
141.67 136.42 129.12 127.00
42.70 42.12 33.88 33.30 26.01 25.89 25.85 23.20 21.10
Me
S
Me
38
^{13}C NMR(126 MHz, CDCl$_3$)
f1 (ppm)

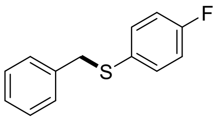

39

^{1}H NMR(500 MHz, $CDCl_3$)

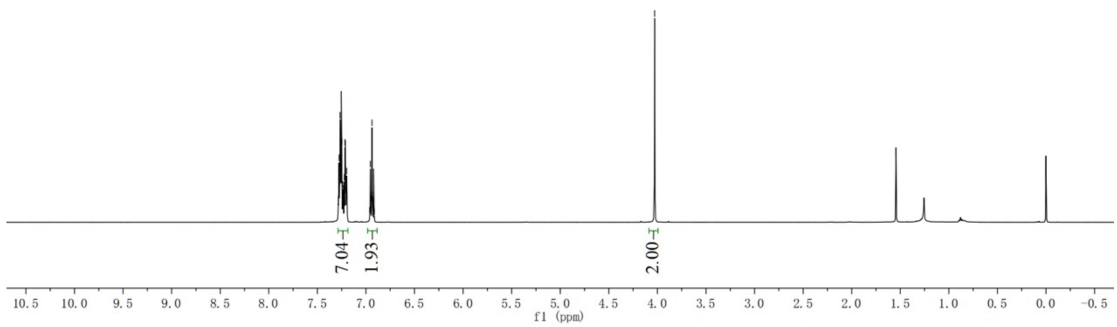

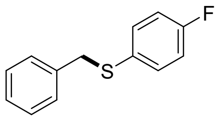

39

^{13}C NMR(126 MHz, $CDCl_3$)

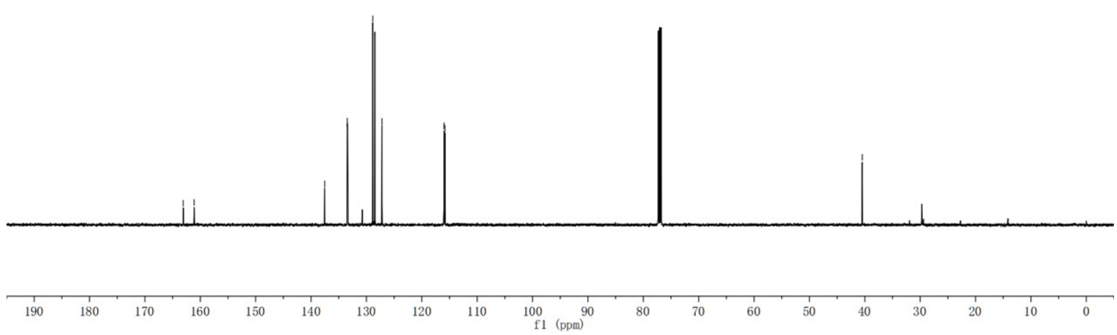

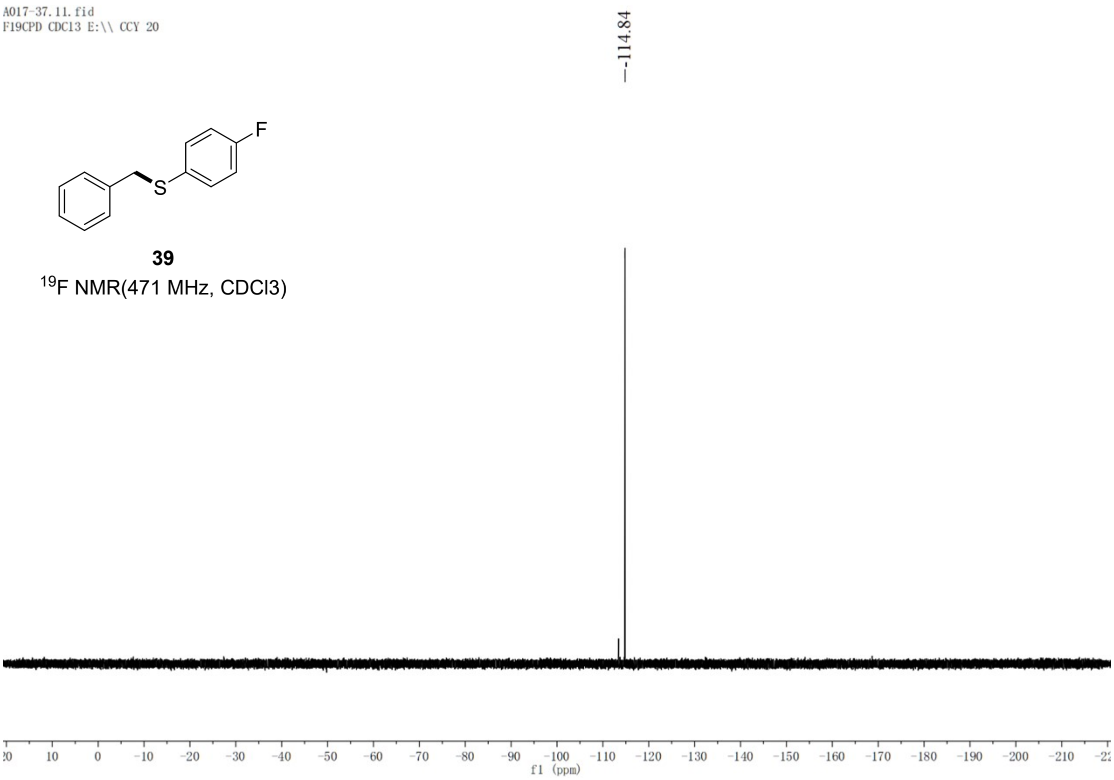

39

^{19}F NMR(471 MHz, CDCl3)

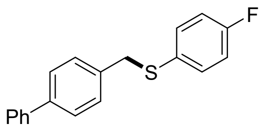

40

^1H NMR(500 MHz, $CDCl_3$)

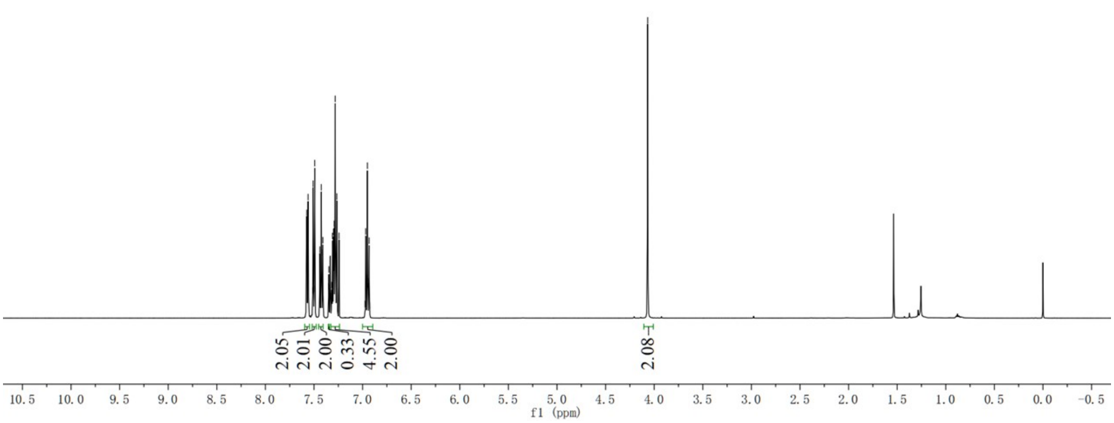

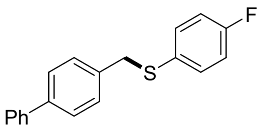

40

^{13}C NMR(126 MHz, $CDCl_3$)

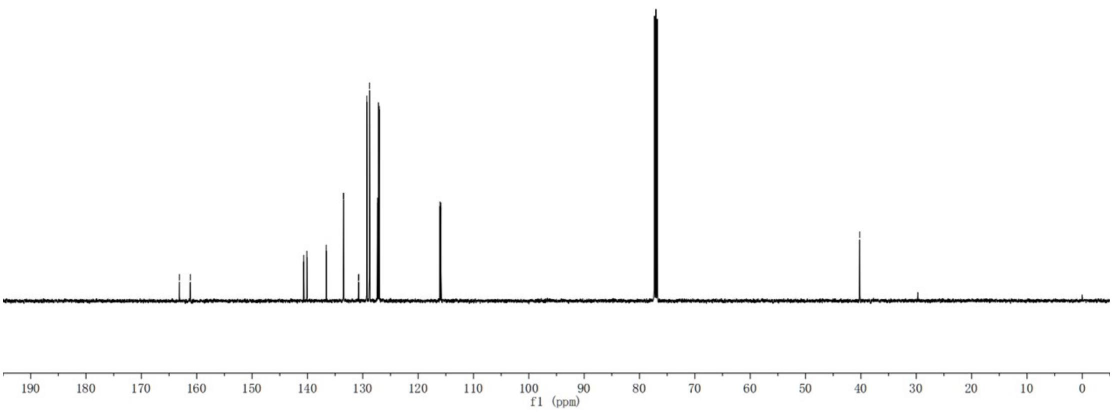

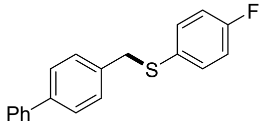

40

^{19}F NMR(471 MHz, CDCl3)

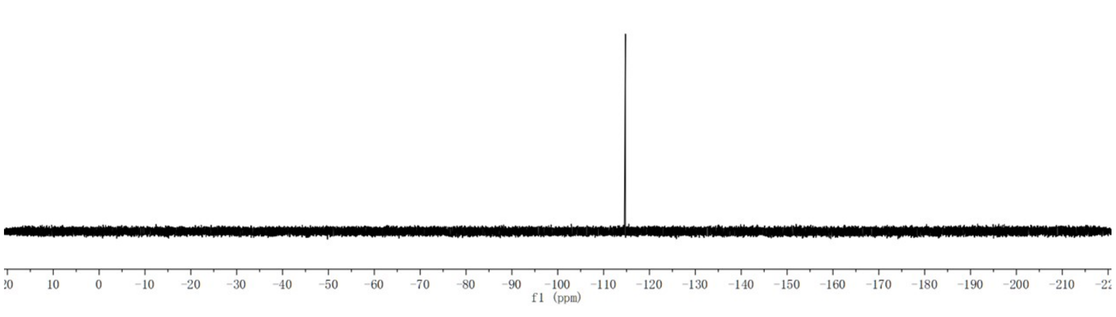

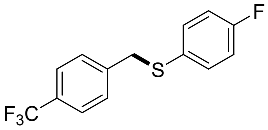

41

^{1}H NMR(500 MHz, $CDCl_3$)

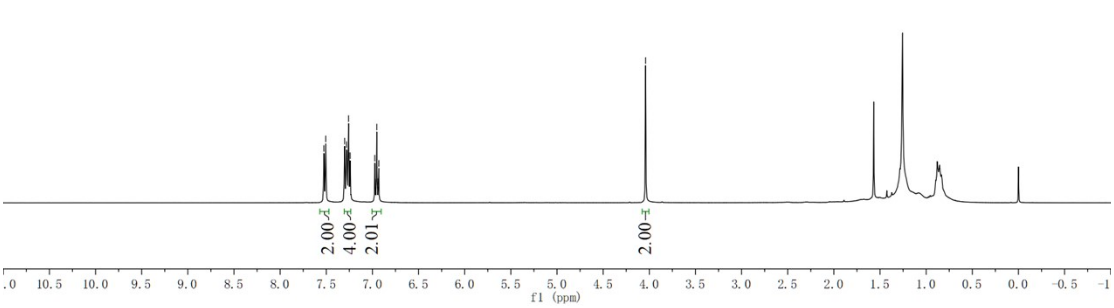

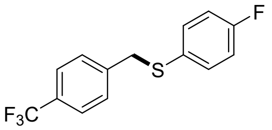

41

^{13}C NMR(126 MHz, $CDCl_3$)

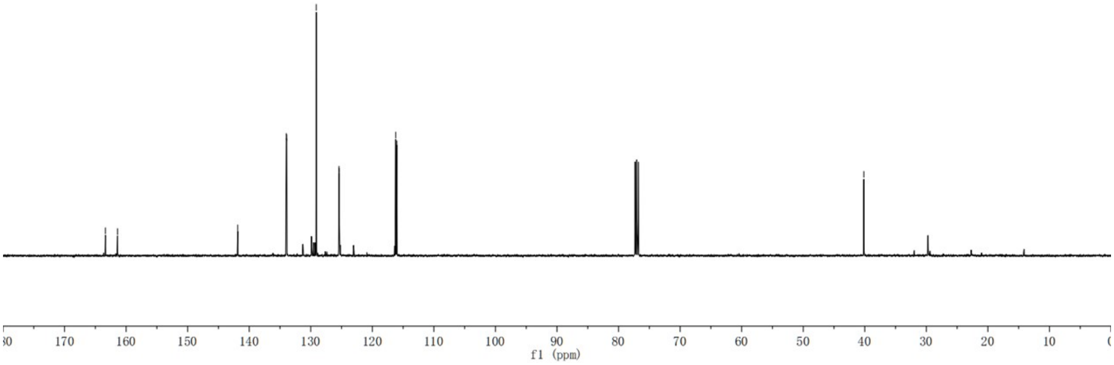

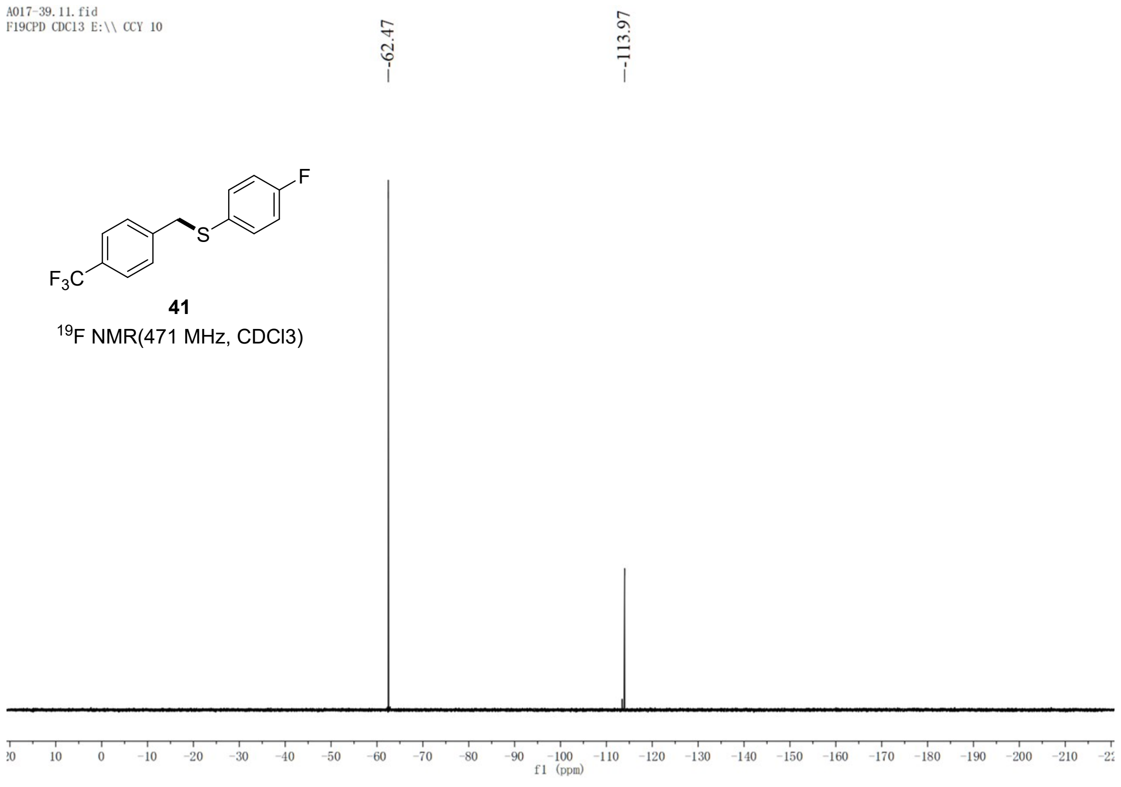
A017-39.11.fid
F19CPD CDCl3 E:\\ CCY 10
-62.47
-113.97
F
S
F3C
41
19F NMR(471 MHz, CDCl3)
f1 (ppm)

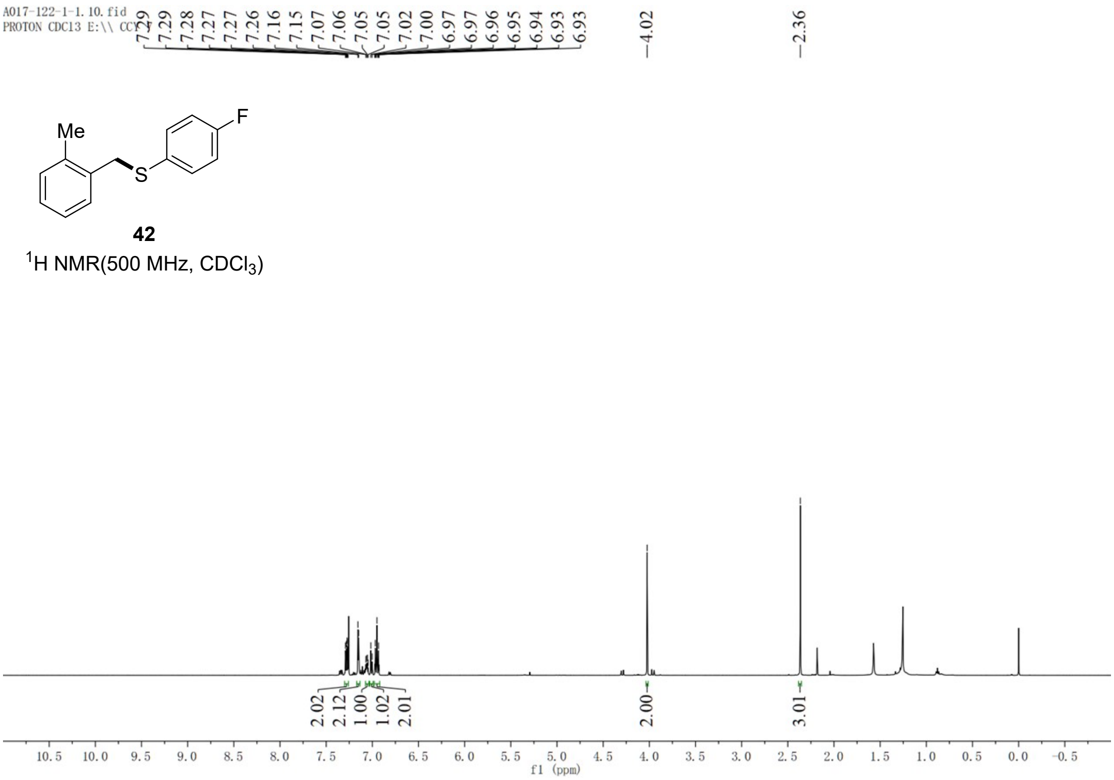

42

^{1}H NMR(500 MHz, $CDCl_3$)

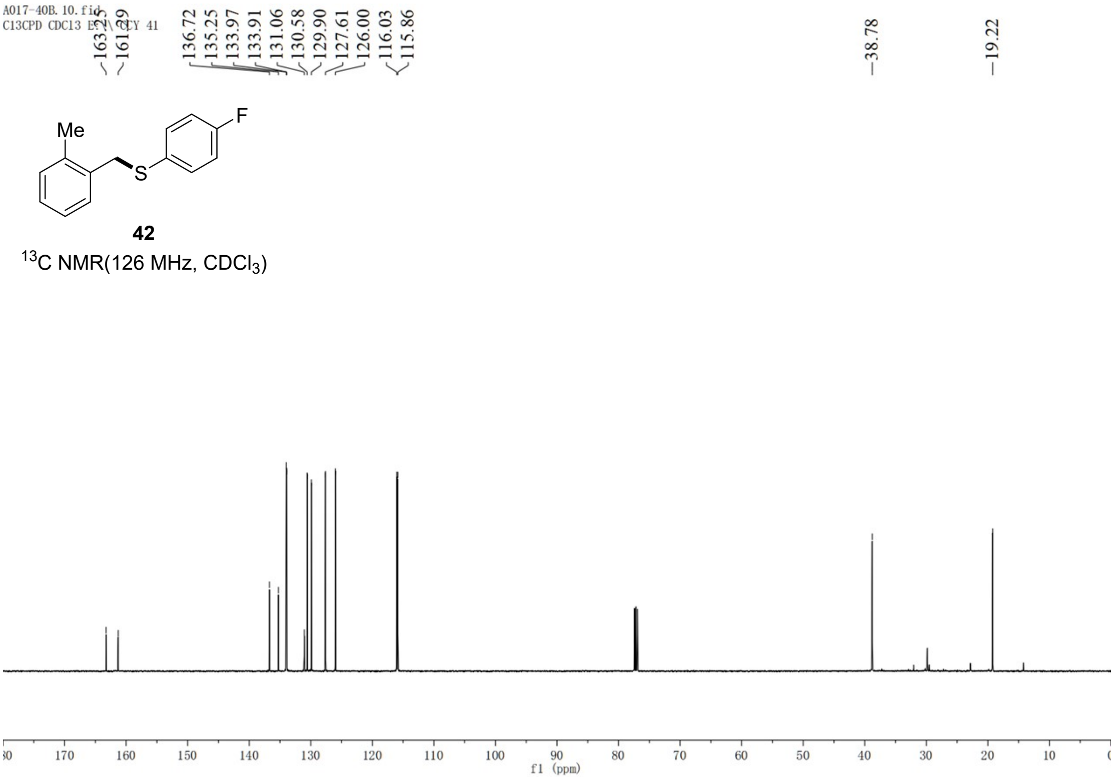

42

^{13}C NMR(126 MHz, $CDCl_3$)

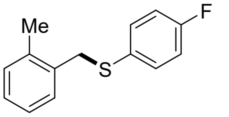

42

^{19}F NMR(471 MHz, $CDCl_3$)

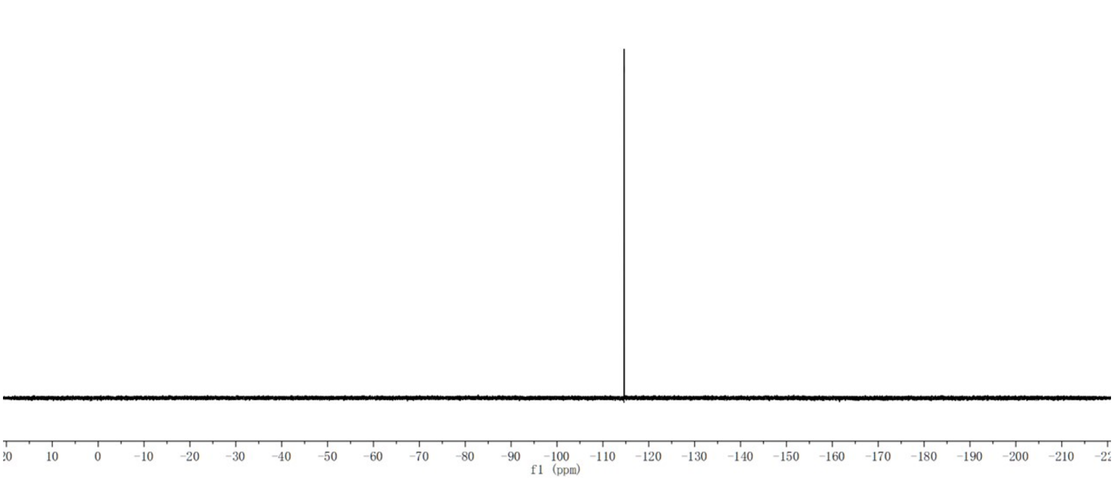

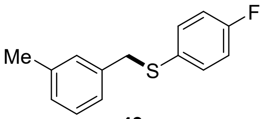

43

^1H NMR(500 MHz, $CDCl_3$)

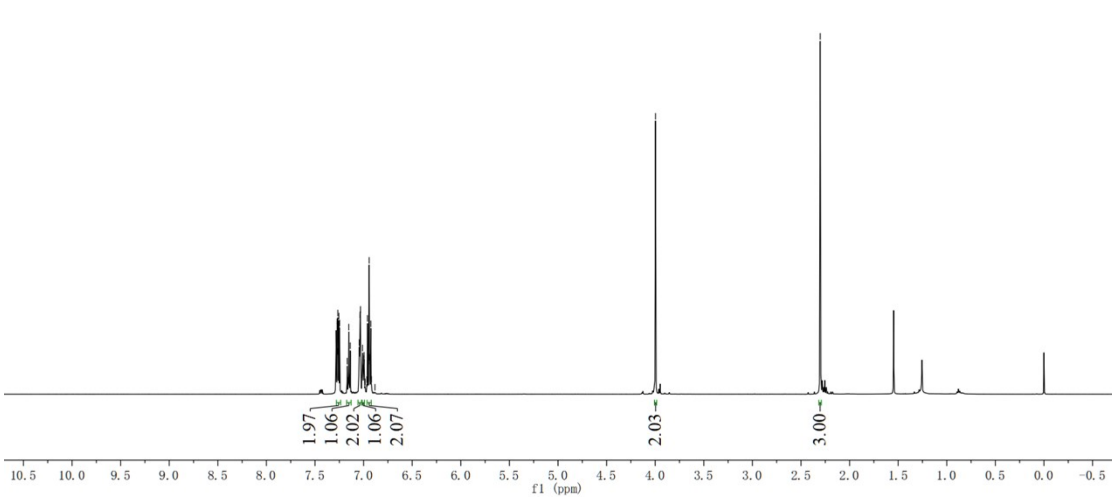

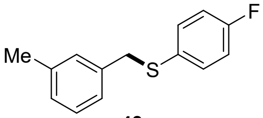

43

^{13}C NMR(126 MHz, $CDCl_3$)

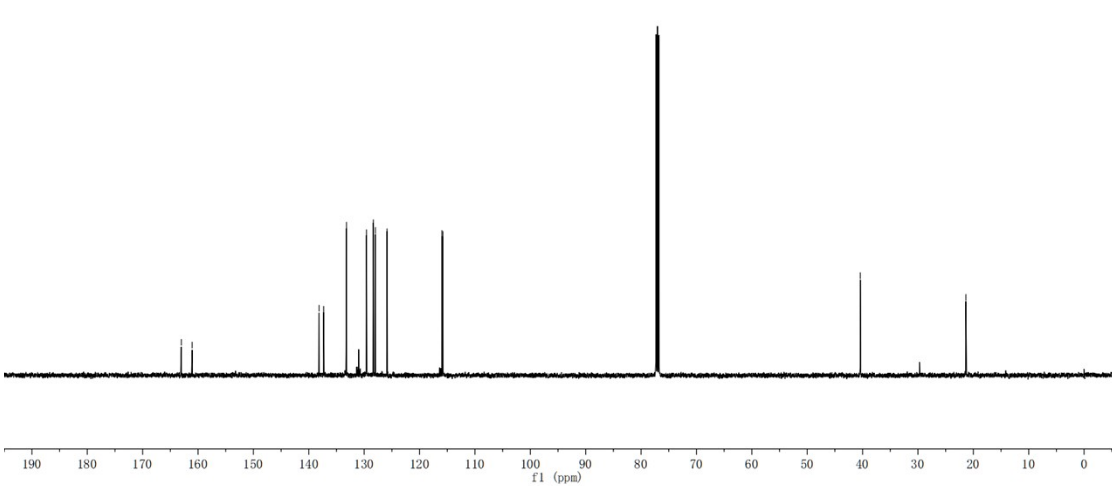

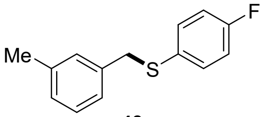

43

^{19}F NMR(471 MHz, CDCl3)

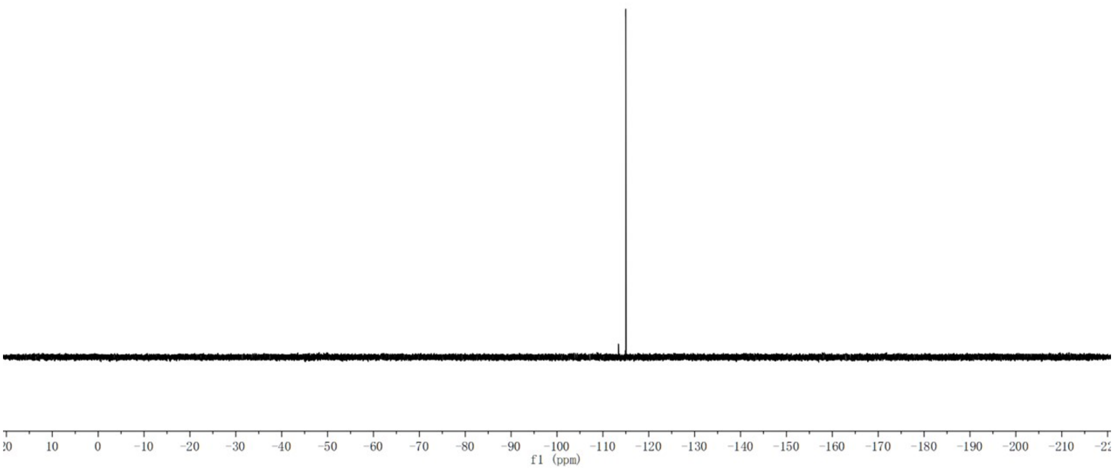

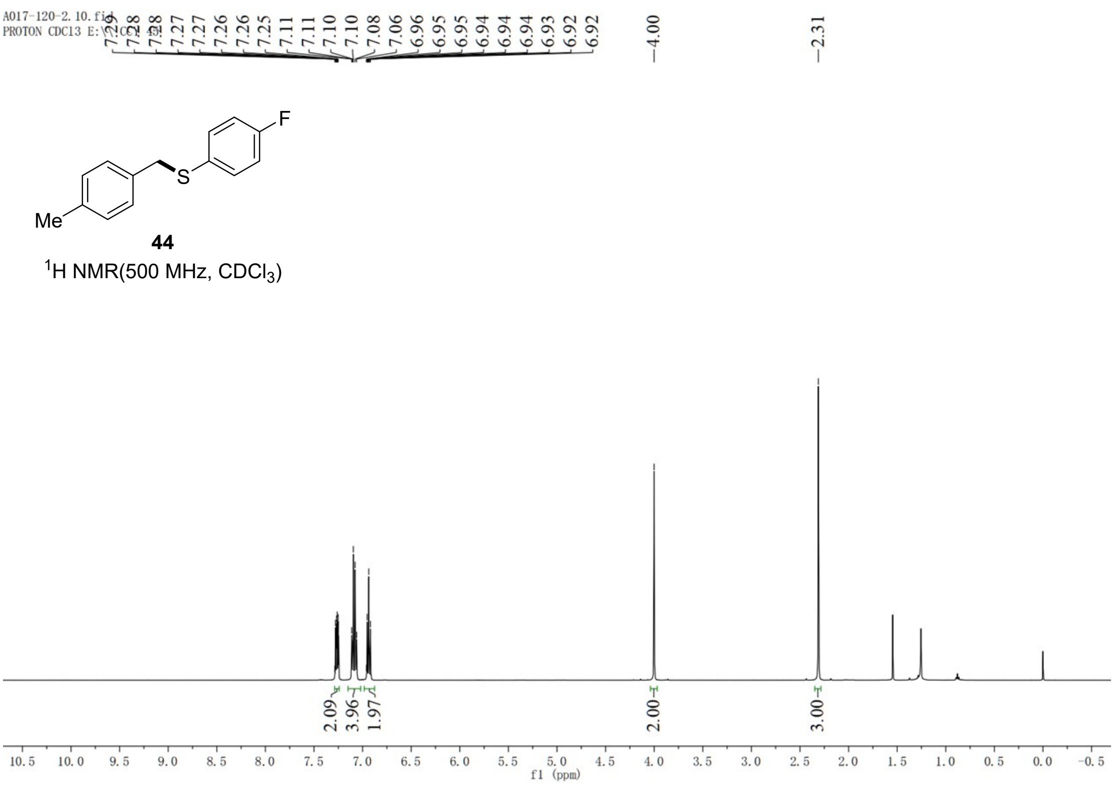
A017-120-2.10.fid
PROTON CDCl3
7.29 7.28 7.28 7.27 7.27 7.26 7.26 7.25 7.11 7.11 7.10 7.10 7.08 7.06 6.96 6.95 6.95 6.94 6.94 6.94 6.93 6.92 6.92
4.00
2.31
F
S
Me
44
^{1}H NMR(500 MHz, $CDCl_3$)
2.09 3.96 1.97
2.00
3.00
f1 (ppm)

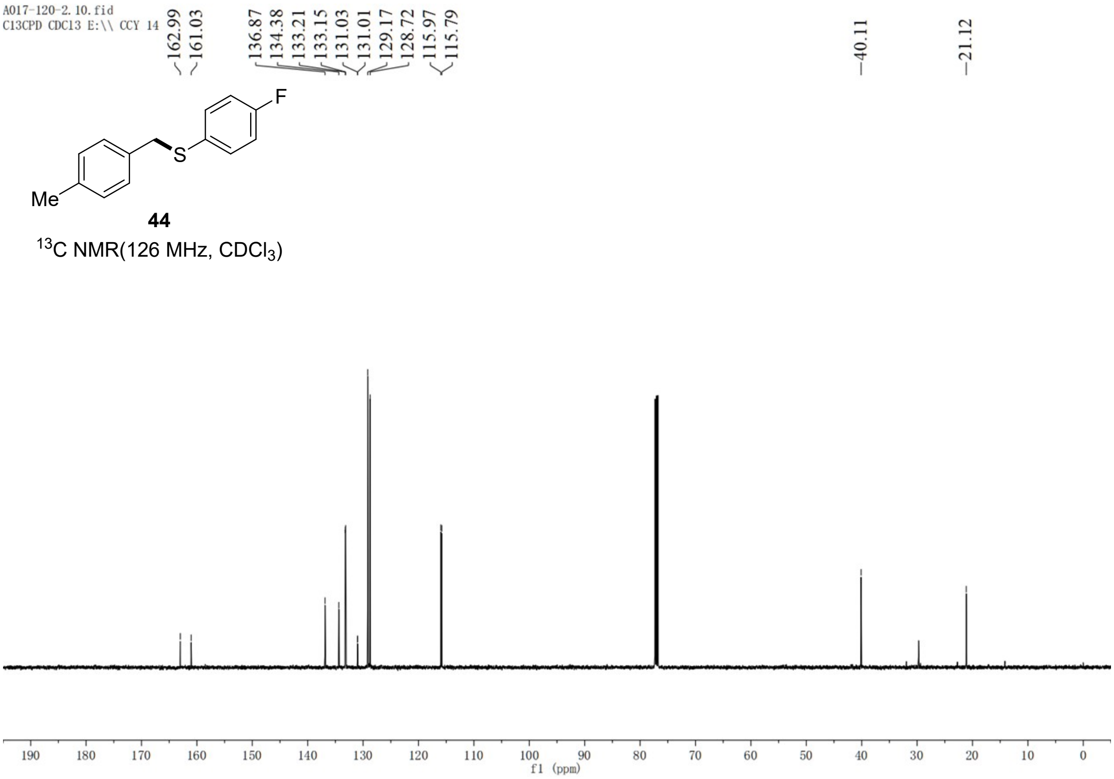
A017-120-2.10.fid
C13CPD CDCl3 E:\\ CCY 14
162.99 161.03
136.87 134.38 133.21 133.15 131.03 131.01 129.17 128.72 115.97 115.79
40.11
21.12
F
S
Me
44
^{13}C NMR(126 MHz, $CDCl_3$)
f1 (ppm)

A017-426.11.fid
F19CPD CDCl3 E:\\ CCY 2

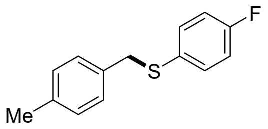

44

^{19}F NMR(471 MHz, $CDCl_3$)

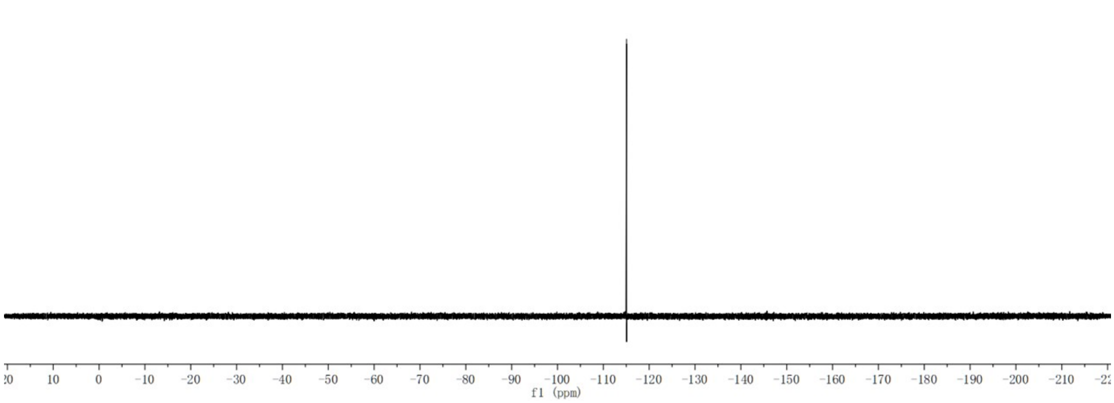

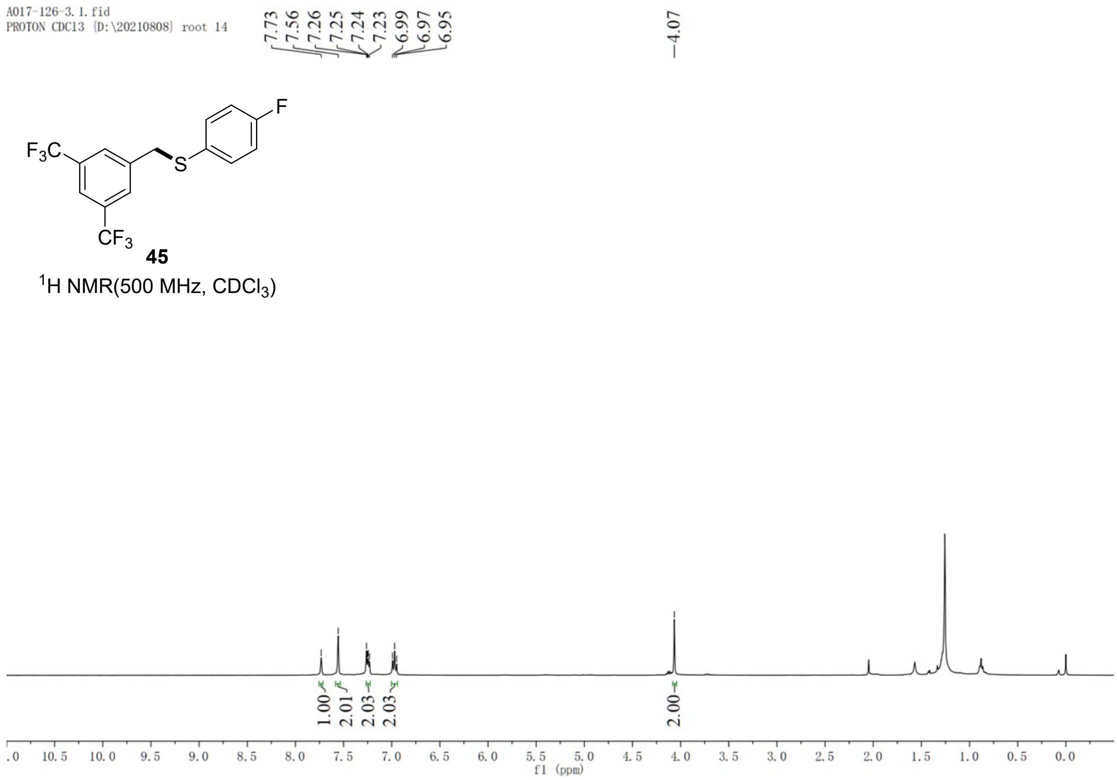

45

^{1}H NMR(500 MHz, $CDCl_3$)

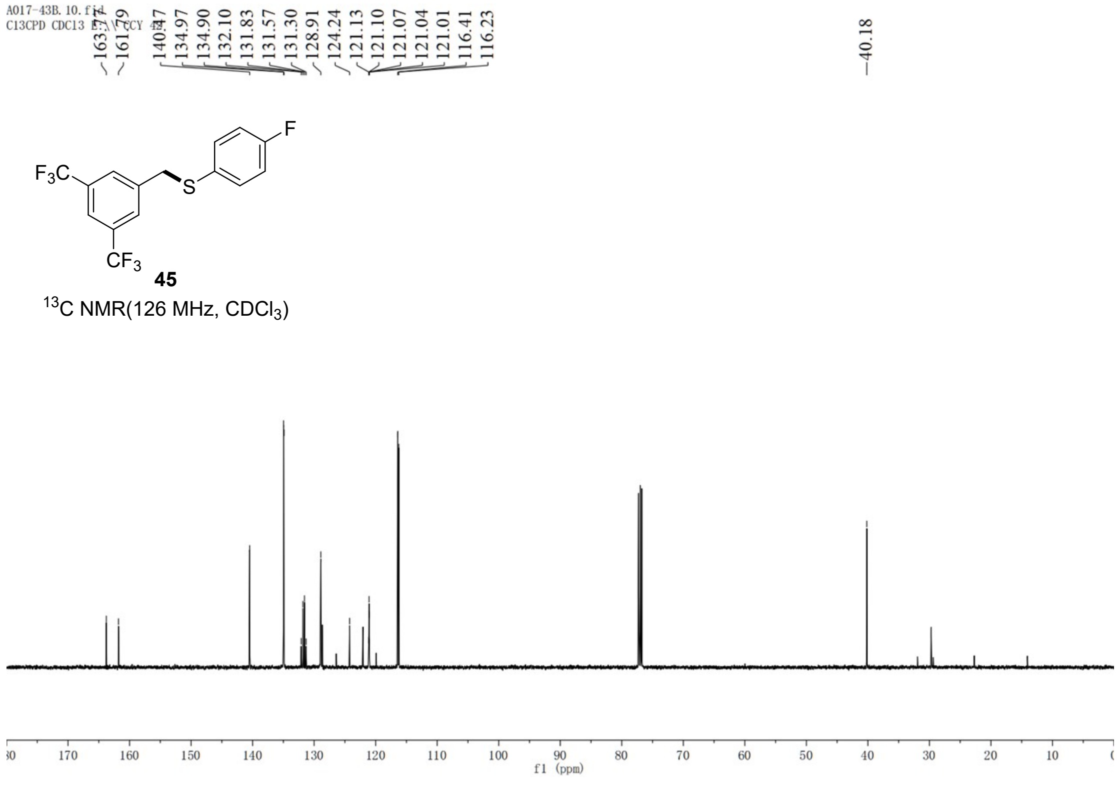

45

^{13}C NMR(126 MHz, $CDCl_3$)

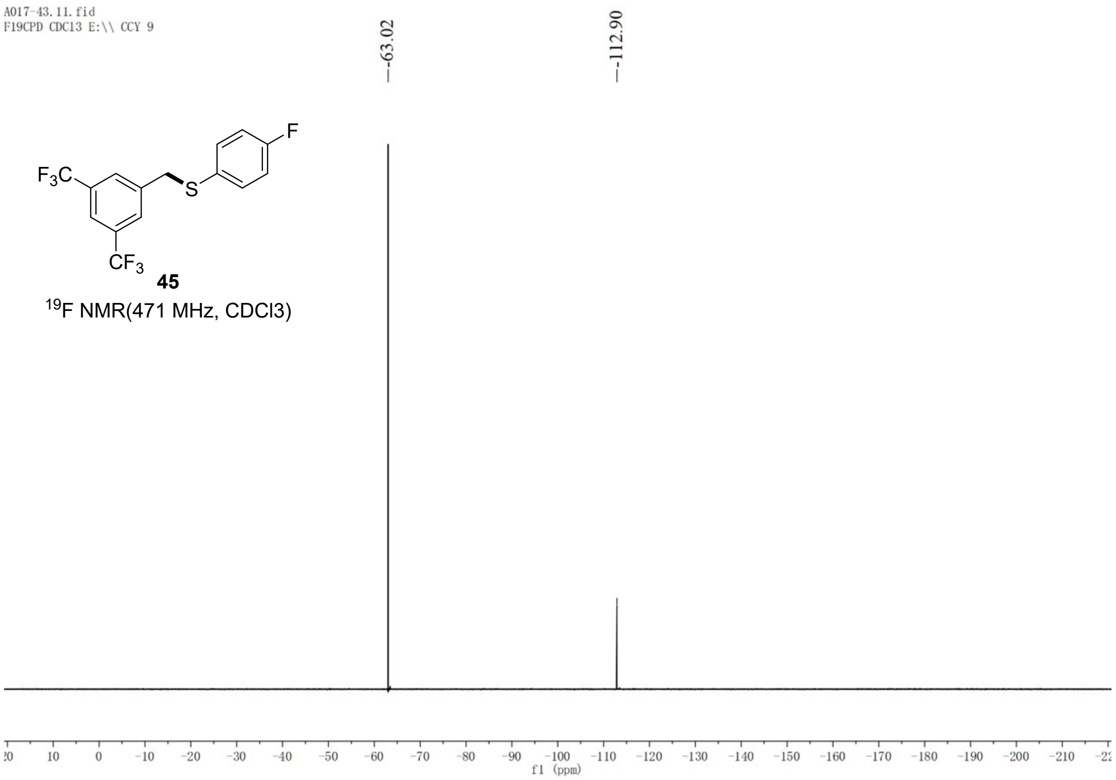
A017-43.11.fid
F19CPD CDCl3 E:\\ CCY 9
-63.02
-112.90
F3C
S
F
CF3
45
19F NMR(471 MHz, CDCl3)
f1 (ppm)

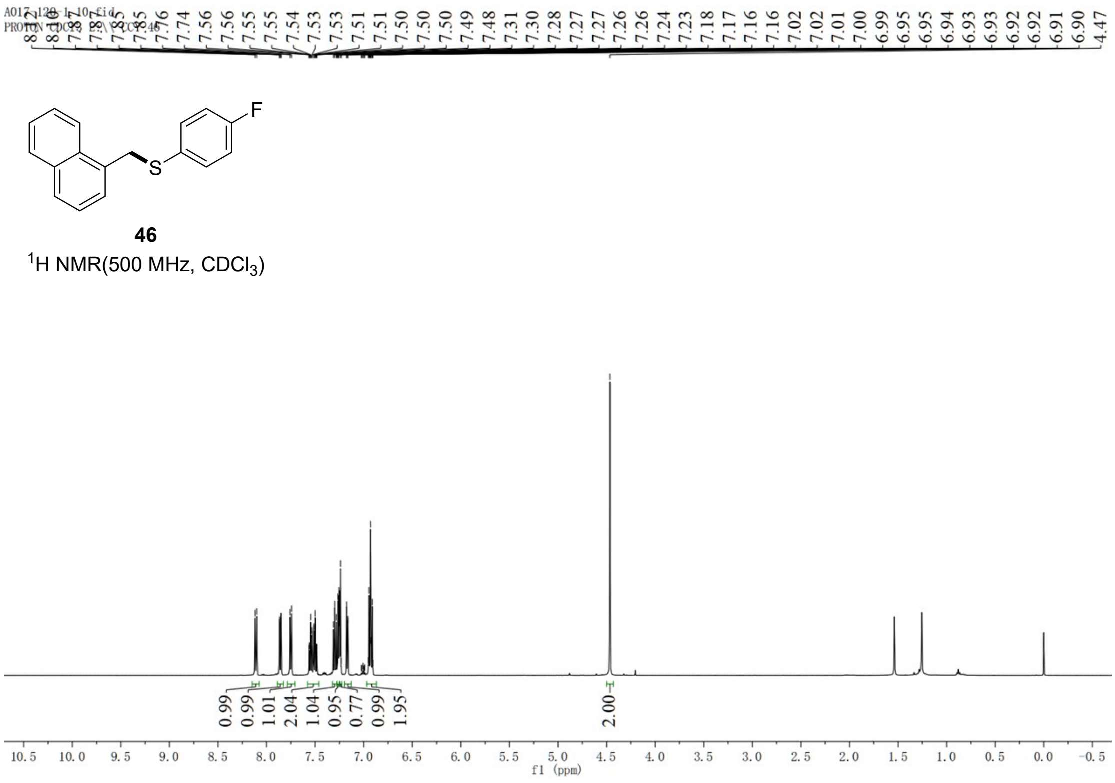

46

^{1}H NMR(500 MHz, $CDCl_3$)

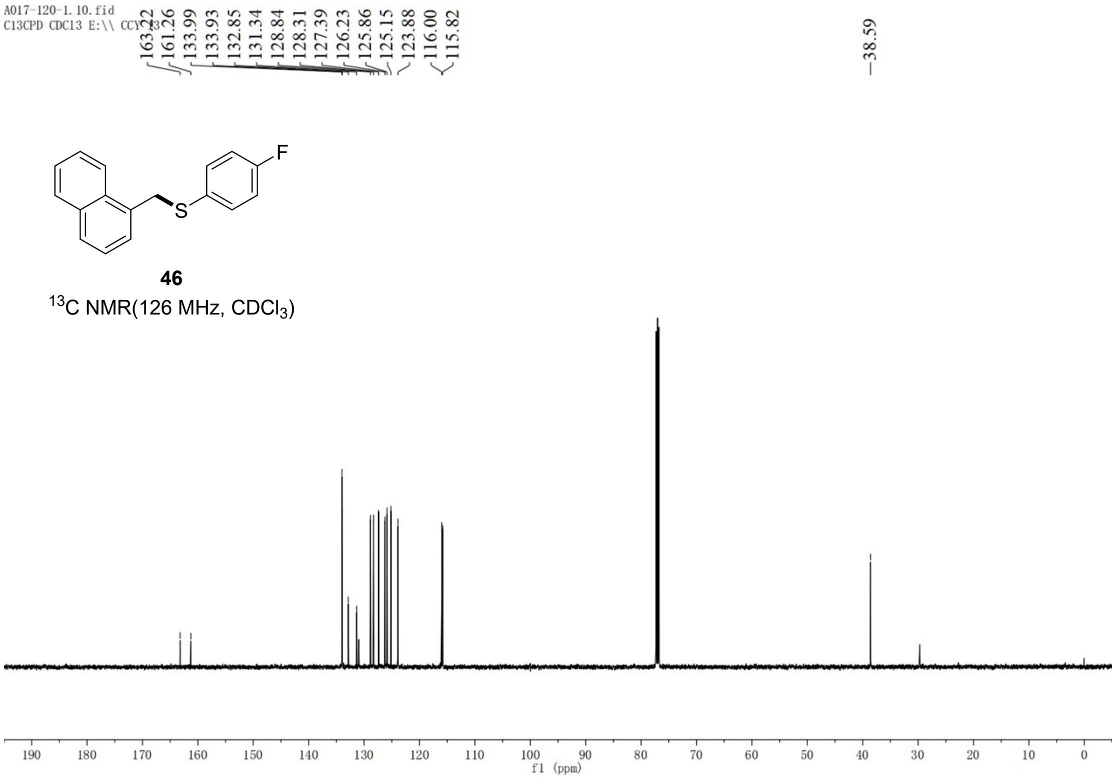

46

^{13}C NMR(126 MHz, $CDCl_3$)

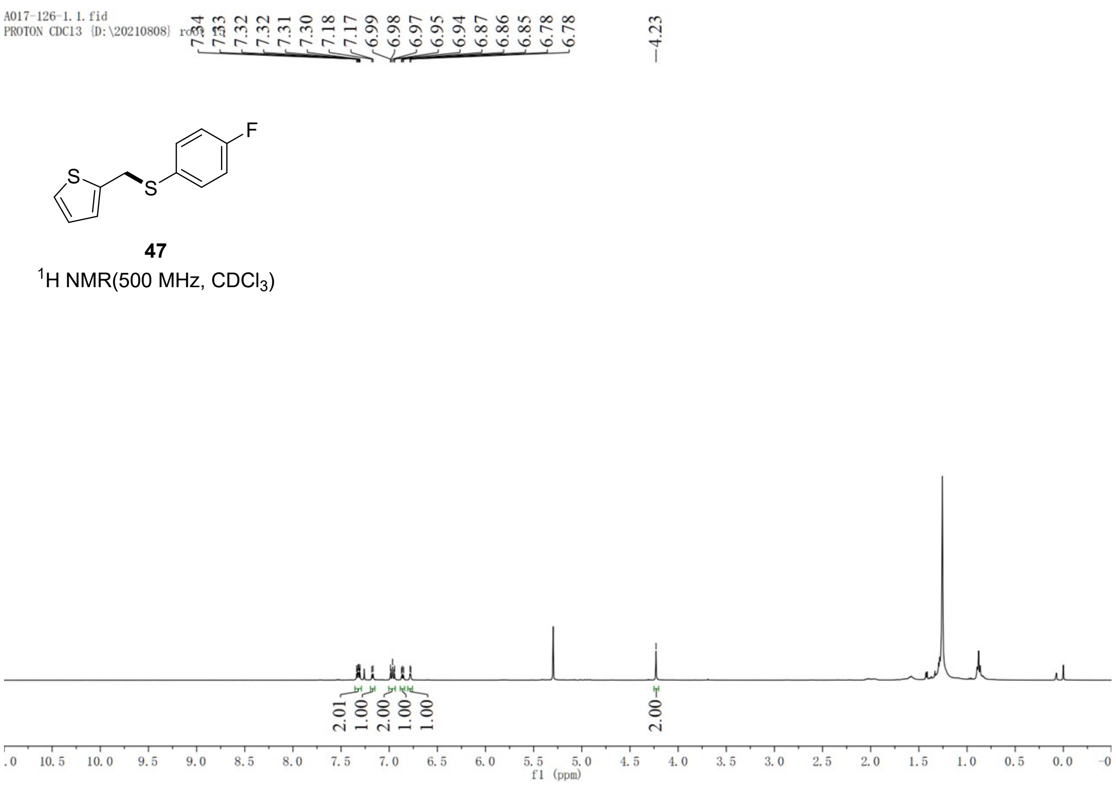

47

^{1}H NMR(500 MHz, $CDCl_3$)

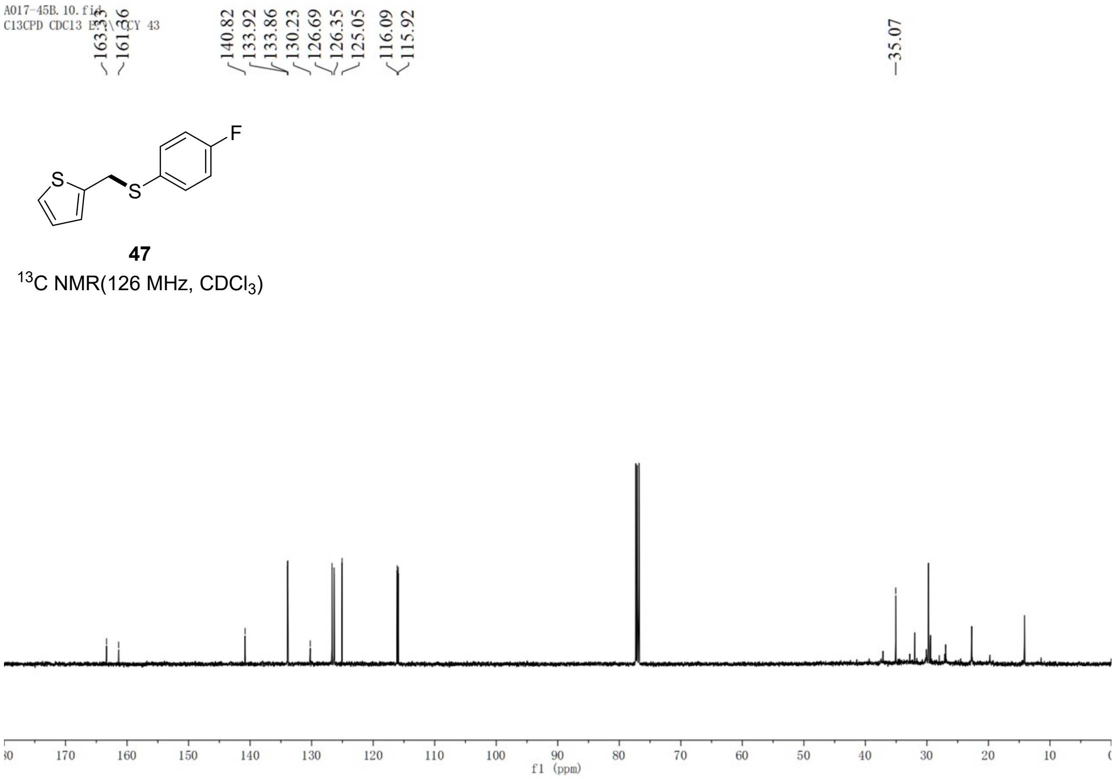

47

^{13}C NMR(126 MHz, $CDCl_3$)

A017-45.11.fid
F19CPD CDCl3 E:\\ CCY 7

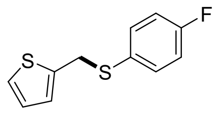

47

^{19}F NMR(471 MHz, CDCl3)

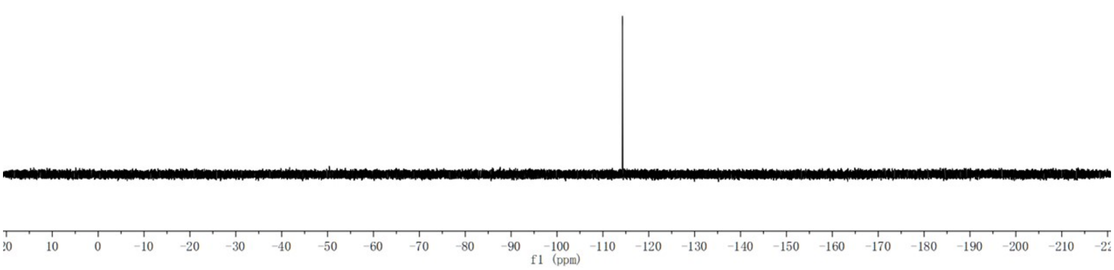

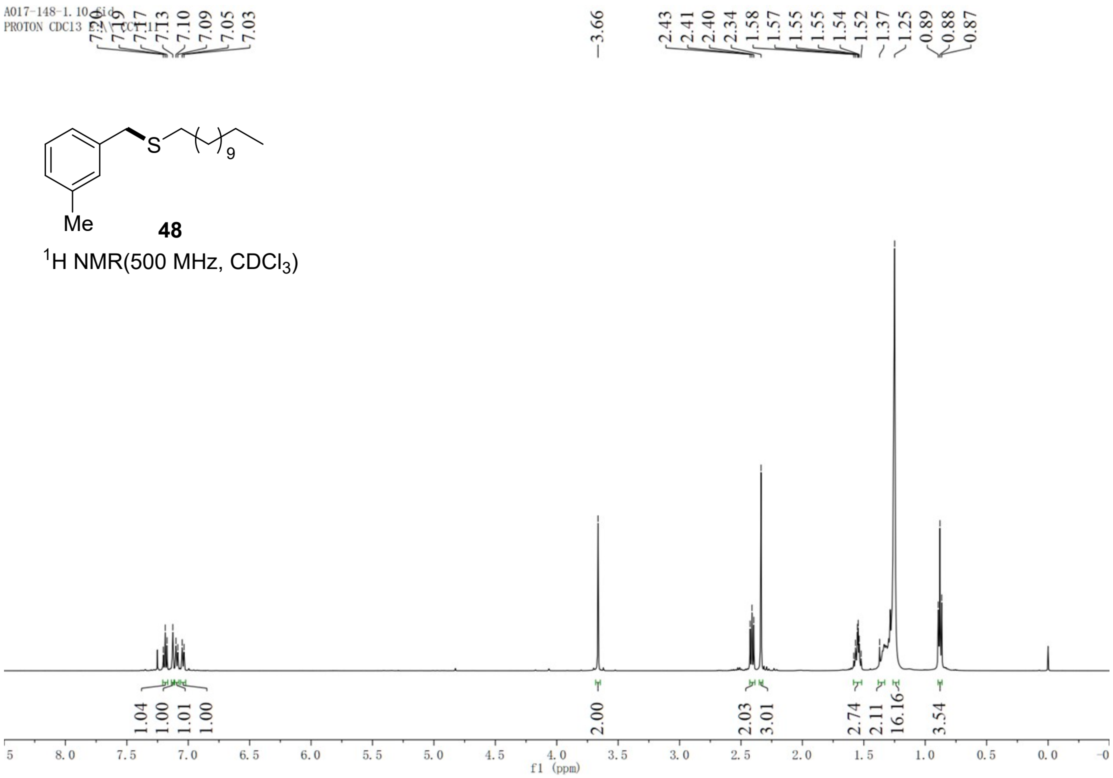
A017-148-1.10.fid
PROTON CDCl3 E:\\ CCY 11
7.20 7.19 7.17 7.13 7.10 7.09 7.05 7.03
3.66
2.43 2.41 2.40 2.34 1.58 1.57 1.55 1.55 1.54 1.52 1.37 1.25 0.89 0.88 0.87
S
9
Me
48
^{1}H NMR(500 MHz, $CDCl_3$)
1.04 1.00 1.01 1.00
2.00
2.03 3.01
2.74 2.11 16.16
3.54
f1 (ppm)

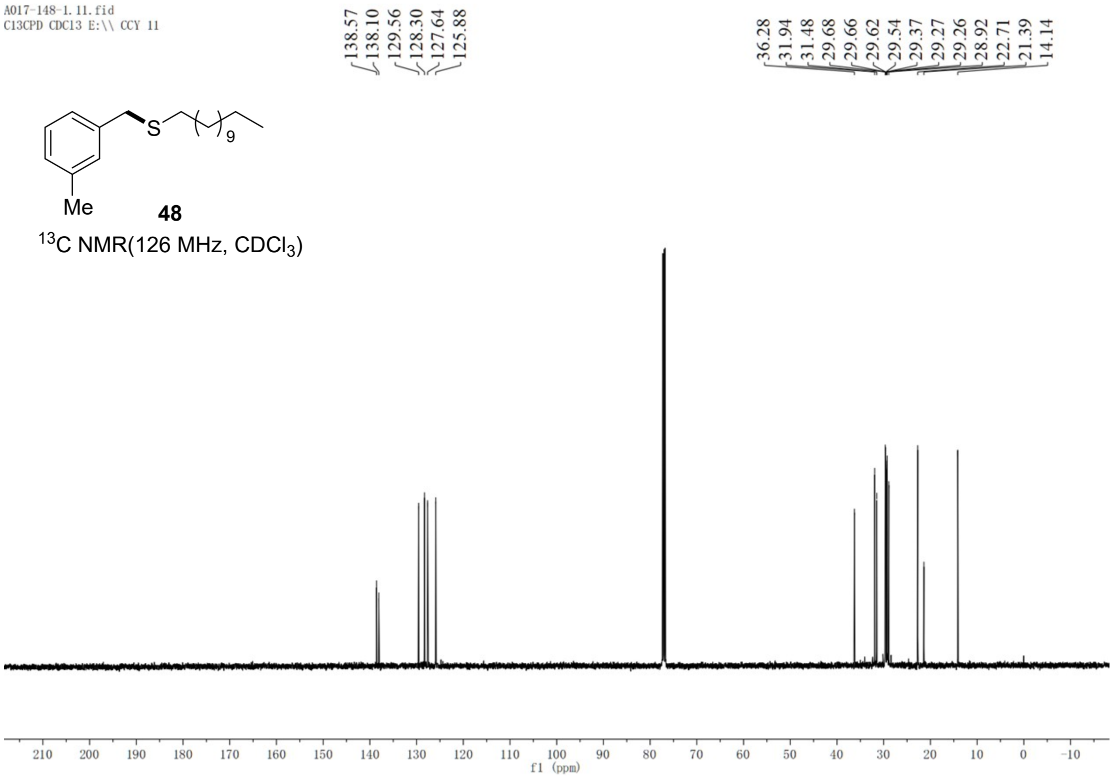
A017-148-1.11.fid
C13CPD CDCl3 E:\\ CCY 11
138.57 138.10 129.56 128.30 127.64 125.88
36.28 31.94 31.48 29.68 29.66 29.62 29.54 29.37 29.27 29.26 28.92 22.71 21.39 14.14
S
9
Me
48
^{13}C NMR(126 MHz, $CDCl_3$)
f1 (ppm)

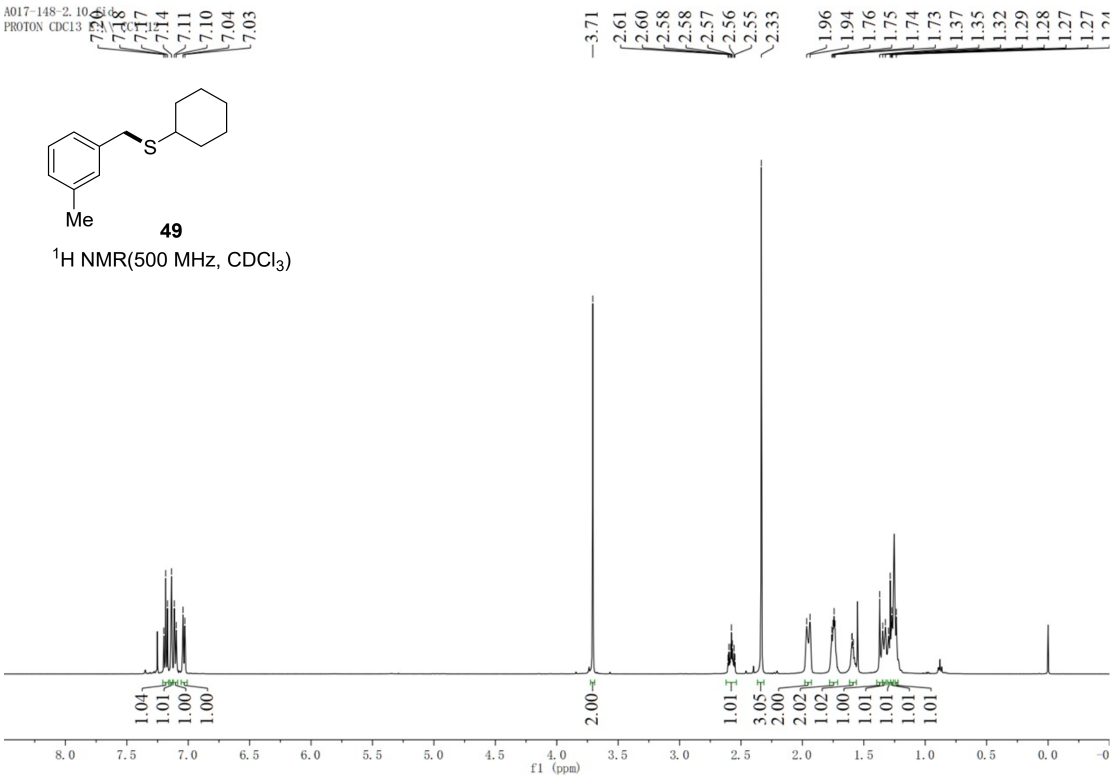

49

^{1}H NMR(500 MHz, $CDCl_3$)

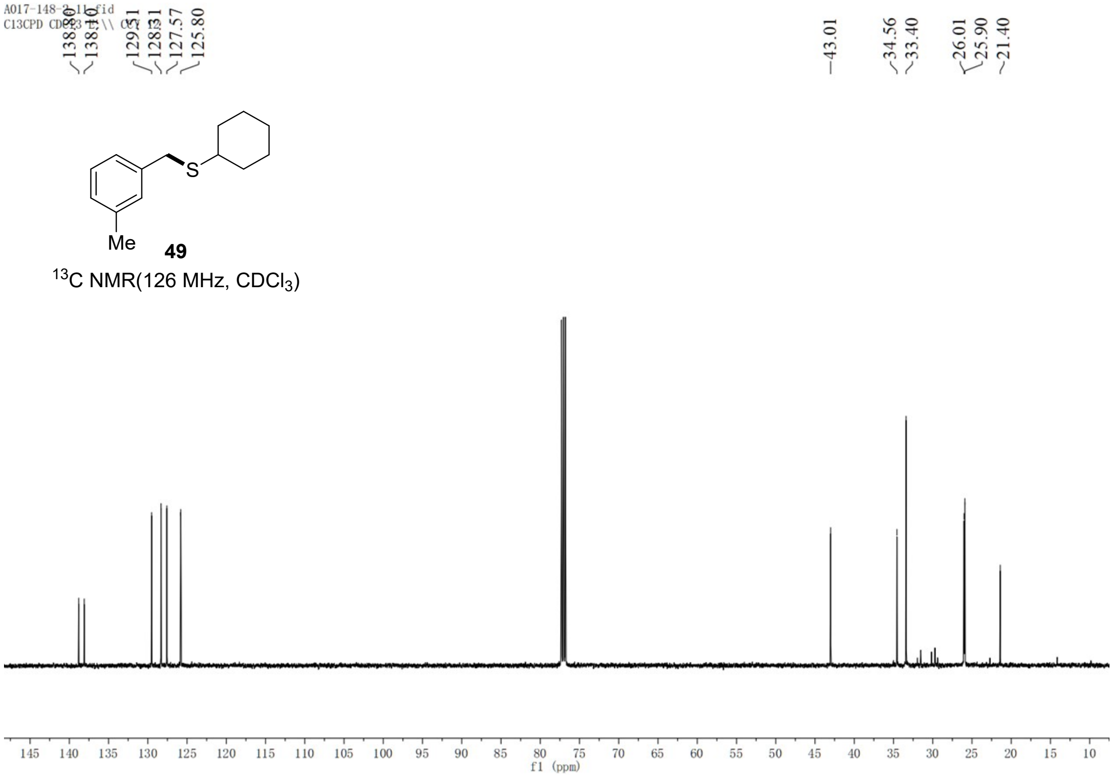

49

^{13}C NMR(126 MHz, $CDCl_3$)

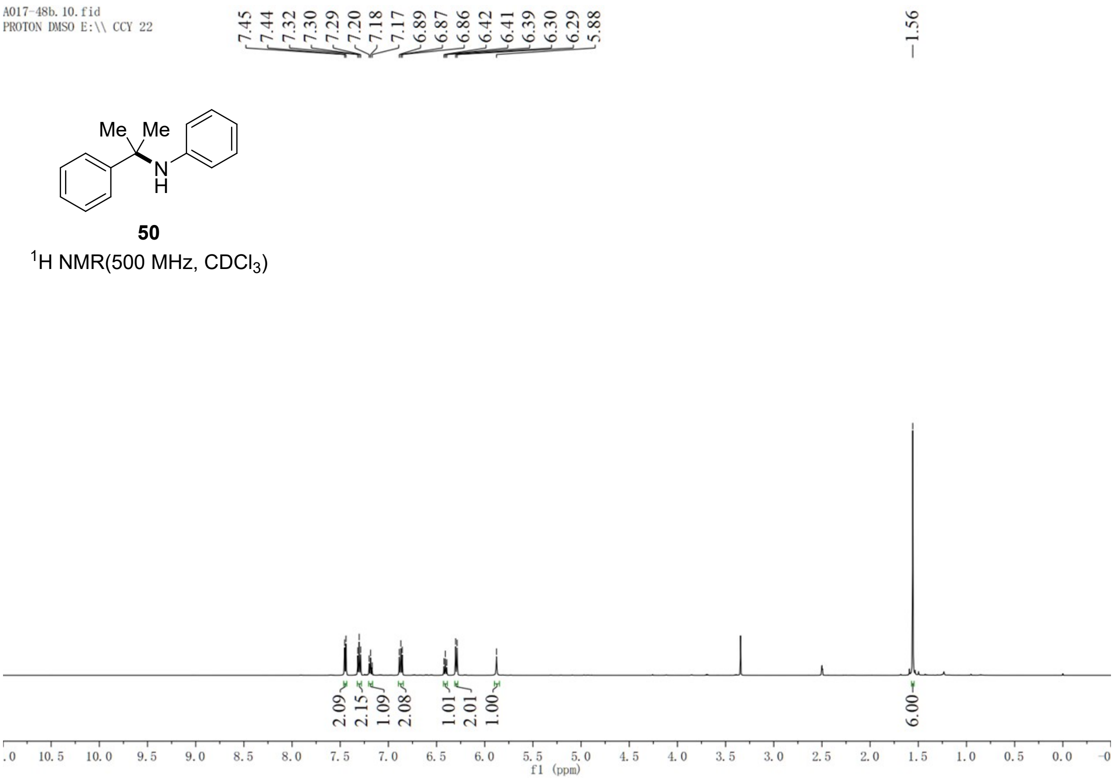

50

^{1}H NMR(500 MHz, $CDCl_3$)

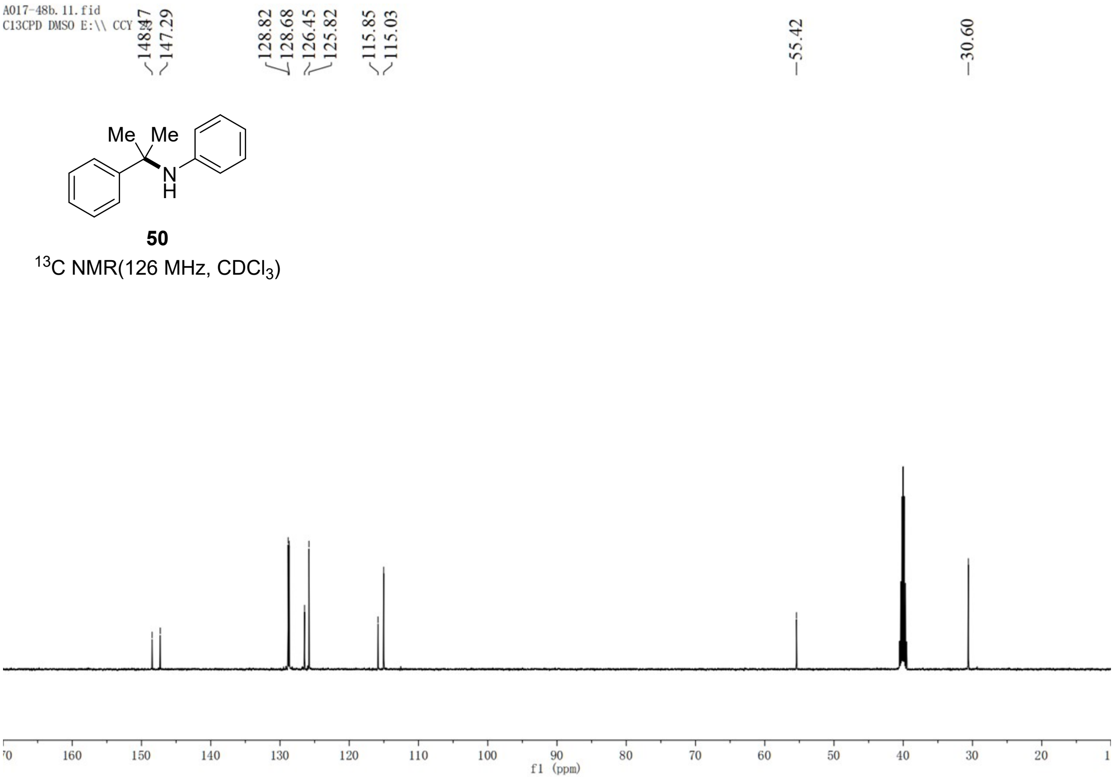

50

^{13}C NMR(126 MHz, $CDCl_3$)

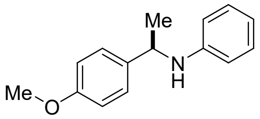

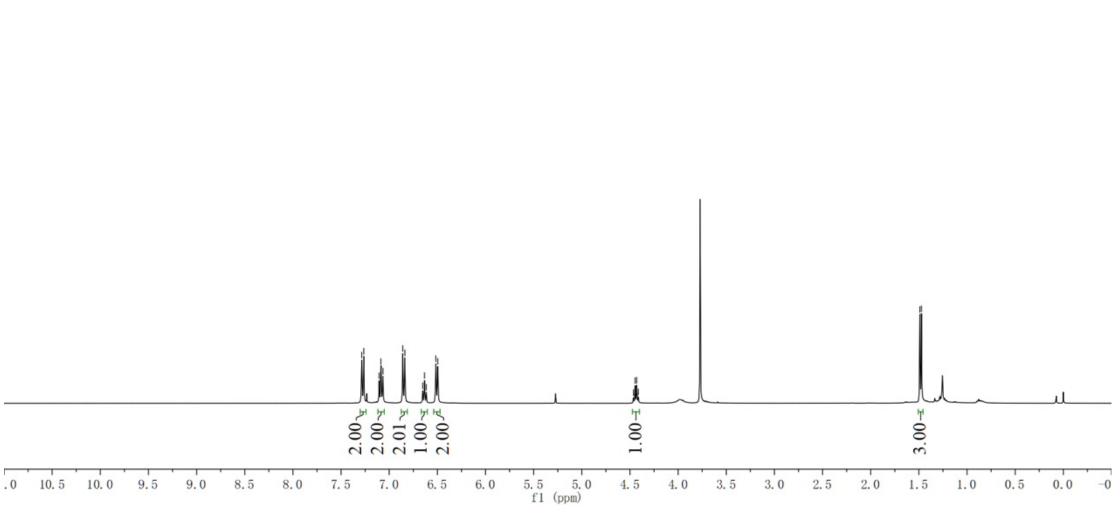

51

^{1}H NMR(500 MHz, $CDCl_3$)

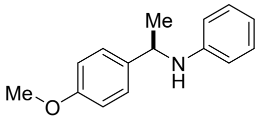

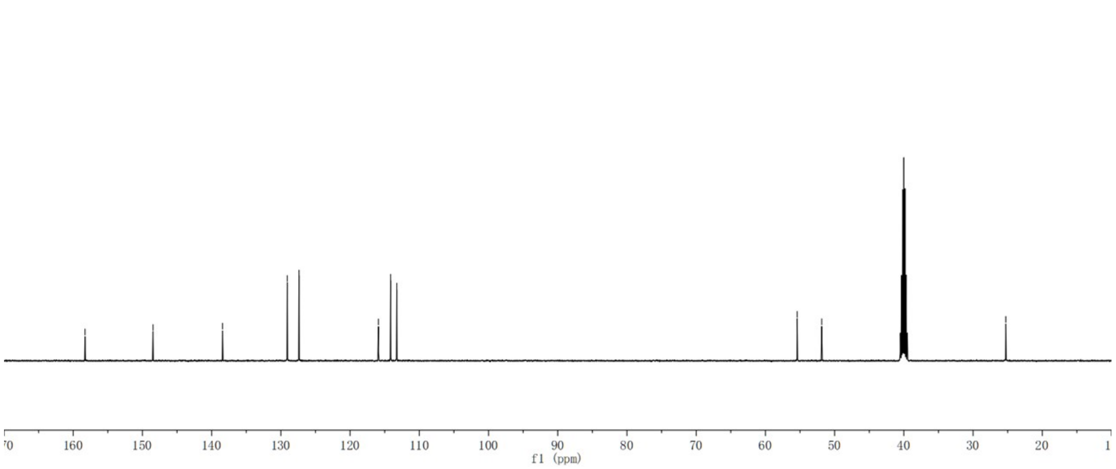

51

^{13}C NMR(126 MHz, $CDCl_3$)

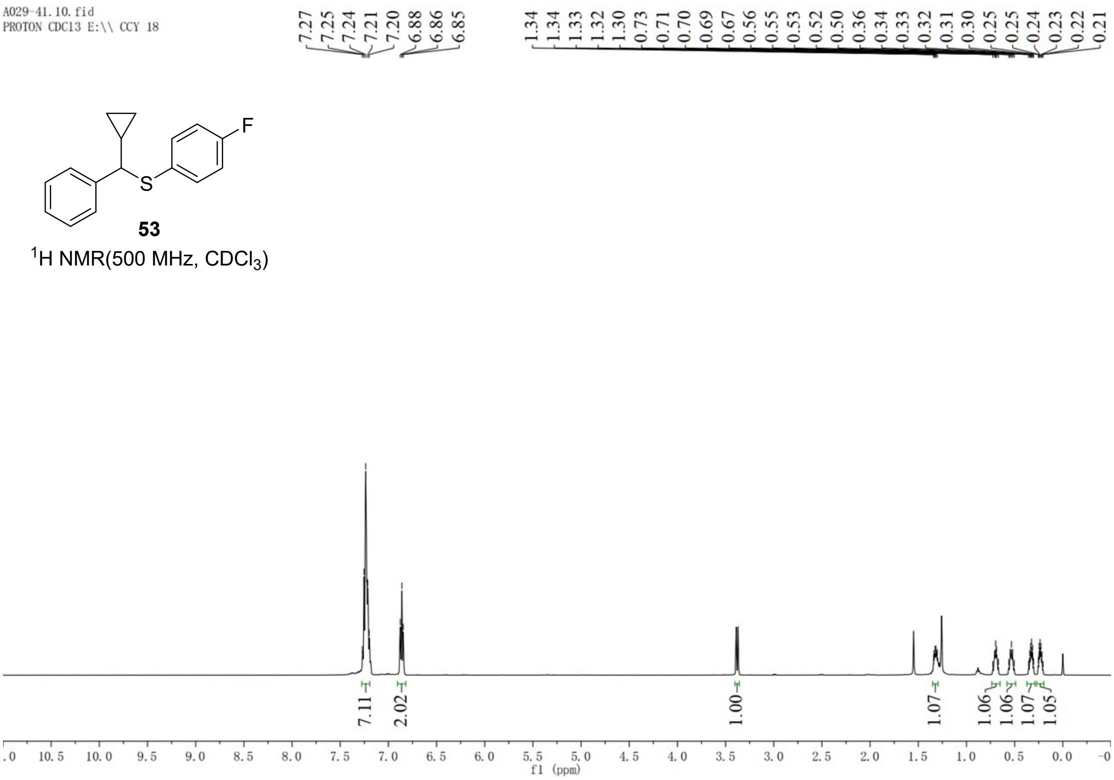

53

^{1}H NMR(500 MHz, $CDCl_3$)

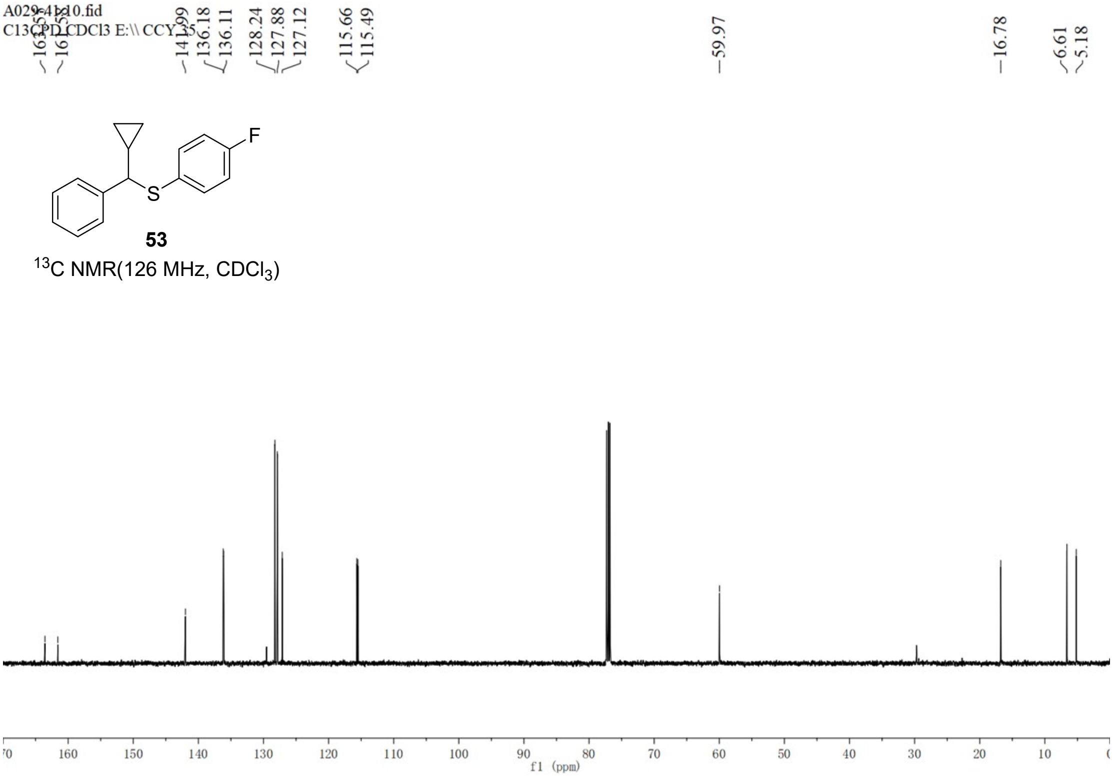

53

^{13}C NMR(126 MHz, $CDCl_3$)

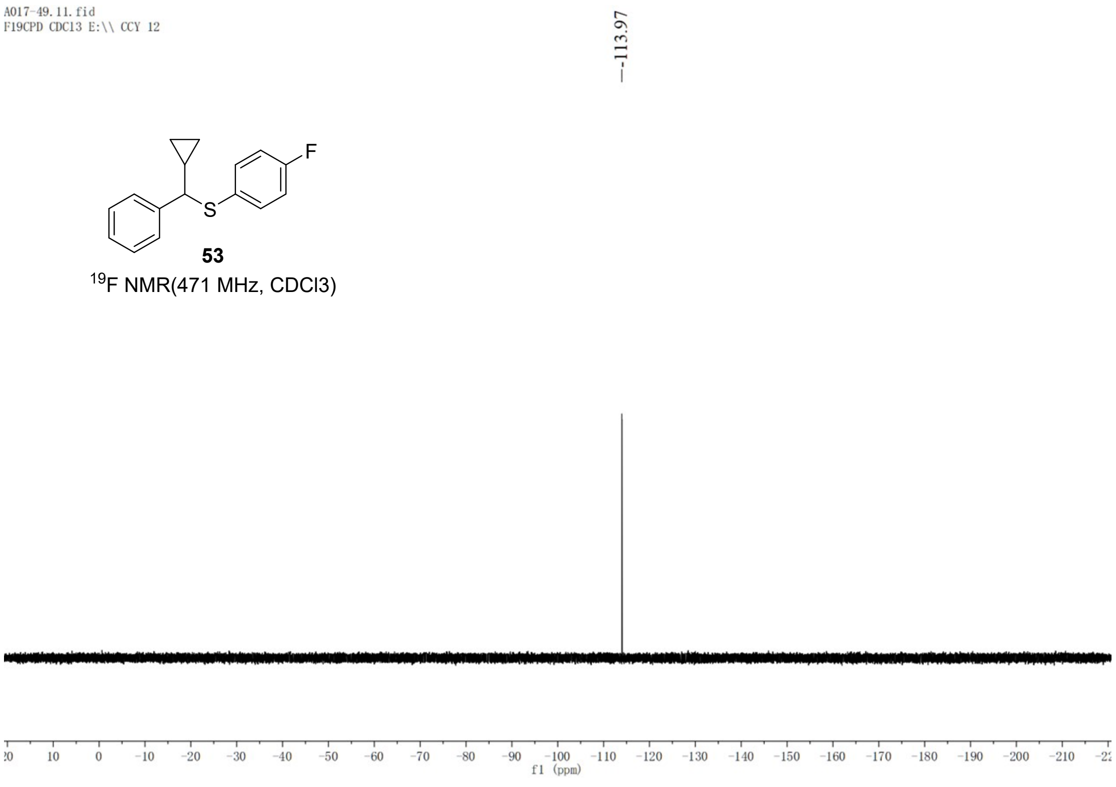
A017-49.11.fid
F19CPD CDCl3 E:\\ CCY 12
--113.97
F
S
53
19F NMR(471 MHz, CDCl3)
20 10 0 -10 -20 -30 -40 -50 -60 -70 -80 -90 -100 -110 -120 -130 -140 -150 -160 -170 -180 -190 -200 -210
f1 (ppm)

2. Spectroscopic Data (HPLC Trace)

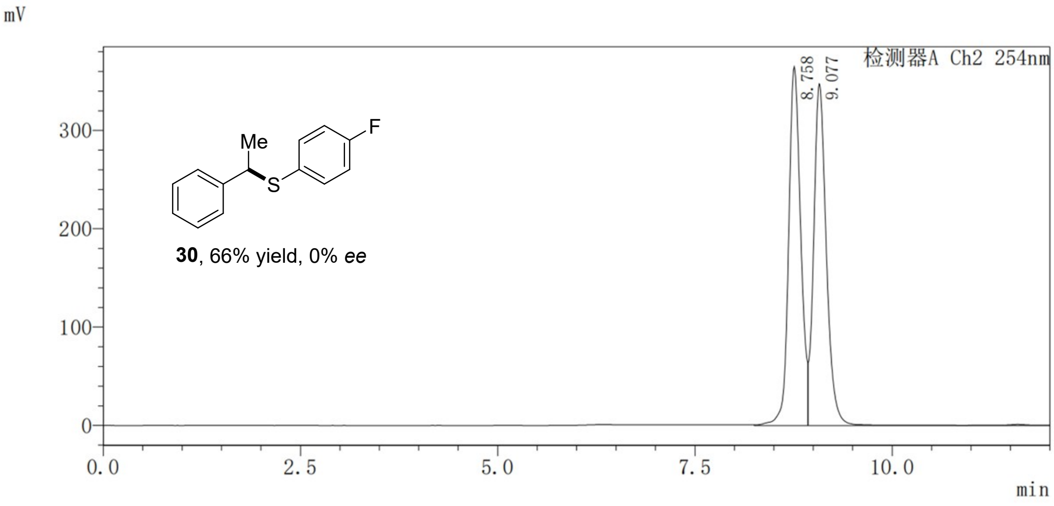

PDA Ch2 254nm

Peak#	Resolution Time	Area	Height	Area %	Height %
1	8.758	3910792	364791	49.622	51.219
2	9.077	3970440	347423	50.378	48.781
Total		7881232	712214	100.000	100.000

III. References

[1] Shelby, Q.; Kataoka, N.; Mann, G.; Hartwig, J. *J. Am. Chem. Soc*. **2000**, *43*, 10718.

[2] Eichman, C. C.; Stambuli, J. P. *J. Org. Chem.* **2009**, *10*, 4005.

[3] Murata, M.; Buchwald, S. L. *Tetrahedron* **2004**, *34*, 7397.

[4] (a) Ye, Y.; Chen, H.; Sessler, J. L.; Gong, H. *J. Am. Chem. Soc.* **2019**, *141*, 820. (b) Ma, G.; Chen, C.; Talukdar, S.; Zhao, X.; Lei, C.; Gong, H. *Chem. Commun.* **2020**, *56*, 10219. (c) Gong, Y.; Zhu, Z.; Qian, Q.; Tong, W.; Gong, H. *Org. Lett.* **2021**, *23*, 1005.

[5] Pijper, T. C.; Robertus, J.; Browne, W. R.; Feringa, B. L. *Org. Biomol. Chem*. **2014**, *1*, 265.

[6] Okamoto, S.; Tsujioka, H.; Sudo, A. *Chem. Lett.* **2018**, *3*, 369.

[7] Jiang, W.; Li, N.; Zhou, L.; Zeng, Q. *ACS Catal.* **2018**, *8*, 9899.

[8] Narobe, R.; Simon J.; Iskra, J.; Burkhard, K. *Adv. Synth. Catal*. **2019**, *361*, 3998.
